# Impacts of allopolyploidization and structural variation on intraspecific diversification in *Brassica rapa*

**DOI:** 10.1186/s13059-021-02383-2

**Published:** 2021-05-31

**Authors:** Xu Cai, Lichun Chang, Tingting Zhang, Haixu Chen, Lei Zhang, Runmao Lin, Jianli Liang, Jian Wu, Michael Freeling, Xiaowu Wang

**Affiliations:** 1 Institute of Vegetables and Flowers, Chinese Academy of Agricultural Sciences, No.12, Haidian District, Beijing, 100081 China; 2 Department of Plant and Microbial Biology, University of California, Berkeley, CA USA

## Abstract

**Background:**

Despite the prevalence and recurrence of polyploidization in the speciation of flowering plants, its impacts on crop intraspecific genome diversification are largely unknown. *Brassica rapa* is a mesopolyploid species that is domesticated into many subspecies with distinctive morphotypes.

**Results:**

Herein, we report the consequences of the whole-genome triplication (WGT) on intraspecific diversification using a pan-genome analysis of 16 de novo assembled and two reported genomes. Among the genes that derive from WGT, 13.42% of polyploidy-derived genes accumulate more transposable elements and non-synonymous mutations than other genes during individual genome evolution. We denote such genes as being “flexible.” We construct the *Brassica rapa* ancestral genome and observe the continuing influence of the dominant subgenome on intraspecific diversification in *B. rapa*. The gene flexibility is biased to the more fractionated subgenomes (MFs), in contrast to the more intact gene content of the dominant LF (least fractionated) subgenome. Furthermore, polyploidy-derived flexible syntenic genes are implicated in the response to stimulus and the phytohormone auxin; this may reflect adaptation to the environment. Using an integrated graph-based genome, we investigate the structural variation (SV) landscapes in 524 *B. rapa* genomes. We observe that SVs track morphotype domestication. Four out of 266 candidate genes for Chinese cabbage domestication are speculated to be involved in the leafy head formation.

**Conclusions:**

This pan-genome uncovers the possible contributions of allopolyploidization on intraspecific diversification and the possible and underexplored role of SVs in favorable trait domestication. Collectively, our work serves as a rich resource for genome-based *B. rapa* improvement.

**Supplementary Information:**

The online version contains supplementary material available at 10.1186/s13059-021-02383-2.

## Introduction

Polyploidization plays a positive role in increasing the richness of the plant kingdom by supporting plant speciation through frequent and recurrent polyploidization and re-diploidization events [1–5]. Previously, Cheng et al. [6] reviewed 49 paleopolyploidies located in the lineages of the plant phylogenetic tree [6], and many more will undoubtedly be discovered due to the increasing number of sequenced plant species. Polyploidization is generally divided into two categories: autopolyploidization, duplication of the same genome; allopolyploidization, merging of two diverged genomes into a common nucleus [7]. Subgenome dominance is a common phenomenon that is widely observed in allopolyploids, including cotton [8], *Brassica* [9], and wheat [10]. This subgenome dominance reflects gene fractionation bias and expression dominance between homoeologous genes from different subgenomes [11–13]. Depending on subgenome location, genes are subjected to subgenome-specific epigenetic regulation [14, 15], altered gene expression and nearby transposon density [16, 17], and frequency of homoeologous chromosome exchange [18, 19].

In addition to the crucial role of allopolyploidization on speciation, allopolyploidization also contributes to species diversification [20], crop domestication [21], and adaptation [22], all of which are attributed to the enhanced genomic plasticity and “evolvability” generated by genome polyploidy [23]. This “evolvability” is thought to derive from the relaxation of purifying selection on any one duplicate gene or subgenome. Intraspecific diversity is the basis of crop domestication but how exactly this diversity contributed to domestication needs to be explored specifically for each cultivar. Therefore, de novo assemblies of multiple, representative genomes for a species should provide new insights for exploring the impacts of allopolyploidization and subgenome dominance on intraspecific diversification.

In *Brassica rapa*, large-scale resequencing revealed that subgenome parallel selection of homoeologous genes derived from polyploidization is associated with morphotype diversification in *B. rapa* and *Brassica oleracea* [24]. In the maize genome, genes in the dominant subgenome explain more important trait variants [25]. In the cotton genome, domestication analysis for long white fibers revealed 620 homoeologous pairs that have been subjected to domestication selection in the A or D subgenome, and only 34 homoeologous pairs exhibit selection signals in both subgenomes, indicating that the coexisting subgenomes have been under asymmetrical domestication selection [26]. However, the combination of a single reference genome and population-scale short-read resequencing cannot be adopted to identify large structural variants and genomic sequences that are absent in the reference genome.

Increasing studies have suggested that single-reference genome is not sufficient to capture all or even most of the variants in a species [27–31], including variants known to have been favored by breeders [27, 28, 32]. A pan-genome represents an approximation of the entire gene repertoire of a species. It was first proposed in bacterial research. The pan-genome concept was quickly applied to studies of human and plant genomes [27, 30, 33–37]. Specifically, the constructions of pan-genomes of some important crops, such as rice, soybean, tomato, and rapeseed, have added completeness to the reference genome and have resolved the full spectrum of variation for a species [27, 28, 38, 39]. In the rapeseed pan-genome, 7.71–14.8% of each genome sequence was absent in the reference genome, and these regions were associated with 2.72–5.04% of species genes [28]. In the soybean pan-genome, 5.75–14.09% of each genome sequence was absent in the reference genome [27]. Additionally, in the *Arabidopsis thaliana* pan-genome, 10.6–14.3% of each genome sequence was rearranged and 4.3–5.3% of each genome sequence was absent in the reference genome, which introduces copy-number changes in ~5000 genes, including ~1900 non-reference genes [40]. Furthermore, the *Brachypodium distachyon* pan-genome contains nearly twice the number of genes found in any individual genome [41]. The pan-genome has been proven to be an excellent tool for revealing extensive genomic variants in plants.

The species *B. rapa* (AA, 2n=20) is one of the most economically important *Brassica* species and is mainly cultivated as a vegetable crop worldwide. Additionally, *B. rapa* is a vital member of the well-established “triangle of U” model [42], providing one of the ancestor genomes of oil-used *Brassica napus* (AACC, 2n=38) and vegetable-used *Brassica juncea* (AABB, 2n=36). As a mesopolyploid crop, *B. rapa* evolved from a translocation Proto-Calepineae Karyotype (tPCK) ancestor and has experienced a whole-genome triplication (WGT) event [43, 44], and the “two-step theory” was proposed to explain the meso-triplication of the *Brassica* “A” genome and the dominant subgenome in the extant diploid genome [16]. During diversification, *B. rapa* formed different subspecies and varieties with highly diverse morphotypes, such as leafy heads, enlarged organs, and extensive axillary branching [24]. Owing to its agronomic importance and evolutionary characteristics, *B. rapa* provides a powerful reference to understanding the unknown impacts of polyploidization and subgenome dominance on intraspecific diversification.

Structural variation (SV) plays an important role in plant evolution and agriculture, by regulating flowering time, stress resistance, fruit flavor, size, and productivity [32, 45, 46]. Currently, the representative reference genomes of *B. rapa* are limited and include Chinese cabbage (ssp. *pekinensis*) [31], yellow sarson (ssp. *tricolaris*) [47], and pak choi (ssp. *chinensis*) [48], which is insufficient to represent major variants among different *B. rapa* genomes, particularly for SVs such as large deletions, inversions, and translocations. This leaves the vast majority of SVs poorly resolved and their impacts on the *B. rapa* genome and phenotypes largely hidden [49]. By exploring single nucleotide polymorphisms (SNPs), Cheng et al. [24] identified six *B. rapa* genes that were strongly selected in the leaf-heading morphotype. However, there are no studies of morphotype domestication using SVs in *B. rapa*.

In this study, we de novo assembled 16 representative *B. rapa* genomes. These genomes together with the two published high-quality reference genomes (Chiifu and Z1) were used to construct a *B. rapa* pan-genome. Based on the *B. rapa* pan-genome, we defined the core and dispensable genes for *B. rapa* and identified SVs. Core genes are defined as genes that were retained in all *B. rapa* genomes, and dispensable genes are defined as genes that were fractionated in some *B. rapa* genomes. We observed that biased gene flexibility, which describes biased gene fractionation during intraspecific diversification, was positively correlated with the extent of subgenome dominance. Furthermore, we constructed an integrated graph-based genome and genotyped SVs in 524 *B. rapa* genomes, thus revealing the SVs involved in morphotype domestication. Specifically, four candidate genes were speculated to be involved in leafy head domestication.

## Results

### De novo genome assembly and annotation of 16 representative genomes indicated variants that were absent in the reference sequences

We individually de novo assembled and annotated 16 representative *B. rapa* genomes. All 16 *B. rapa* accessions were selected from our previous studies [24], including morphotypes of Chinese cabbage, turnip, oilseed, taicai, mizuna, and pak choi (pak choi, wutacai, caixin) (Additional file 3: Table S1). All 16 accessions were de novo assembled using Illumina and PacBio reads (Additional file 3: Table S2), resulting in contig N50 sizes of 0.25–1.41 Mb and genome sizes of 337–466 Mb (Table 1 and Additional file 3: Table S3). To anchor the contigs of each accession to the 10 pseudo-chromosomes of *B. rapa*, 12 of the 16 accessions with relatively higher contig N50 values were sequenced with Hi-C technology; a procedure that aids in assembly [50]. The contigs of the 12 accessions were corrected, ordered, and oriented using 973.05 Gb Hi-C reads in total (Additional file 3: Table S4). The contigs of the remaining four accessions were oriented using reference-guided scaffolding. Further evaluations revealed the high accuracy and completeness of these assemblies (Additional file 1: Supplementary note).

**Table 1:** Assembly and annotation metrics of the 18 *B. rapa* genomes

Accession	Assembly size (Mb)	Complete BUSCOs (%)	Repeat sequences		Annotated loci	Syntenic genes*	
			Length (Mb)	Percent (%)		Count	Percent (%)
BRO	378.31	97.3	183	48.36	46,034	39,689	82.73
CCA	341.62	98.0	153	44.75	46,875	41,599	87.49
CCB	378.47	98.1	187	49.49	44,207	40,379	84.22
Chiifu	353.14	98.0	164	46.52	46,878	46,878	100.00
CXA	378.75	97.8	183	48.27	45,145	39,891	83.30
CXB	348.22	97.9	157	45.12	45,911	40,540	84.27
MIZ	386.27	97.6	186	48.20	45,350	40,083	82.82
OIA	434.64	97.4	221	50.93	45,776	39,394	82.53
OIB	354.41	97.5	164	46.39	46,553	39,672	82.29
OIC	337.46	94.7	154	45.73	45,389	38,606	81.46
PCA	367.56	97.3	177	48.20	46,550	40,848	85.18
PCB	389.26	97.5	187	48.12	46,687	40,227	84.60
TCA	371.28	97.5	182	49.11	46,420	40,582	84.69
TUA	368.70	97.5	174	47.12	47,557	40,287	84.18
TUE	384.72	96.8	193	50.28	45,845	39,927	84.01
TBA	422.86	97.5	217	51.32	45,346	39,105	81.59
WTC	466.50	97.5	254	54.37	47,602	39,225	82.51
Z1	401.93	97.7	179	44.42	46,721	37,630	79.81

*Syntenic genes in each accession were calculated using Chiifu as query genome

We found that 43.59–53.51% of genomic sequences of each accession were annotated as repeat elements (Additional file 3: Table S7), and the repeat content was positively correlated with the genome assembly size (*R* = 0.99, *P* = 3.8e−16) (Additional file 2: Figure S3). Combining ab initio, homology-based annotations and RNA-seq reads (Additional file 3: Table S8), we detected 44,207–47,602 gene models in each of the 16 genomes. Together with the two reported genomes (Chiifu and Z1) [31, 47], we obtained a total of 18 *B. rapa* de novo assembled genomes in the present study. Using Chiifu as the reference, 79.81–87.49% of the genes were identified as syntenic genes in the other *B. rapa* genomes (Additional file 3: Table 1 and Additional file 3: Table S9).

The 18 *B. rapa* genomes revealed extensive variants that were absent in the Chiifu reference genome. By aligning the 17 genomes to Chiifu individually, we found that 15.14–37.39% of each genome sequence was not syntenic with the Chiifu genome (Additional file 3: Table S10), including 5.63–13.14% of genomic sequences that were absent in the Chiifu genome (Additional file 3: Table S11), revealing that different subspecies/varieties diversified has resulted in major gene content, collinearity, and chromosome-structural differences. Furthermore, based on the resequencing data of the 18 de novo assembled accessions, we identified 2.3–4.9 × 10^6^ SNPs and 0.4–0.9 × 10^6^ InDels by taking each of the 17 assemblies as the reference genome. The number of variants varied greatly using different genomes as references. Specifically, 8.8–17.7% and 5.8–11.53% of SNPs and InDels occurred in non-syntenic regions between each genome and Chiifu, and 4.3–8.9% and 2.9–5.3% of SNPs and InDels were in the regions that were absent in the Chiifu genome (Additional file 3: Table S12).

### Composition and features of the *B. rapa* pan-genome

**Fig. 1:**
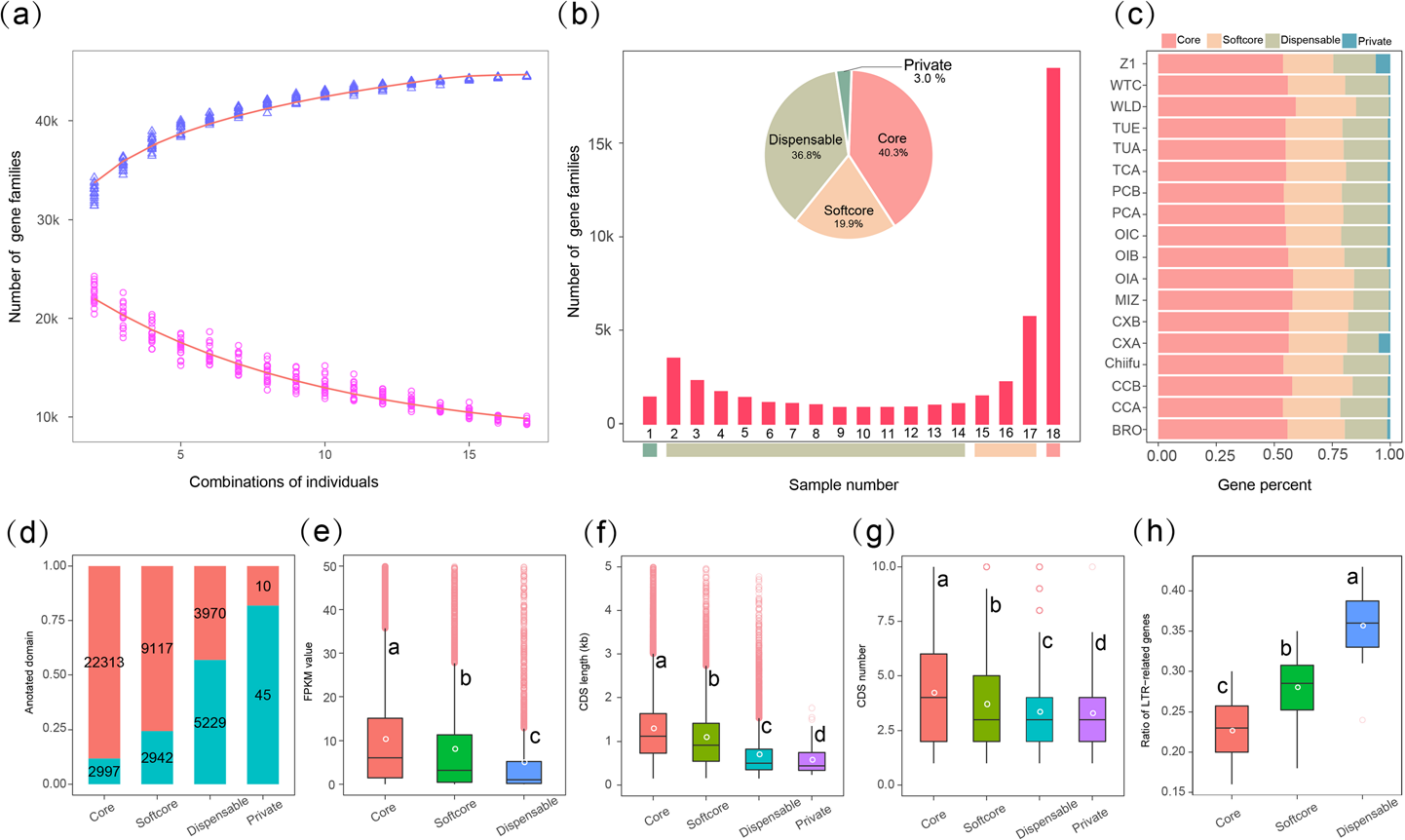
Composition and characteristics of the *B. rapa* pan-genome. **a** Pan-genome models for *B. rapa*. The upper and lower curves show the number of total and core gene families after different combinations of individuals. **b** Compositions of the *B. rapa* pan-genome. The histogram shows the number of gene families in the 18 genomes with different frequencies. The pie chart shows the proportion of the gene families marked by each composition. **c** Number of classified genes in each genome. **d** Proportion of genes with InterPro domains in the Chiifu core genes, softcore genes, dispensable genes, and private genes. Orange and green bars represent genes with InterPro domain annotations and genes without InterPro domain annotations. **e** The expression level of core, softcore, and dispensable genes in the Chiifu genome. **f**, **g** CDS length (**f**) and CDS number (**g**) of each gene in Chiifu core, softcore, dispensable, and private genes. **h** The ratio of LTR-related genes in core, softcore, and dispensable genes. An LTR-related gene is defined as a gene with insertions of LTR-RTs in the regions of 2 kb upstream and downstream of the gene body. The white dots indicate the average value in all figures, and multiple comparisons were performed by the Student-Newman-Keuls test with a = 0.01 (same as presented in Figs. 3 and 4)

We constructed a *B. rapa* pan-genome consisting of the 16 representative genomes and the two published reference genomes [31, 47]. In total, we detected 47,107 gene families in the *B. rapa* pan-genome. Modeling of pan-genome size suggested a closed pan-genome for *B. rapa* species (a closed pan-genome indicates that the additional sequenced genomes do not add new genes into the existing pan-genome)*.* The total gene families increased as additional genomes were added, approaching a plateau when *n* = 16 (Fig. 1a), indicating that the pan-genome represents most of the *B. rapa* species gene content. To further dissect the *B. rapa* pan-genome, we divided all families into different classes according to the frequency of gene families present in the 18 genomes. Of the total gene sets, gene families present in all genomes were defined as core genes, those present in 15 to 17 genomes (more than 80% of all accessions) were defined as softcore genes, those present in two to 14 genomes were defined as dispensable genes, and those present in one accession with homologs and orphan genes (no homologs) were both defined as private genes. In total, an average of 55.74%, 25.00%, 17.80%, and 1.46% of genes in each genome was considered as the core, softcore, dispensable, and private genes, respectively (Fig. 1b–c and Additional file 3: Table S13).

**Fig. 2:**
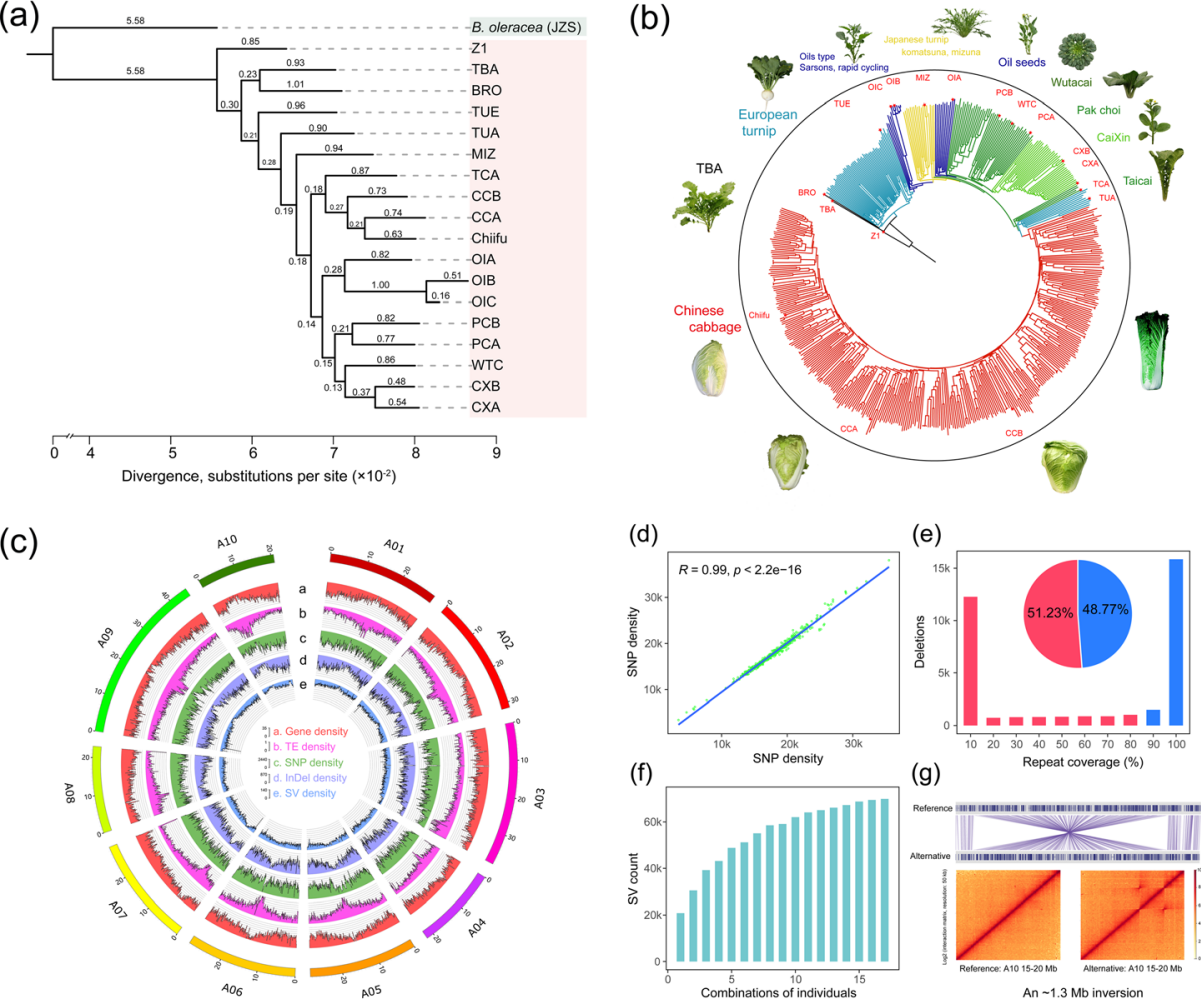
Phylogeny and variation landscapes from 18 *B. rapa* representative genomes and 524 resequenced accession genomes. **a** Phylogenetic relationships of 18 *B. rapa* accessions using *B. oleracea* as an outgroup. The branch length values represent divergence between accessions with *B. oleracea*. **b** A neighbor-joining tree of 524 *B. rapa* accessions. Different colors indicate the accessions within different sub-populations, and the 18 *B. rapa* genomes are specifically marked with red stars. Representative morphological pictures are displayed next to the corresponding sub-populations. **c** Distribution of genomic variants from 18 genomes and 524 accessions on 10 *B. rapa* chromosomes using Chiifu as the reference. **d** Correlation between SNP densities detected by resequencing data of the *B. rapa* germplasm (*x*-axis) and comparison of de novo assemblies (*y*-axis). We calculated the number of SNPs in a bin with a size of 500 kb. **e** SV number plots against repetitive sequences. We used deletion sequences to evaluate the correlation between SV and repetitive sequences involvement. Red (blue) indicates that the proportion of repetitive sequences in the SV sequence is less (greater) than 80%. **f** The number of SVs after different combinations of individuals. **g** An ~1.3 Mb inversion specifically occurred in the Chiifu and CCB genomes

Intraspecific diversification mainly occurred in dispensable and private genes. The proportion of core genes with large effect mutations was significantly lower than that in dispensable and private genes, especially in private genes, where the ratio of genes with large effect mutations was almost three times that in the core genes (on average, 11.41% and 33.44% of the core and private genes were detected, respectively) (Additional file 2: Figure S5 and Additional file 1: Supplementary note). Additionally, we found that 88.2% of core genes and 75.6% of softcore genes in the Chiifu genome contained InterPro domains, which was much higher than either that of the dispensable or private genes (43.2% and 18.2% for dispensable and private genes, respectively) (Fig. 1d). The average expression level of core genes was also significantly higher than that of dispensable genes (Fig. 1e). The average length and number of core gene CDSs were significantly higher than that of less conserved categories (Fig. 1f–g). Additionally, we observed that genes with LTR-RTs inserted within the transcriptional unit exhibited significantly lower expression levels (*P* = 9.8e−08) (Additional file 1: Supplementary note; Additional file 2: Figure S8). The insertions of LTR-RTs were more likely to occur in the dispensable genes (Fig. 2h), indicating that the dynamics of TE insertion in dispensable and private genes accelerated genetic variants during intraspecific diversification.

### The pan-genome reveals comprehensive variation and enormous structural complexity within the *B. rapa* species

The outline of the *B. rapa* phylogeny constructed using single-copy nuclear genes across the genome showed extensive phylogenetic discordance. To infer the ordinal phylogeny of the different *B. rapa* morphotypes, we selected 7900 single-copy nuclear genes and inferred a single-species phylogeny for the 18 *B. rapa* accessions with one *B. oleracea* accession (JZS v2.0) [51] as an outgroup (Fig. 2a). The results revealed that *B. rapa* morphotypes were clearly divided into turnip, oil type, pak choi, and Chinese cabbage, etc. However, we noticed that the tree inferred from variants on each chromosome was not fully consistent with the genome-wide phylogenetic tree (Additional file 2: Figure S9), illustrating a complex history of intraspecific diversification. For example, data for chromosomes A02, A08, and A10 suggested that OIB and OIC (ssp. *tricolaris*) were closest to the *B. rapa* ancestral branch. However, OIB and OIC were the sister group of OIA, as inferred from the whole-genome tree.

There is a strong correlation between the variants identified by large-scale resequencing accessions and the *B. rapa* pan-genome. We produced and collected resequencing data from a natural population consisting of 524 diverse *B. rapa* accessions (Additional file 1: Supplementary note). In total, we detected 3,971,130 SNPs and 1,144,753 InDels with minor allele frequency (MAF) ≥ 0.05. The neighbor-joining tree of 524 *B. rapa* accessions showed that the 16 accessions we chose for our pan-genome analysis existed, as expected, in different sub-populations, each with distinctive morphotypes (Fig. 2b). Additionally, there was a strong correlation between the SNPs detected through assembly-calls of the 17 de novo assemblies using Chiifu as the reference and the SNPs obtained from mapping-calls of 524 resequencing data (*R* = 0.99, *P* < 2.2e-16) (Fig. 2d). Furthermore, we found that 92.82% of SNPs detected by the mapping-calls were present in the SNP data obtained by assembly-calls (Additional file 2: Figure S10), further revealing that the pan-genome harbored abundant variants of *B. rapa*.

SVs in the pan-genome illustrated the enormous structural complexity of *B. rapa*. By investigating variation landscapes in the pan-genome using Chiifu as the reference (Fig. 2c), we detected 33.24–56.7 Mb insertions and 35.75–58.84 Mb deletions (size ≥ 50 bp; Additional file 3: Table S18). Additionally, frequent translocations and inversions were also detected (Additional file 1: Supplementary note). Furthermore, we used insertions and deletions (size ≥ 50 bp) as representatives to investigate SV characteristics in different *B. rapa* genomes. We observed that SVs tended to be enriched in repetitive sequences (Fig. 2e and Additional file 2: Figure S11), and SVs were tightly associated with morphological diversification (Additional file 1: Supplementary note). Similar to the patterns of core and dispensable gene families (Fig. 1a), along with the increasing number of genomes, the addition of more sequenced genomes did not affect the size of the nonredundant SV set (Fig. 2f). Notably, we detected four large inversions with sizes of 1.74 Mb, 1.65 Mb, 1.3 Mb, and 0.99 Mb verified by the Hi-C data (Fig. 2g, Additional file 2: Figure S17 and Additional file 3: Table S21). These results highlighted the enormous structural complexity in *B. rapa* during intraspecific genome diversification.

### The flexibility of syntenic genes was associated with the diversification of different *B. rapa* genomes

**Fig. 3:**
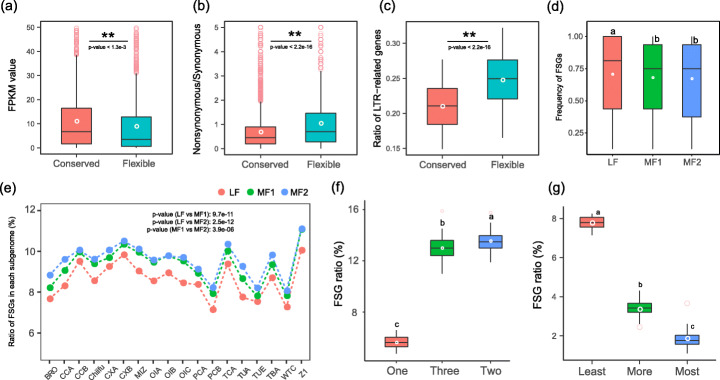
Gene flexibility increased during the period of intraspecific genome diversification in *B. rapa*. **a** Comparison of the expression levels of homoeologous pairs consisting of conserved and flexible syntenic genes in the Chiifu genome. “Conserved” and “Flexible” represent conserved and flexible syntenic gene in the homoeologous pair. The *P* value was calculated based on a paired t test. **b** The ratio of nonsynonymous to synonymous SNPs in CSGs and FSGs. **c** The ratio of LTR-related genes in CSGs and FSGs. **d** Frequencies of FSGs in the three *B. rapa* subgenomes. **e** Ratio of FSGs in the three subgenomes of the 18 *B. rapa* genomes. **f** The ratio of FSGs in single-, two-, and three-copy gene sets of the 18 genomes. **g** Ratio of least, more, and most flexible syntenic genes in the three-copy genes

A large number of polyploidy-derived genes were observed to be fractionated in the different *B. rapa* genomes. Here, we more specifically defined conserved syntenic genes and flexible syntenic genes to further explore the evolution of genes derived from polyploidy during intraspecific genome diversification. In this study, if a gene had an ortholog in *A. thaliana* and was present in more than 16 of 18 genomes, then the gene was defined as a conserved syntenic gene (CSG); if a gene had an ortholog in *A. thaliana* and was present in two to 16 genomes, then the gene was defined as a flexible syntenic gene (FSG) (Additional file 2: Figure S18). We used this strict standard to ensure that each CSG and FSG was supported by at least two sequenced genomes. By calculating syntenic gene arrays for *A. thaliana* and our 18 genomes, we identified 24,411–25,132 (3324–4575) CSGs (FSGs) in each of the 18 *B. rapa* genomes (Additional file 3: Table S22). On average, 13.42% of WGT-derived genes were flexible among the sequenced accessions. Additionally, we found that the average of expression level of the FSGs was significantly lower than that of the CSGs (Fig. 3a).

To further investigate the evolutionary characteristics of these FSGs, we calculated SNPs in the FSGs and CSGs, and the results revealed that the average ratio of nonsynonymous to synonymous SNPs in FSGs was significantly higher than that in CSGs (*P* < 2.2e−16) (Fig. 3b), which indicates that FSGs were prone to non-synonymous mutations. Similarly, we calculated genes with large effect mutations in the CSGs and FSGs, which indicated that FSGs harbored a significantly higher content of genes with large effect mutations (such as start-codon mutation, stop-codon mutation, and premature stop codon) [52] in the pan-genome (*P* = 2.6e−29) (Additional file 3: Table S23). Meanwhile, we also observed that FSGs accumulated significantly higher proportions of SVs than CSGs (*P* = 0.1e−15) (Additional file 2: Figure S19). Additionally, the genic region (the regions of the gene body and 2-kb flanking sequences) of the FSGs harbored significantly more LTR-RTs than CSGs (*P* < 2.2e−16) (Fig. 3c). In summary, our observation of the high percentage of FSGs and the higher tendency of accumulation of non-synonymous mutations, large-effect mutations, SVs, and LTR-RTs indicated that FSGs were strongly correlated with the diversification of different *B. rapa* genomes.

### Gene flexibility during intraspecific diversification exaggerated the dominance of the LF subgenome

We found that gene flexibility, during intraspecific diversification, was biased to the more fractionated subgenomes (MFs). After WGT, significant subgenome dominance was observed in the extant mesohexaploid *B. rapa* genome. Previously, subgenome dominance was explained by the “two-step theory,” which suggests that *B. rapa* experienced a tetraploidization followed by fractionation and subsequent hybridization with a third genome, which shows less fractionation [16]. However, the evolution of the dominant subgenome during intraspecific diversification is unexplored. The present study found that the average ratio of FSGs on the LF, MF1, and MF2 subgenomes was 8.57%, 9.27%, and 9.55%, respectively, and the ratio of FSGs was significantly lower in the LF subgenome (Fig. 3e, Additional file 2: Figure S20 and Additional file 3: Table S24), revealing that the biased gene flexibility during intraspecific diversification was associated with the increase of the dominance of the LF subgenome. Meanwhile, we calculated the presenting frequency of each FSG in the 18 genomes. When comparing the frequency of FSG among the three subgenomes, we observed a significantly higher value in the LF subgenome than in the other two MF subgenomes (Fig. 3d), further highlighting the continuing influence of biased gene flexibility during intraspecific diversification.

The gene flexibility, which was biased to multi-copy genes, was associated with environmental adaptation. We found that the ratios of FSGs in single-, two-, and three-copy gene sets were 5.61%, 13.53%, and 12.98% on average, respectively (Fig. 3f and Additional file 3: Table S25), and the ratio of FSGs in the multi-copy gene sets was more than twice that of the single-copy gene set in each of the 18 genomes, illustrating that the multi-copy genes were more likely to be flexible during intraspecific diversification. In the present study, if one gene was a flexible syntenic gene in the two or three copies, then the flexible syntenic gene was further defined as the least FSG. Here, “least” means one copy of the gene in the genome. If two and three genes were FSGs, they were defined as “more” and “most” FSGs, respectively. We found that an average of 10.06% and 3.47% of two copies were least and more FSGs (Additional file 2: Figure S21), and an average of 7.77%, 3.35%, and 1.86% of three copies were least, more, and most FSGs, respectively (Fig. 3g and Additional file 3: Table S26). The results revealed the high flexibility of multi-copy genes during intraspecific diversification.

The Gene Ontology (GO) enrichment categories of these least, more, and most FSGs revealed that all three types of genes were enriched in terms of response to stimulus, cellular developmental process, and response to auxin (GO:0009733 and GO:0009725; Additional file 3: Table S27–S29), suggesting that these genes were associated with environmental adaptation, as responses to stimulus and the phytohormone auxin are critical for adaptation and plant growth [53].

### The *B. rapa* inferred ancestral genome provides new insights for systematically investigating intraspecific diversification

**Fig. 4:**
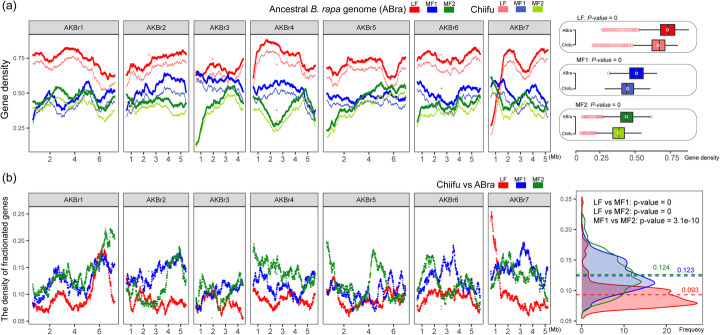
The dominance of the LF subgenome during intraspecific diversification in *B. rapa*. **a** Gene density in the three subgenomes of the inferred *B. rapa* ancestral genome and Chiifu genome. The inferred *B. rapa* ancestral genome was constructed using the 18 genomes with *A. thaliana* as the reference. The Chiifu genome is used as a representative to illustrate intraspecies diversification in *B. rapa*. Gene density was calculated based on an ancestral karyotype of Brassiceae (AKBr). **b** The density of fractionated genes during the formation of the Chiifu genome. The x-axis indicates the seven inferred chromosomes of the inferred *B. rapa* ancestral genome based on AKBr. The y-axis indicates the ratio of fractionated genes to the genes in each bin of the inferred ancestral genome during the formation of the Chiifu genome. A 500-gene sliding window with an increment of two genes was adopted to calculate the gene density in **a** and **b**. The figure on the right shows the distribution of ratios of fractionated genes to the genes in each bin of the inferred ancestral genome, and the dotted line represents the average of these ratios in each subgenome

We constructed an inferred ancestral genome of *B. rapa* by merging all the genes of the 18 *B. rapa* genomes, which were syntenic with *A. thaliana*, and ordering them as the tPCK karyotype (Methods; Additional file 4). In total, there were 30,166 genes in the inferred *B. rapa* ancestral genome, of which 13,116, 9182, and 7868 genes were in the LF, MF1, and MF2 subgenomes, respectively. We then calculated gene densities in the three subgenomes to investigate biased gene fractionation. The average densities of genes in LF, MF1, and MF2 were 0.727, 0.507, and 0.435, respectively (Fig. 4a), indicating the subgenome dominance in the inferred *B. rapa* ancestral genome.

To investigate the individual genome evolution, we compared the Chiifu genome with the inferred *B. rapa* ancestral genome. In the Chiifu genome, the average densities of genes in LF, MF1, and MF2 were 0.661, 0.445, and 0.381, respectively. The average gene density in the individual genome was significantly lower than that of the inferred *B. rapa* ancestral genome (*P* = 0, Fig. 4a), revealing extensive gene fractionation during intraspecific diversification. Moreover, we calculated the distribution of FSGs in the Chiifu genome (Additional file 2: Figure S22) and found that the average densities of fractionated genes in LF, MF1, and MF2 were 0.093, 0.123, and 0.124, respectively (Fig. 4b). The density of fractionated genes in the LF subgenome was significantly lower than in the MF subgenomes (*P* = 0, Fig. 4b), revealing that the genes in LF had a significantly lower fractionation rate than those in the MF subgenomes during intraspecific diversification.

**Fig. 5:**
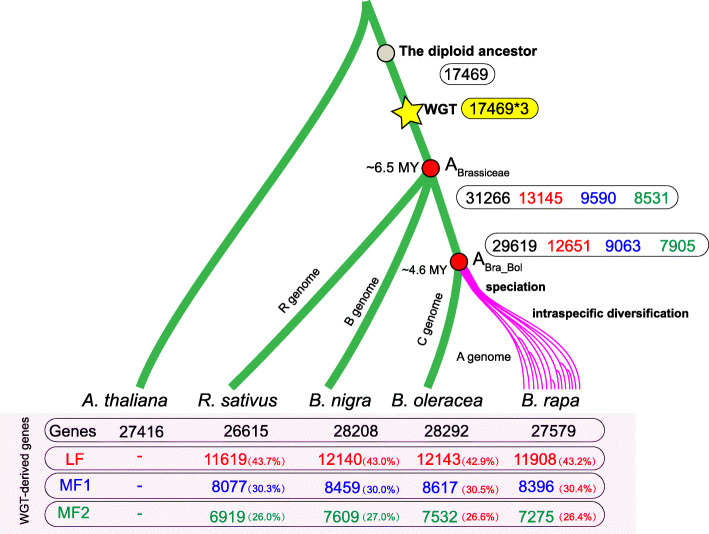
The number of WGT-derived genes in the three subgenomes of Brassiceae species and their inferred ancestral genomes. Red, blue, and green represent the number of genes (gene ratio) in the three subgenomes of the three *Brasica* diploids and *Raphanus sativus*. The red dots represent the inferred ancestral genome of Brassiceae species (A_Brassiceae_) and the ancestral genome of *B. rapa* and *B. oleracea* (A_Bra_Bol_). The pink lines indicate *B. rapa* speciation and intraspecific diversification. This figure is based on information published previously by Cheng et al. [55]

*Brassica*s evolved from the tPCK ancestor genomes before WGT [44, 54], with *Brassica nigra* emerging at about 6.5 MYA (million years ago), followed by the emergence of *B. rapa* and *B. oleracea* at about 4.5 MYA [55]. We reconstructed the ancestral genomes of all Brassiceae species to evaluate the impacts of the dominant subgenome on speciation. Using the same method for constructing the *B. rapa* ancestral genome, we deduced the common ancestral genome of Brassiceae species (A_Brassiceae_) and the common ancestral genome of *B. rapa* and *B. oleracea* (A_Bra_Bol_) (Additional file 5). In total, there were 31,266 (29,619) genes in the inferred A_Brassiceae_ (A_Bra_Bol_) genome, of which 13,145 (12,651), 9590 (9063), and 8531 (7905) genes were in the LF, MF1, and MF2 subgenomes, respectively (Fig. 5). More genes were retained in the dominant subgenome, as reported in Brassiceae species [43, 56–58]. Of course, a lower fractionation rate was observed in the LF subgenome (3.76%, 550%, and 7.34% of A_Brassiceae_ genes were fractionated in the LF, MF1, and MF2 subgenomes), illustrating the out-sized contributions of the dominant subgenomes to *B. rapa* speciation. In summary, together with the observations that significantly fewer FSGs in the dominant subgenome during *B. rapa* intraspecific diversification (Fig. 4b), we have highlighted the continuing influence of the dominant subgenome on the evolution of *Brassica*s.

### The pan-genome enables a broad survey of SV and how SV is linked to morphotype diversity

An integrated graph-based *B. rapa* genome was constructed to investigate SV landscapes in 524 genomes. Based on the alignments of 18 genomes, we obtained a set of 87,032 nonredundant SVs (insertions and deletions; size ≥ 50 bp) and constructed an integrated graph-based genome using Chiifu as the reference. We randomly selected several SVs and used PCR amplification to assure the fidelity of these SVs (Additional file 2: Figure S23 and Additional file 3: Table S31). We then mapped the resequencing data of 524 accessions onto the graph-based genome to genotype all of the candidate SVs. In total, we detected 57,877 SVs (containing 28,052 deletions and 29,914 insertions) with MAF ≥ 0.05, which made it feasible to investigate the relationship between SVs and morphotype domestication of *B. rapa*.

SVs were associated with the domestication of different *B. rapa* morphotypes. Three *B. rapa* outstanding morphotypes of Chinese cabbage, pak choi, and European turnip were selected to investigate the relationship between SV and the domestication of different morphotypes. Generally, if an allele of one SV was enriched in the target morphotype, it indicated that the SV might be related to the target morphotype domestication. Based on this principle, we divided all accessions into heading populations (329 accessions) and non-heading populations (195 accessions), calculating the ratio of two genotypes of each SV in the two populations. In total, 1064 SVs were enriched in the heading population, which were associated with 266 genes in the Chiifu genome, corresponding to 191 orthologous genes in the *A. thaliana* genome (Additional file 3: Table S32). Similarly, 19 and 172 SVs were considered to be closely related to the domestication of pak choi and European turnip morphotypes (Additional file 2: Figures S24–25 and Additional file 3: Tables S33–34). These findings revealed that the SVs were associated with the domestication of different morphotypes. Additionally, we identified a 55-bp SV occurring in the *BrFLC2* gene body between oil-type and other accessions (Additional file 2: Figure S26). The SV was previously reported to only occur in oil-type *B. rapa* and contributed to variation in flowering time [46]. This finding further provided evidence that SV was associated with morphotype domestication in *B. rapa*.

**Fig. 6:**
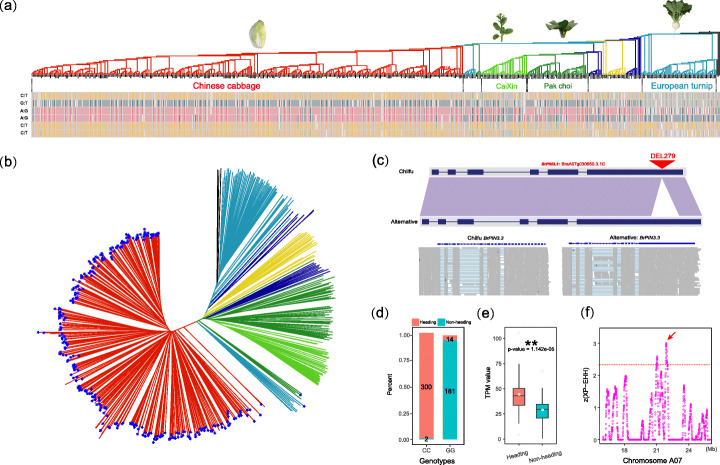
Structural variation in *BrPIN3.3* is associated with *B. rapa* heading morphotype domestication. **a** The distribution of haplotypes in the *BrPIN3.3* gene region in 524 genomes. The numerical suffix denotes a gene’s location on the subgenome LF, MF1, or MF2. Homozygous sites of AA, CC, GG, and TT are filled using different colors as described in the figure, while missing loci (NN) and heterozygous loci (Hetero) are not filled with color. **b** The distribution of one of the *BrPIN3.3* genotypes in 524 accessions. Accessions with a 279 bp deletion in *BrPIN3.3* are marked using blue stars. **c** Micro-synteny analysis between the two genotypes of *BrPIN3.3*. RNA-seq reads of different accessions were collected and mapped onto the two genotypes. **d** The genotype of the structural variation in the *BrPIN3.3* gene region in 524 accessions. CC indicates that the genotype in the corresponding accession was consistent with the reference genome, and GG indicates that the genotype in the accession was different from the reference genome. **e** The expression level in TPM of *BrPIN3.3* in 44 heading and 42 non-heading accessions. CC (Chinese cabbage) and others represent heading and non-heading types, respectively. **f** XP-EHH values are normalized as z-scores for *B. rapa* on A07. A 200-kb sliding window with an increment of 5 kb was used to calculate these normalized XP-EHH values. Each point represents a value in a 200-kb window, and the horizontal dashed line presents the empirical threshold of α = 0.01 (z = 2.33). The arrow indicates the location of the *BrPIN3.3* gene

We identified that *BrPIN3.3*, *BrMYB95.3*, *BrFL5.1*, and *BrSAL4.2* associated with the leafy head domestication. For *BrPIN3.3*, there was a 279-bp deletion that occurred in 300 of 329 heading accessions, while appearing in only two non-heading accessions (Fig. 6b–d, Additional file 2: Figure S27), indicating that the 279 bp deletion was under extremely strong selection and was tightly linked with leaf heading morphotype domestication. Then, we selected SNPs of 524 accessions in the gene region and then joined these SNPs to form the gene haplotype (Fig. 6a). The result indicated that the heading population displayed a uniform haplotype, which was different from the non-heading haplotype. Furthermore, we found that the *BrPIN3.3* gene existed in a putative selective region that had a strong selection signal for leaf heading domestication (Fig. 6f). These findings further confirmed that the *BrPIN3.3* gene was tightly linked to the domestication of the leaf heading morphotype, and alleles of this gene itself are candidate heading domestication genes. In *A. thaliana*, *AtPIN3* encodes putative auxin efflux carrier that is involved in auxin polar transport, response to light stimulus, auxin efflux, and regulation of hormone levels. In *B. rapa*, *BrPIN3.3* is the orthologous gene of *AtPIN3* and plays an essential role in the development of heads [59]. Furthermore, we investigated the expression level of the *BrPIN3.3* gene in 44 heading and 42 non-heading populations. The results showed that the expression level of the *BrPIN3.3* gene with the SV in the heading population was significantly greater than that in the non-heading population (*P* = 1.1e−5). Additionally, we validated the selection signals in a germplasm collection of 884 *B. rapa* accessions (208 heading and 676 non-heading accessions) (Additional file 3: Table S35), which revealed that the four candidate SVs were strongly associated with the leaf-heading trait (*P* < 2.2e−16) (Additional file 2: Figure S28 and Additional file 3: Table S36). The other three candidate genes were also analyzed using the same methods (Additional file 1: Supplementary note).

## Discussion

### Genome composition may be a contributing factor characterizing the dominant subgenome

The existence of a dominant subgenome is widely distributed in allopolyploid species [43, 56, 60–62]. *Brassca rapa* was subjected to a WGT event, providing a crucial reference for understanding the evolution of polyploid genomes. Since this paleohexaploidy event, the dominant subgenome (LF) has retained 70% of the genes found in *A. thaliana*, whereas 46% and 36% of the genes in MF1 and MF2 have been retained, respectively [43]. Previously, the “two-step theory” explained that the two rounds of independent polyploidization and re-diploidization events resulted in fewer fractionated genes in the newly joined genome, which illustrated the dominant subgenome in *B. rapa* [16]. Additionally, subgenome dominance has been observed in Brassiceae species, suggesting that the dominant subgenome was formed before speciation [43, 55, 56, 58].

In our analysis, we separated the evolution of *B. rapa* into two stages. One stage involves the process of the common ancestor of radish and the three *Brassica* species evolving to the common ancestor of *B. rapa* and *B. oleracea*. The other stage involves the process of intra-specific diversification since its divergence from the common ancestor of *B. rapa* and *B. oleracea*. In both stages, we observed lower gene fractionation rates in the LF subgenome than those of the MF subgenomes. As all three subgenomes were co-existing during these two stages, the “two-step theory” cannot be used to explain the difference. The lower gene fractionation rates in LF should be determined by the features of the subgenome themselves.

### The inferred ancestral genome provides a reference for investigating gene fractionation during genome diversification

Brassiceae species evolved from a common tPCK-like ancestor genome before WGT [55] and formed the dominant subgenome after WGT [43]. Based on comparative analysis of genome sequences of Brassiceae species, Cheng et al. constructed the hexaploid ancestor of the tribe Brassiceae [44]. However, we cannot use this hexaploid ancestor to investigate the intraspecific diversification, as we cannot distinguish gene fractionation during speciation from that during intraspecific diversification. To address this, we constructed a *B. rapa* ancestral genome based on a pan-genome strategy. The inferred ancestral genome provided an essential reference to investigate gene fractionation during individual genome evolution. The biased gene fractionation during intraspecific diversification highlighted that the dominant subgenome was associated with both speciation and intraspecific diversification. Our study demonstrates the importance of the inferred ancestral genome in the investigation of gene fractionation during intraspecific diversification.

The strategy to construct an inferred ancestral genome by merging and ordering all non-redundant syntenic genes to a known reference genome before polyploidization is universal for all genomes that experienced polyploidization. It can be used either for one species or several species with relatively closer evolutionary relationships. In addition to *B. rapa*, we constructed an ancestral genome for four Brassiceae species by merging the genes of a reference genome for each of them. Although we provided examples of *Brassica* species, the concept of the pan-genomic ancestral genome should be extended to analyze other polyploid species. Moreover, the pan-genomic ancestral genome, being a construct, improves as gene content is added from other Brassiceae species.

### Our pan-genome studies revealed the role of SVs on intraspecific diversification and trait domestication

Recently, SVs have been reported to regulate gene expression and influence important traits such as flavor, fruit size, and flowering time [28, 32]. The reason SVs may have such regulatory roles is that SV can bring transcriptional units, evolved to fit one environment, under the control of cis regulatory sequences that have evolved to fit an entirely different environment. The result may be an unexpected, unevolved regulatory changes where some of these changes contributed to fitness. Since polyploidy creates entire genomes that are under relaxed purifying selection, polyploidy may provide sub-genomic environments that promote “evolvability.”

The pan-genome, constructed from individual de novo assemblies, can resolve the vast majority of SVs and further help explore the impacts of SVs on genome and phenotype diversification. Our methods combine pan-genome and large-scale resequencing to investigate SV landscapes in a large population and the possible influence of SV on morphological domestication. Based on the representative genome sequences, we obtained a comprehensive and non-redundant SV set. Then, we constructed an integrated graph-based genome and genotyped all SVs in 524 genomes. We observed that SVs were associated with *B. rapa* morphotype domestication. Previously, domestication and GWAS (Genome-Wide Association Studies) analysis were limited to small variants (SNPs and InDels), leaving the impacts of the vast majority of SVs largely hidden. Pan-genome and graph-based genome strategies have thus established a means for deciphering the impacts of SVs on favorable trait domestication. This method was also applied to the soybean pan-genome recently [27]. However, there are still some issues that need to be resolved. For example, we identified three large inversions (size > 1 Mb); however, we were unable to further investigate these in 524 genomes, as such large SVs could not be accurately genotyped by short reads.

### Studies of leafy head domestication and future directions

The leafy head is an important economic trait in *B. rapa* and is the most outstanding feature of Chinese cabbage. The formation of the leafy head consists of a series of complex developmental processes, and it was reported that genes involved in phytohormone and patterning the adaxial–abaxial axes are involved in leaf-heading formation [24, 63–66]. To date, studies have shown that the formation of the leafy head is regulated by many quantitative trait loci (QTLs) with likely small effects [52, 67, 68]. Therefore, there are still many genes unexplored for illustrating the complex mechanism of leafy head formation. From our previous studies, we found six candidate genes involved in auxin and leaf adaxial–abaxial patterning to be related to leafy head formation. This study identified four additional genes that might be involved in leafy head formation. Although, as we explained, these four domesticated genes are excellent candidates to have contributed to leafy head formation, we still have no direct experimental evidence to support this. In the future, we will focus on the functions of these genes and try to decipher the complex leafy head trait.

## Conclusions

In this study, we constructed a *B. rapa* pan-genome consisting of 18 representative accessions and an integrated graph-based *B. rapa* genome. We established core, dispensable, and private genes, which will facilitate the discovery of loci associated with *B. rapa* morphotype domestication. The pan-genome and genotyped variants in 524 diverse genomes serve as a valuable resource for the *B. rapa* research community. We observed high gene variability and enormous structural complexity in the pan-genome. We also found that the gene flexibility during intraspecific diversification was associated with individual genome adaptation. That is, the subgenomes became more different in gene content and rate of accumulation of genomic variants during intraspecific diversification. Additionally, we observed that SV tracks morphotype domestication, and four SV-related genes under extremely strong selection might be involved in the domestication of the *B. rapa* leafy head.

## Methods

### Plant materials

Sixteen *B. rapa* accessions of different morphotypes named BRO, CCA, CCB, CXA, CXB, MIZ, OIA, OIB, OIC, PCA, PCB, TCA, TUA, TUE, TBA, and WTC were used in this study (Additional file 3: Table S1). All 16 accessions were collected from previously reported 199 *B. rapa* accessions [24], including heading Chinese Cabbage, turnips (Chinese and European turnips), sarsons (sarson, rapid cycling, and oilseed), pak choi (pak choi, wutacai, and caixin), and Japanese morphotype (mizuna). We also collected and resequenced 144 *B. rapa* accessions representing different morphotypes in the present study (Additional file 3: Table S15).

### Illumina, PacBio, and Hi-C sequencing

All 16 accessions were planted in a greenhouse during 2018. Genomic DNA was extracted from leaf tissues at 5 weeks of age using a cetyltrimethylammonium bromide (CTAB) method [69], following which the genomic DNA was used for Illumina and PacBio library construction and sequencing. Libraries with an insert size of 20 kb for SMRT PacBio genome sequencing were constructed as previously reported [70], and these PacBio libraries were sequenced on the PacBio Sequel platform (Pacific Biosciences). Libraries for Illumina paired-end genome sequencing were built according to the standard manufacturer’s protocol (Illumina). Illumina reads for the 16 accessions were generated from three paired-end sequencing libraries with insertion sizes of approximately 350 bp, and the libraries were sequenced on an Illumina platform with a paired-end sequencing strategy. The 144 resequencing accessions were subjected to the same methods for extraction of genomic DNA and were sequenced on a BGISEQ-500 platform. The Hi-C libraries of all accessions were constructed following the pipelines described in a previous study [71], and the resulting libraries were sequenced by an Illumina HiSeq 4000 sequencing platform.

### Contig assembly and pseudo-chromosome construction

A hybrid strategy was used to complete the assembly. An average of approximately 12 Gb (~25×) PacBio SMRT reads and 43 Gb (~90×) Illumina reads for each accession were used for draft genome assembly with MaSuRCA (version 3.2.6) [72] by default parameters. Then, BUSCO [73] was used to perform a preliminary assessment of the assembly integrity. Pseudo-chromosomes of 12 accessions with relatively higher contig N50 values were constructed with Hi-C data using the 3D-DNA pipeline (version 180419) [50]. First, we aligned Hi-C reads to hybrid-assembled contigs by Juicer (version 1.6.2) [74]. Second, we used our developed Hi-C misjoins correction pipeline (https://github.com/caixu0518/MisjoinDetect) to detect misjoins and determine breakpoints in the hybrid assembled contigs. Third, we realigned Hi-C data to the corrected contigs using 3D-DNA [50] (parameters: -m haploid -e), Fourth, we used the Juicebox Assembly Tools (version 1.9.9) [75] to visualize the results and correct minor errors by hand. Finally, we used nucmer (version 4.0) [76] to align the Hi-C scaffolding results to *B. rapa* reference genome and determine pseudo-chromosome boundaries.

### Gene prediction and functional annotation

Before gene prediction, we conducted a whole-genome TE annotation of each assembly and constructed TE libraries using EDTA pipelines (version 1.8.3) [77]. We then used RepeatMasker (version open-4.0.7) [78] to mask the whole genome sequences with the TE library constructed by EDTA, and gene predictions were based on the masked genomic sequences. For gene prediction, we used a strategy that combined ab initio, homology-based approach and RNA-seq reads to predict genes. First, AUGUSTUS (version v3.3.3) (https://github.com/Gaius-Augustus/Augustus) and GeneMark (version 4) [79] were used for de novo gene prediction. Second, GeneWise (version 2.4.1) [80] with default parameters was used to predict homology-based gene models. Third, genes were predicted with RNA-seq reads using the Trinity (version r2013-02-25) [81] and PASA (version r20130425beta) [82] pipelines. Finally, we used EVidenceModeler [83] to combine gene models detected by the three steps. After gene predictions, we used InterProScan (version 5.30-69.0) [84] to conduct functional annotation of the 16 gene sets, and information of the annotated domains and gene ontology was extracted from the InterProScan results. All gene models and functional annotations are freely available from the BRAD database. OrthoFinder (version 2.3.11) [85] was used to calculate homoeologous gene sets of the 18 genomes and orphan genes in each genome. Additionally, we used TBtools (version 1.055) to conduct GO enrichment analysis [86].

### Transposable element annotation

All transposable elements in each genome were annotated and classified by EDTA pipelines [77]. Intact LTR-RTs were predicted using LTR_Finder (version 1.07) [87] with the parameters “-D 15000 -d 1000 -L 7000 -l 100 -p 20 -C -M 0.9” and then further filtered and classified into Copia-like and Gypsy-like LTR-RT by LTR_retriever (version 1.9) [88]. The insertion time of intact LTR-RTs was extracted from the results of LTR_retriever.

### Phylogenetic analysis

First, single-copy genes between *B. oleracea* and the 18 *B. rapa* were determined by OrthoFinder [85]. In total, 7900 single-copy gene families were detected within the 19 genomes. Second, the coding sequences of the single-copy gene families were aligned using MAFFT (version v7.402) [89], and then, Gblock (v0.91b) [90] was used to extract the conserved sequences among the 19 genomes. Finally, the phylogenetic tree was constructed by RAxML (version v8.2.12) [91] with 100 bootstrap replicates. The neighbor-joining tree of the *B. rapa* population was constructed as described in Cheng et al. [24].

### Analysis of SNPs and InDels

We used the Nucmer program [76] to align the 17 assemblies to the *B. rapa* reference genome (Chiifu) using the parameters “--mum -g 1000 -c 90 -l 40,’ following which we used the delta-filter with parameters settings “-1” to obtain one-to-one blocks in the alignment results. Finally, the SNPs and InDels in the one-to-one block were extracted using show-snp with parameter settings “–Clr TH.” Furthermore, we used snpEff (version SnpEff 4.3t) [92] software to annotate the effects of SNPs and InDels. In addition, SNPs and InDels were also detected based on the resequencing reads. First, we used fastp (version 0.12.3) [93] with parameters “-z 4 -q 20 -u 30 -n 5” to filter the raw reads. Then, all of the clean reads were mapped to the Chiifu genome (v3) using BWA-MEM (version 0.7.5a-r405) [94] with the default parameters. Next, SAMtools (version 0.1.19-44428cd) [95] was used to convert Sam files to Bam files and filter the PCR duplicates of the reads. Based on the Bam files, variants were call using SAMtools. Finally, we used Perl scripts to select polymorphic loci covered by ≥3 reads and merged all SNPs from 524 accession. Additionally, based on the Bam files, we used Graphtyper (version 2.5.1) [96] to call InDels using the default parameters.

### Identification of structural variants and structural variation genotyping

Genomic structural variants in the *B. rapa* pan-genome were identified using Chiifu as the reference, and each of the other 17 assemblies was aligned to the reference genome to call insertions, and deletions using the smartie-sv pipeline (https://github.com/zeeev/smartie-sv) [97]. To construct a non-redundant structural variation set, we used svimmer (https://github.com/DecodeGenetics/svimmer) to merge similar structural variants from multiple single sample VCF files. Then, we used the Chiifu reference genome and the nonredundant SV set to construct a graph-based genome with the vg pipeline [98]. We mapped the resequencing reads onto the graph-based genome using a vg toolkit with default parameters and genotyped SVs in the 524 genomes. Meanwhile, we used SyRI (version v1.2) (https://github.com/schneebergerlab/syri) [99] to identify genomic translocations and inversions between each of the other genomes and Chiifu. In addition, we identified genes with large effect mutations using the same method as described in Sun et al. [52]. We extracted each genic sequence (genic regions include 2 kb upstream and downstream regions of the gene body) in the reference genome and mapped these sequences onto each of the other 17 genomes using the “mem” algorithm of the Burrows-Wheeler aligner (BWA; version 0.7.5a-r405) [94].

### Transcriptome sequencing and analysis

The roots, stems, leaves, flowers, and seed pods of the *B. rapa* pan-genomic accessions were collected and used for transcriptome sequencing. These data were not only used to predict gene models, but also to calculate gene expression levels in each genome. All raw reads were filtered by fastp [93] using the parameter “-z 4 -q 20 -u 30 -n 5.” Hisat2 (version 2.2.0) [100] was used to align all clean reads to the corresponding genome, and then, StringTie (version 2.1.3b) [101] was used to calculate the FPKM (fragments per kilobase of exon model per million mapped fragments) value of each gene. Transcriptome data of 86 *B. rapa* accessions (including 44 heading and 42 non-heading accessions) were collected in our previous study [24], and we used the same method to calculate the TPM values (transcripts per million clean tags) of each gene in the Chiifu genome.

### Identification of PAVs

We used “show-diff” in MUMmer [102] to select for unaligned regions of each genome to obtain potential PAV sequences of the 17 genomes relative to the reference genome, and we filtered the unaligned sequences in gap regions and sequences with the feature type “BRK.” Then, we mapped these unaligned sequences to the reference genome with the parameter settings “-x asm10” using minimap2 (version 2.14) [103], and the sequence covering >80% was filtered out to obtain the final PAV region.

### Construction of the *B. rapa* three subgenomes and the *B. rapa* ancestral genome

We used SynOrths [104] to identify syntenic gene pairs between each of 18 genomes and *A. thaliana*. Then, the least fractionated (LF), the medium fractionated (MF1), and the most fractionated (MF2) subgenomes of each accession were built using previously reported methods [16]. Based on the subgenome infroamtion, we calculated single-, two-, and three-copy genes in *B. rapa* (Additional file 2: Figure S34)*.* And a 500-gene sliding window with an increment of two genes was adopted to calculate gene densities in the three subgenomes.

Using *A. thaliana* as the reference, we generated syntenic gene arrays for *A. thaliana* and the 18 genomes on the three subgenomes. Additionally, we calculated syntenic genes between *A. thaliana* and each of the 18 *B. rapa* genomes. Then, we merged syntenic genes of the 18 genomes and removed redundant syntenic genes (Additional file 2: Figure S35). Finally, these genes were ordered based on the tPCK-like ancestor to construct the *B. rapa* ancestral genome [44].

### Analysis of putative selective sweeps and gene haplotypes

All of the SNPs detected in the 524 genomes were further filtered with MAF ≥ 0.05 and missing rate ≥ 0.1, and 1,526,692 were used to detect putative selective sweeps for leaf heading morphotype. In this study, we used three selection methods, namely Fst [105], ROD [106], and cross-population extended haplotype homozygosity (XP-EHH) [107] to detect putative selective sweeps. The Fst and ROD value of each site was calculated by VCFtools [108] and Perl scripts, and the XP-EHH value was calculated using the rehh R package (https://cran.r-project.org/web/packages/rehh/index.html). All of the SNPs in the gene region were connected to represent the haplotype of the gene, and then, the haplotype of each gene was investigated in 524 accessions. Additionally, to identify the relationship between SV and the target morphotype domestication, we defined an SV related to the target morphotype domestication to have the following two characteristics. First, the allele frequency of an SV in the target morphotype was five times that in the others. Second, the SV could be genotyped in most accessions of the two populations, as missing loci typically confound the results.

## Supplementary Information

**Additional file 1.** Supplementary note.

**Additional file 2.** Figures S1–S35.

**Additional file 3.** Tables S1–S38.

**Additional file 4 **The inferred *B. rapa* ancestral genome.

**Additional file 5.** The inferred A_Brassiceae_ and A_Bra_Bol_ genomes.

**Additional file 6.** Review history.

## Data Availability

The genomic sequencing reads and the RNA sequencing data generated in this study have been deposited in NCBI under the accession number PRJNA730930 [109]. The genome assemblies and gene annotations of the 16 accessions in the present study are also freely available from the Genome Warehouse database (https://bigd.big.ac.cn) [110] under accession number PRJCA001831. All of the raw reads generated in this work have been also deposited in the genome sequence archive (https://bigd.big.ac.cn) under the accession number CRA003187. Genome assemblies and annotations of *B. rapa* accessions have been also deposited in Figshare database [111]. The variation datasets for SNPs, InDels, and SVs used in this work, are available through the BRAD website (http://brassicadb.cn) or upon request.
