# Supplementary material for: Impacts of allopolyploidization and structural variation on intraspecific diversification in Brassica rapa
Source: Genome Biol. 2021 May 31;22:166. doi: 10.1186/s13059-021-02383-2 (PMC8166115; doi:10.1186/s13059-021-02383-2)
Supplement: Supplementary file 1 — Additional file 1. Supplementary note. [file 13059_2021_2383_MOESM1_ESM.docx]

**Supplementary note**

**Assembly evaluation**

To evaluate our *de novo* assemblies, we mapped the Illumina reads of each accession onto the corresponding genomes, an average of 99% Illumina reads could be mapped onto each genome (Additional file 3: Table S5), revealing the high accuracy of our assemblies. We also used BUSCO to evaluate the completeness of the 16 assemblies, and an average of 97.3% embryophyte genes was detected in each of the 16 genomes (Additional file 3: Table S6). Additionally, we compared our assemblies with the reference genome, and the dotplot between Chiifu and each genome showed a high degree of consistency overall (Additional file 2: Fig. S1). We also generated whole-genome Hi-C contact maps for the 12 genomes, which further supported the high reliability of the pseudo-chromosomes (Additional file 2: Fig. S2).

**Identification of genes with large effect mutations and TE-related genes in the pan-genome**

We aligned gene sequences in Chiifu to each of the other 17 genomes and detected gene structural variations between Chiifu and the other 17 genomes. In total, we detected 4644–7802 genes with large effect mutations (start-codon mutation, stop-codon mutation, with 3n ± 1 nt indel in CDS and premature stop codon, etc.) in the other 17 genomes (Additional file 2: Fig. S4 and Additional file 3: Table S14).

Transposable elements (TEs) regulate gene expression levels and are also associated with intraspecific diversification. By calculating the distance between LTR-RT and genes, we found that the insertion of LTR-RTs was biased towards gene flanking regions (Additional file 2: Fig. S6). Additionally, there was a strong correlation between the occurrence of LTR-RTs in the upstream and downstream regions of the gene body (R = 0.99, *P* = 5.9e-14), and the average content of LTR-RTs in the upstream regions was dramatically higher than that in the downstream regions (*P* = 6.5e-06) (Additional file 2: Fig. S7). Furthermore, we observed that genes with LTR-RTs inserted in the upstream regions exhibited significantly lower expression levels (*P* = 9.8e-08, t-test) (Additional file 2: Fig. S8), revealing that the insertion of TEs could regulate their target gene expression levels. To investigate the relationships between TEs and the *B. rapa* pan-genome, we calculated the content of LTR-RTs in genic regions, including 2-kb upstream and downstream flanking regions. Compared with the core genes, the insertions of LTR-RTs were more likely to occur in the dispensable genes, and the content of LTR-RTs in dispensable genes exhibited greater differences (Fig. 1h), indicating that the dynamics of TE insertion in dispensable and private genes accelerated genetic variations during intraspecific diversification.

**Genomic variants in 524 devised *B. rapa* genomes**

We analyzed data from a natural *B. rapa* population containing 524 diverse *B. rapa* accessions. Among them, 199 accessions were collected from our previous work [1], 192 were collected from a reported study [2], and 144 accessions were resequenced in the present study (Additional file 3: Table S15). After removing redundancy, we obtained a total of 3.43 Tb resequencing reads generated from the Illumina platform with an average depth of 13.65× (Additional file 3: Table S16). In total, we detected 3,971,130 SNPs and 1,144,753 InDels with a minor allele frequency (MAF) >= 0.05 using the Chiifu genome (v3) as a reference. Among these variations, 8.00% and 5.90% of SNPs were synonymous and non-synonymous mutations, respectively, and 3.82% of InDels were detected in the CDS region (Additional file 3: Table S17). Then, we used all synonymous SNPs with a missing rate <= 0.05 to construct the neighbor-joining tree of 524 *B. rapa* accessions (Fig. 2b). The results revealed that *B. rapa* was divided into turnip, oil type, pak choi, and Chinese cabbage, which was consistent with the classification described by Cheng et al [1]. Additionally, we compared the two SNP sets detected by mapping-calls of 524 resequencing accessions and assembly-calls of the 17 *de novo* assemblies using Chiifu as the reference. In total, we identified 13,619,734 raw SNPs by assembly-calls and 10,711,073 raw SNPs by mapping-calls, and we observed a strong correlation between the two SNP sets (R = 0.99, *P* < 2.2e-16) (Fig. 2d).

**SVs were associated with intraspecific diversification in *B. rapa***

We observed that the average expression level of SV-related genes was significantly lower than that of the genes without SVs (*P* = 3.6e-12) (Additional file 2: Fig. S12). The rate of SV was significantly higher in the upstream region compared with the downstream region (Additional file 2: Fig. S13). The phylogeny of the SVs was very similar to that of the tree constructed using single-copy genes (Additional file 2: Fig. S14), indicating that different accessions accumulated SVs in the same step as SNPs or other small variations*.* Comparing SVs in the pan-genome, the SV density in the non-syntenic region was nearly five times higher than that in the syntenic region between Chiifu and each of the other 17 genomes (Additional file 2: Fig. S15 and Additional file 3: Table S30). As expected, SVs were biased towards occurring in the genic regions of private and dispensable genes (Additional file 2: Fig. S16) because core genes are generally considered to have indispensable functions.

**Identification of translocations and inversions in the pan-genome**

Frequent translocations and inversions were also detected based on the alignments of 18 assemblies. In total, we detected 736–2479 inter-chromosomal and 399–1352 intra-chromosomal translocations, representing genomic sequences of 2.07–7.70 Mb and 2.11–5.19 Mb, respectively (Additional file 3: Table S19). Inter-chromosomal translocations were more likely to occur than intra-chromosomal translocations. Compared with the Chiifu genome, 5.93–14.47 Mb sequences were identified as inversions in the 17 genomes (Additional file 3: Table S20).

**Candidate genes were associated with leafy head domestication**

As the Chinese cabbage populations were large and possessed diverse heading accessions, we selected Chinese cabbage as a representative to further analyze the impacts of SVs on morphotype domestication. We found that SV-related genes in the leaf-heading morphotype were mainly enriched in biological processes related to response to multicellular organism development, developmental process, abiotic stimulus, and response to light stimulus, with some genes enriched in response to hormone, cellular response to hormone stimulus, and hormone-mediated signaling pathway (Additional file 3: Table S37). We also noticed that some genes were enriched in the term of cellular response to hormone, and genes involved in hormone responsive patterns were likely to be associated with leafy head formation in Chinese cabbage, as phytohormones (cytokinin, auxin, gibberellins, and jasmonic acid) are important for leaf shape and polarity [3-6]. In total, there were 12 genes in these terms, one of which was named *BrMYB95.3* and possessed a strong selection signal. Its orthologs in *A. thaliana* are involved in the response to jasmonic acid stimulus, response to auxin stimulus, response to salicylic acid stimulus, and response to gibberellin stimulus. In addition, we identified three other genes with strong selection signals, named *BrPIN3.3*, *BrFL5.1*, and *BrSAL4.2*, whose orthologs in *A. thaliana* encode auxin efflux carrier family proteins or are involved in regulating auxin. Generally, if the frequency of a certain allele was extremely high in the heading group, it indicated that the allele was more likely to be tightly related to the domestication of the leaf heading group. Therefore, we ranked the 1,064 SVs based on the difference in the number of accessions containing the same allele. Surprisingly, the first two genes were *BrPIN3.3* and *BrMYB95.3*, which indicated that the two genes were under very strong selection during the formation of the *B. rapa* leaf heading group (Additional file 3: Table S32).

Additionally, we conducted domestication analysis between the heading and non-heading groups based on whole-genome SNPs. Using SNP-based domestication analysis, we identified a total of 3284 genes from the 33 XP-EHH, 22 ROD, and 23 Fst outlier regions (Additional file 2: Fig. S29 and Additional file 3: Table S38). Furthermore, we compared candidate genes identified by SNPs with those identified by SVs and found that 34 of the 266 candidate genes detected by SV-based domestication analysis were present in the candidate genes detected by SNP-based domestication analysis.

**Candidate genes of *BrMYB95.3, BrFL5.1*, and *BrSAL4.2***

The second SV-related gene was *BrMYB95.3*. Using the same analysis method for *BrPIN3.3*, the results of the SV-based domestication analysis and haplotype analysis all supported that *BrMYB95.3* was closely related to the domestication of the leaf heading group. We also found that the expression level of *BrMYB95.3* with the SV in the heading group was significantly lower than that of the non-heading group (*P* = 0.0107) (Additional file 2: Fig. S30). By analyzing the corresponding orthologous *AtMYB95* gene in the *A. thaliana* genome, we found that the *AtMYB95* gene was reported to be an R2R3-MYB transcription factor, involved in jasmonic acid metabolic process, response to salicylic acid stimulus, and response to jasmonic acid stimulus. The other two genes, *BrFL5.1* and *BrSAL4.2,* were also under strong selection, as detected by SV-based domestication analysis (Additional file 2: Fig. S31–33 and Additional file 3: Table S32). *FL5* was described as encoding a plant-specific protein with a domain of unknown function (DUF828) and a Pleckstrin-like homology domain, which acts to co-ordinate leaf size with vein density [7]. *SAL4* was described as encoding an SAP-type protein, associated with SIT4-type phosphatase, which is required for proper *PIN* polar localization and root development [8].

**Reference**

1. Cheng F, Sun RF, Hou XL, Zheng HK, Zhang FL, Zhang YY, Liu B, Liang JL, Zhuang M, Liu YX, et al: **Subgenome parallel selection is associated with morphotype diversification and convergent crop domestication in *Brassica rapa* and *Brassica oleracea*.** *Nature Genetics* 2016, **48:**1218-1224.

2. Su TB, Wang WH, Li PR, Zhang B, Li P, Xin XY, Sun HH, Yu YJ, Zhang DS, Zhao XY, et al: **A Genomic Variation Map Provides Insights into the Genetic Basis of Spring Chinese Cabbage (*Brassica rapa* ssp *pekinensis*) Selection.** *Molecular Plant* 2018, **11:**1360-1376.

3. Gao LW, Lyu SW, Tang J, Zhou DY, Bonnema G, Xiao D, Hou XL, Zhang CW: **Genome-wide analysis of auxin transport genes identifies the hormone responsive patterns associated with leafy head formation in Chinese cabbage.** *Scientific Reports* 2017, **7**.

4. Luo XY, Zheng JS, Huang RY, Huang YM, Wang HC, Jiang LR, Fang XJ: **Phytohormones signaling and crosstalk regulating leaf angle in rice.** *Plant Cell Reports* 2016, **35:**2423-2433.

5. Santner A, Estelle M: **Recent advances and emerging trends in plant hormone signalling.** *Nature* 2009, **459:**1071-1078.

6. Stamm P, Kumar PP: **The phytohormone signal network regulating elongation growth during shade avoidance.** *Journal of Experimental Botany* 2010, **61:**2889-2903.

7. Prabhakaran Mariyamma N, Clarke KJ, Yu H, Wilton EE, Van Dyk J, Hou H, Schultz EA: **Members of the Arabidopsis FORKED1-LIKE gene family act to localize PIN1 in developing veins.** *J Exp Bot* 2018, **69:**4773-4790.

8. Dai MQ, Zhang C, Kania U, Chen F, Xue Q, Mccray T, Li G, Qin GJ, Wakeley M, Terzaghi W, et al: **A PP6-Type Phosphatase Holoenzyme Directly Regulates PIN Phosphorylation and Auxin Efflux in Arabidopsis.** *Plant Cell* 2012, **24:**2497-2514.
