# Supplementary material for: Impacts of allopolyploidization and structural variation on intraspecific diversification in Brassica rapa
Source: Genome Biol. 2021 May 31;22:166. doi: 10.1186/s13059-021-02383-2 (PMC8166115; doi:10.1186/s13059-021-02383-2)
Supplement: Supplementary file 2 — Additional file 2. Figures S1–S35. [file 13059_2021_2383_MOESM2_ESM.doc]

**Supplementary Figures**


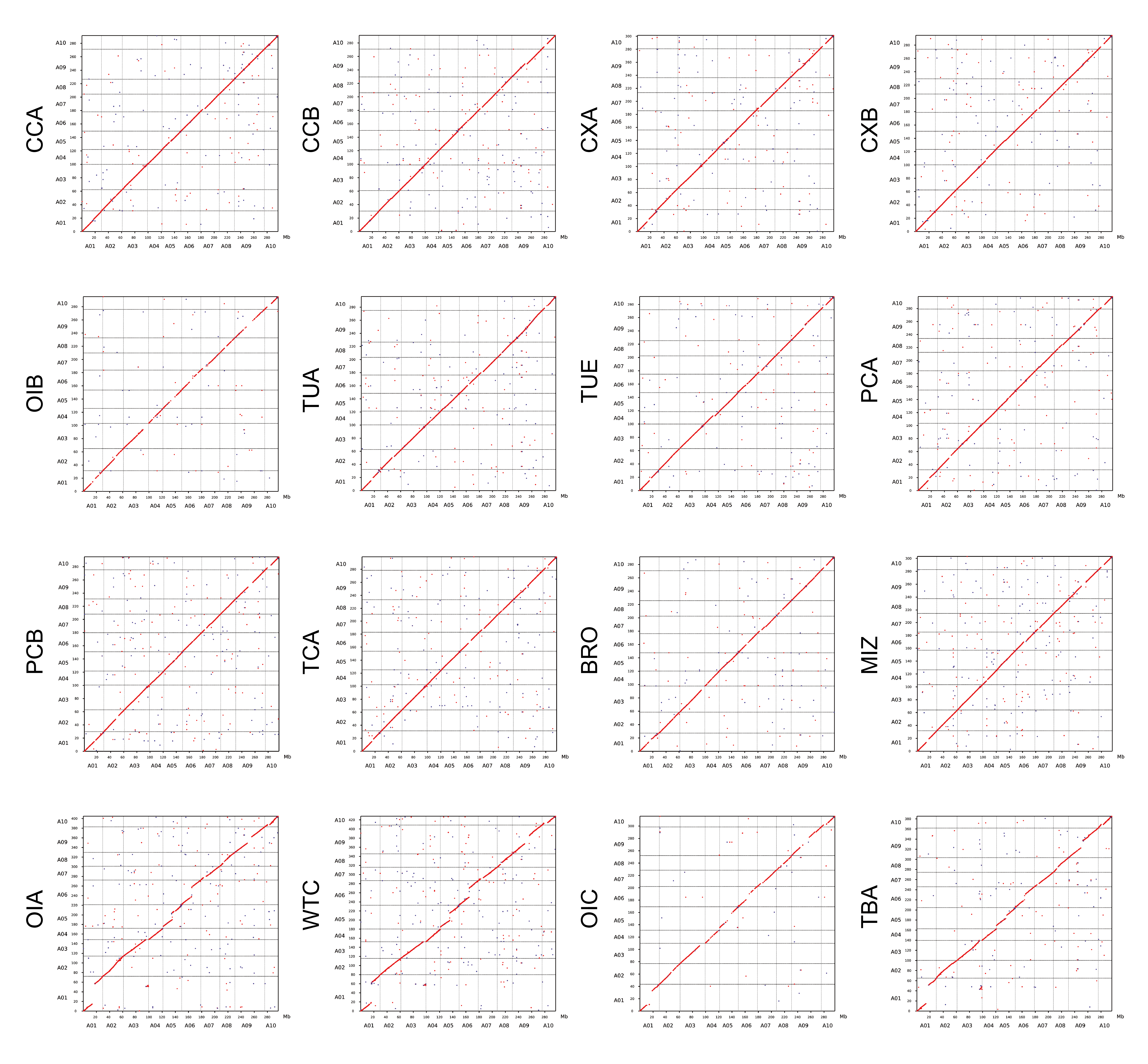


Fig. S1 Dot-plot between Chiifu and each assembly


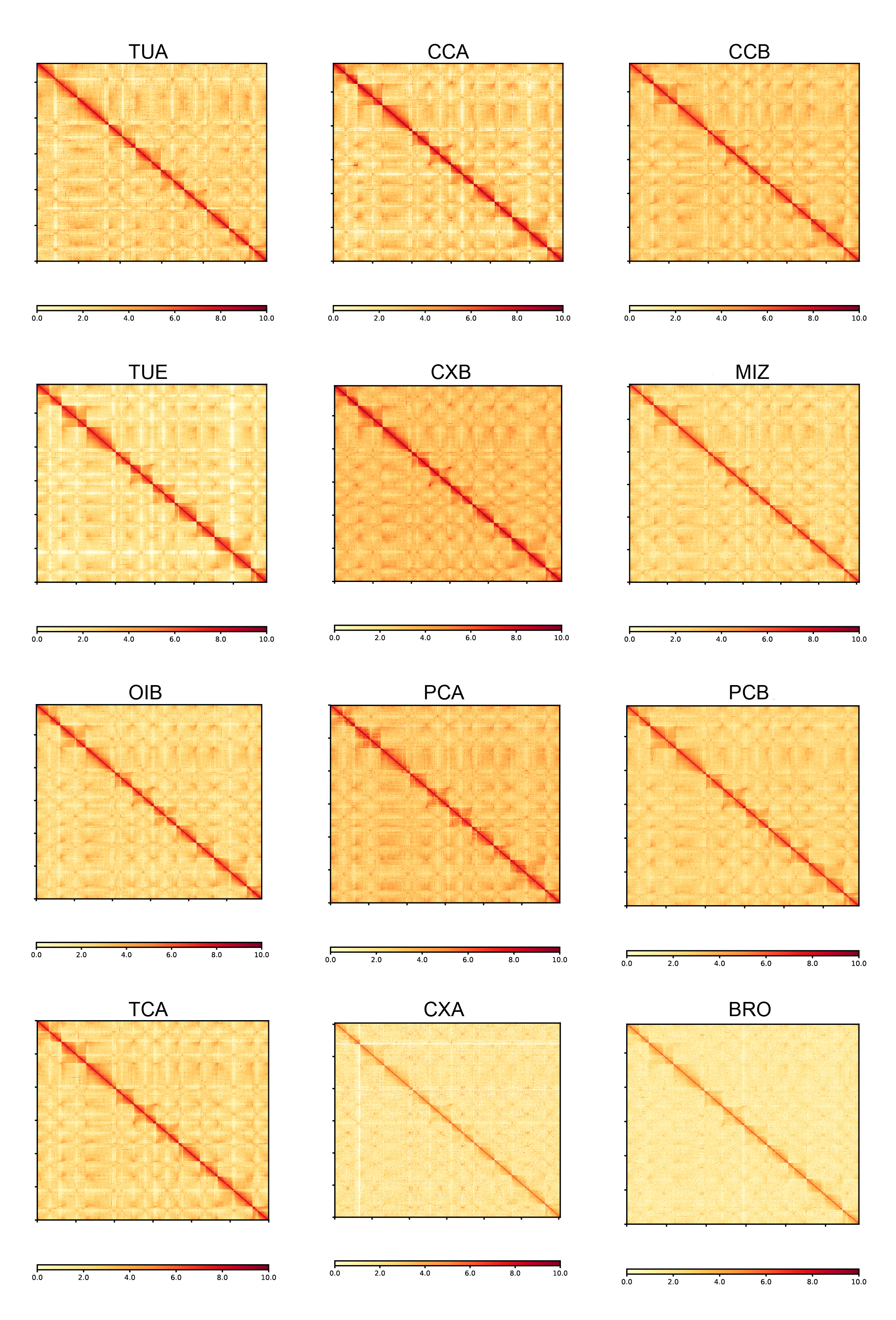


Fig. S2 Hi-C contact maps of 12 *de novo* assembled genomes


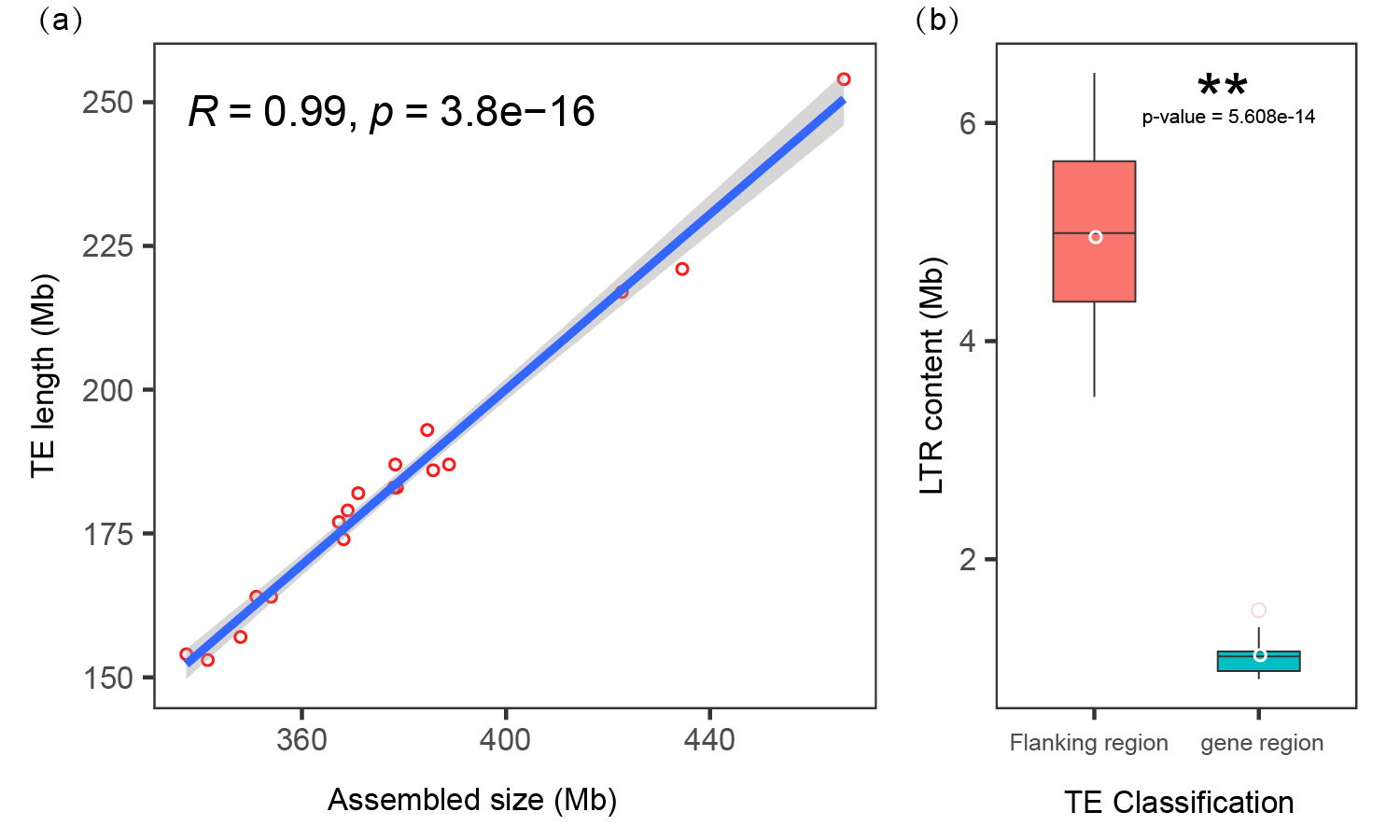


**Fig. S3** The characteristics of TEs in the *B. rapa* pan-genome. **a**, Correlation analysis between TE content and the assembly size of each accession. **b**, Comparison of TEs in the gene flanking region and gene region. The gene flanking region represents the upstream and downstream 2 kb region of the gene body.


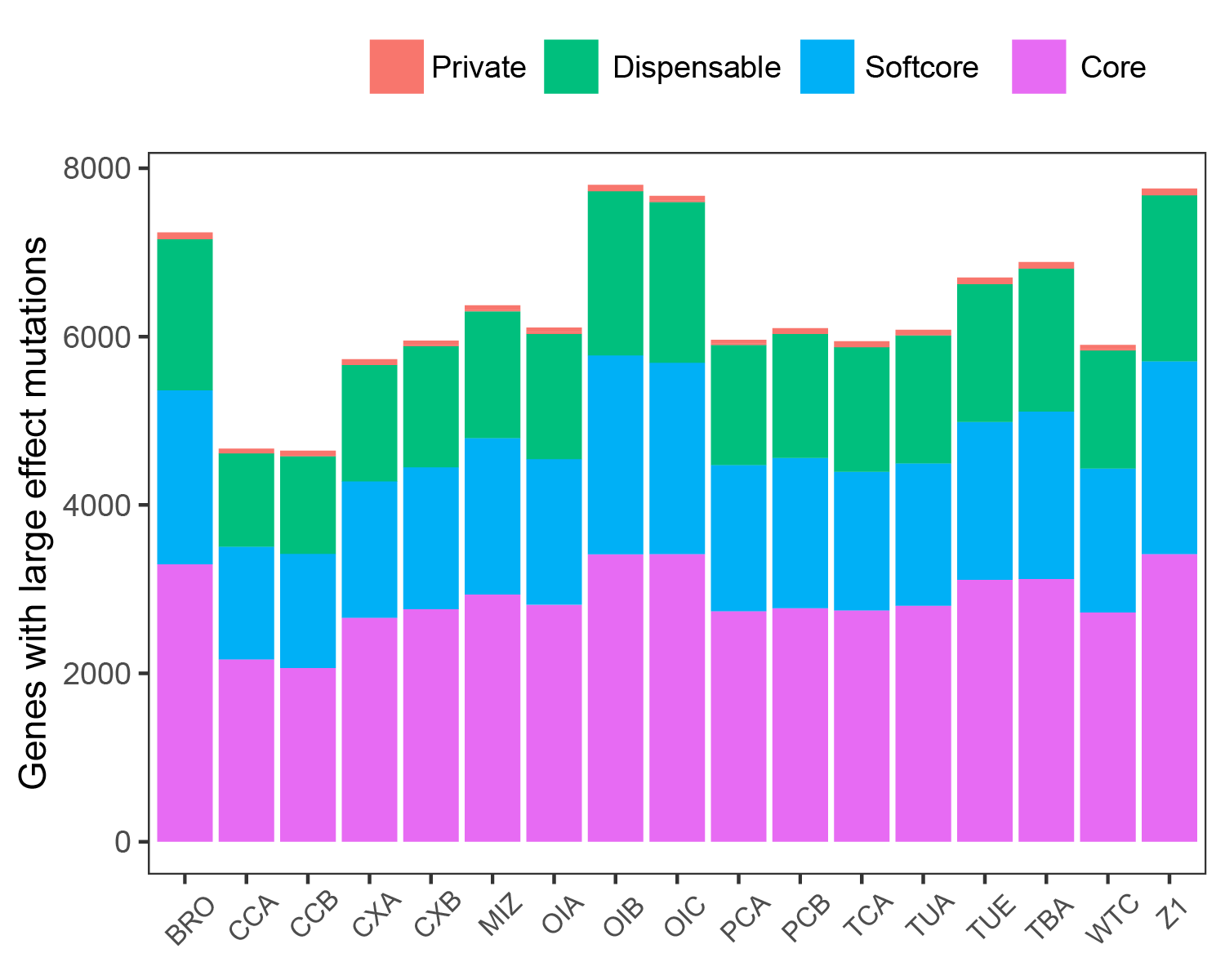


**Fig. S4** Genes with large-effect mutations in the *B. rapa* pan-genome.


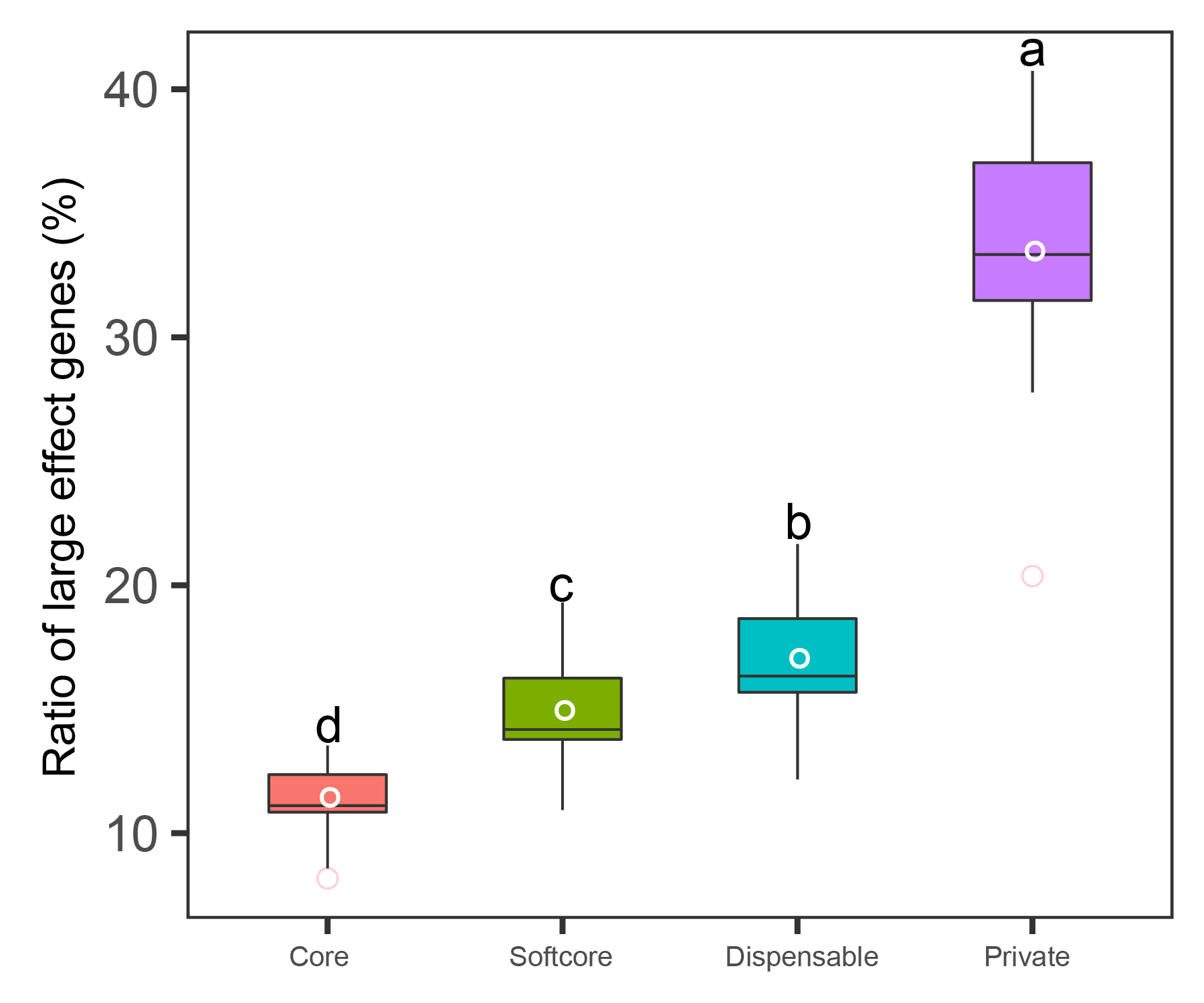


**Fig. S5** The proportion of genes with large effect mutations in the core, softcore, dispensable, and private gene sets. The white dots indicate the average value in the figure. Multiple comparisons are done by the Student-Newman-Keuls test with a = 0.01.


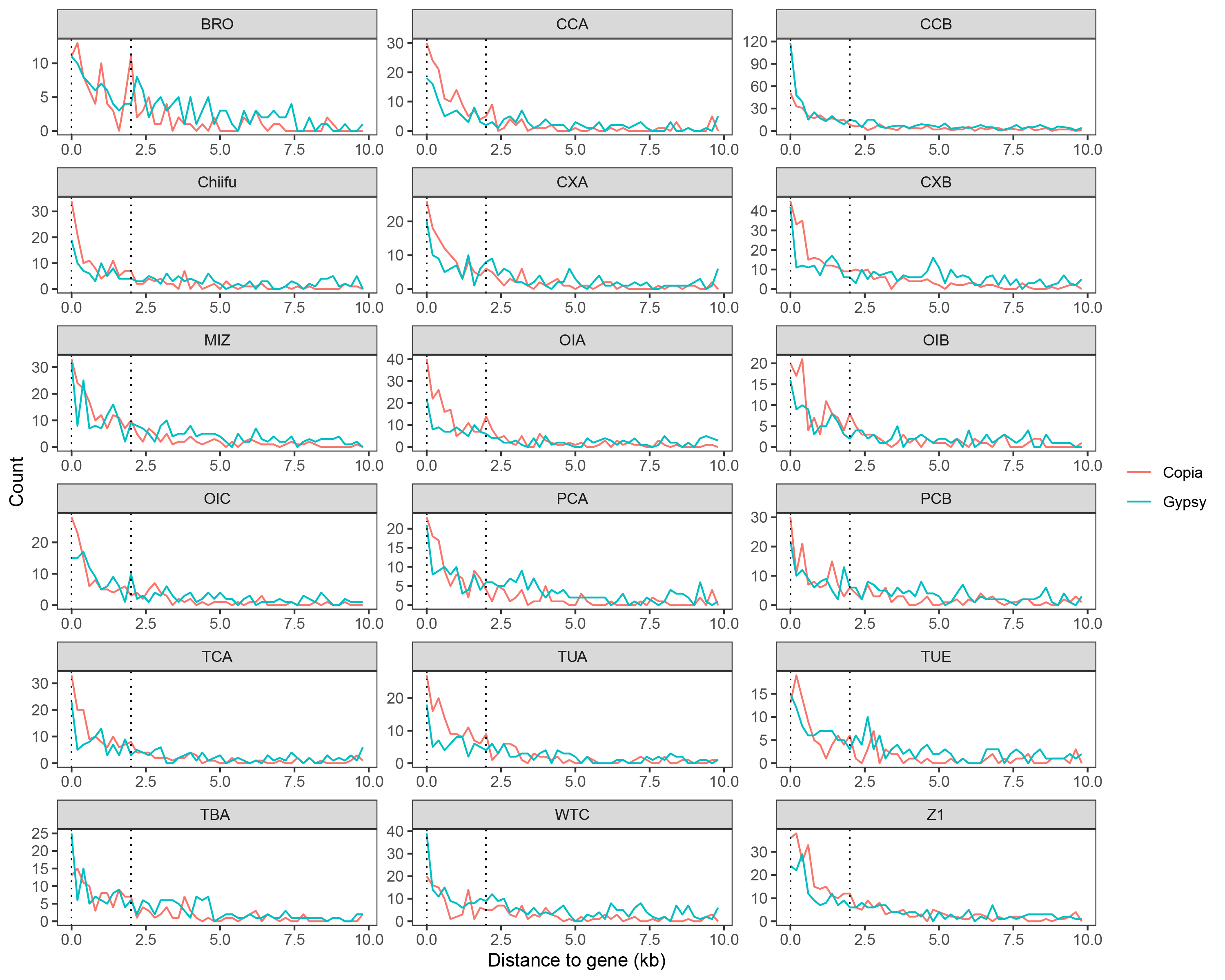


**Fig. S6** The distance between LTR-RT and gene in the eighteen *B. rapa* genomes. The dotted line indicates the position 2kb from the gene body.


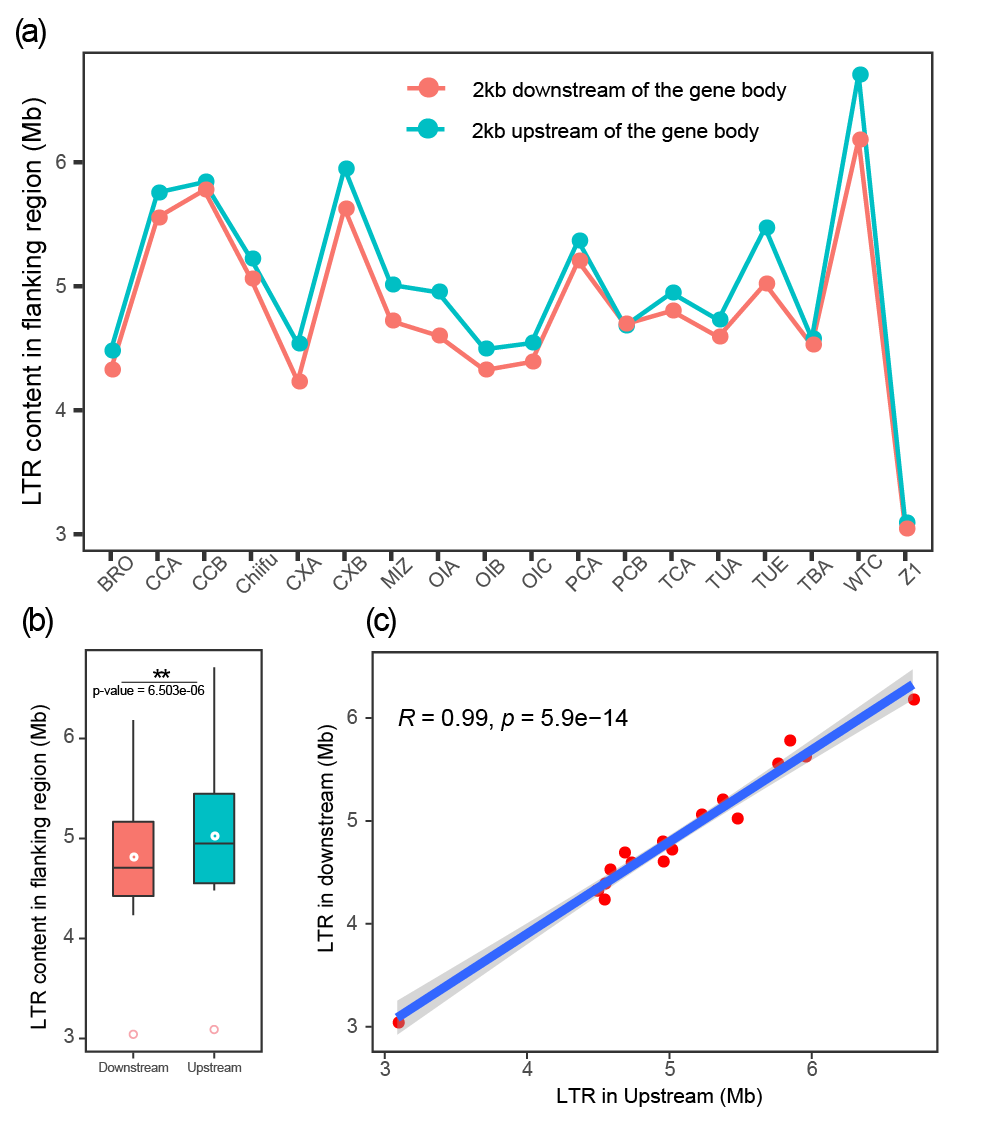


**Fig. S7** The characteristics of LTR-RTs in the gene flanking regions. **a**, LTR-RT content in 2 kb upstream and downstream regions of the gene body. **b**, Comparisons of LTR-RT content in downstream and upstream regions of the gene body. **c**, Correlation analysis of the insertions of LTR-RTs in upstream and downstream regions of the gene body.


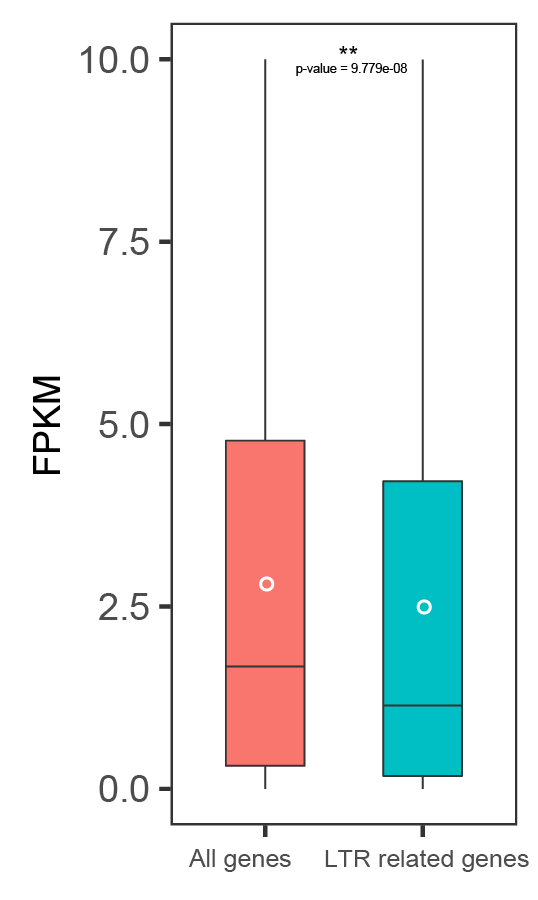


**Fig. S8** Comparison of the expression levels between genes with LTR-RT insertion in upstream regions and gene regions in the Chiifu genome.


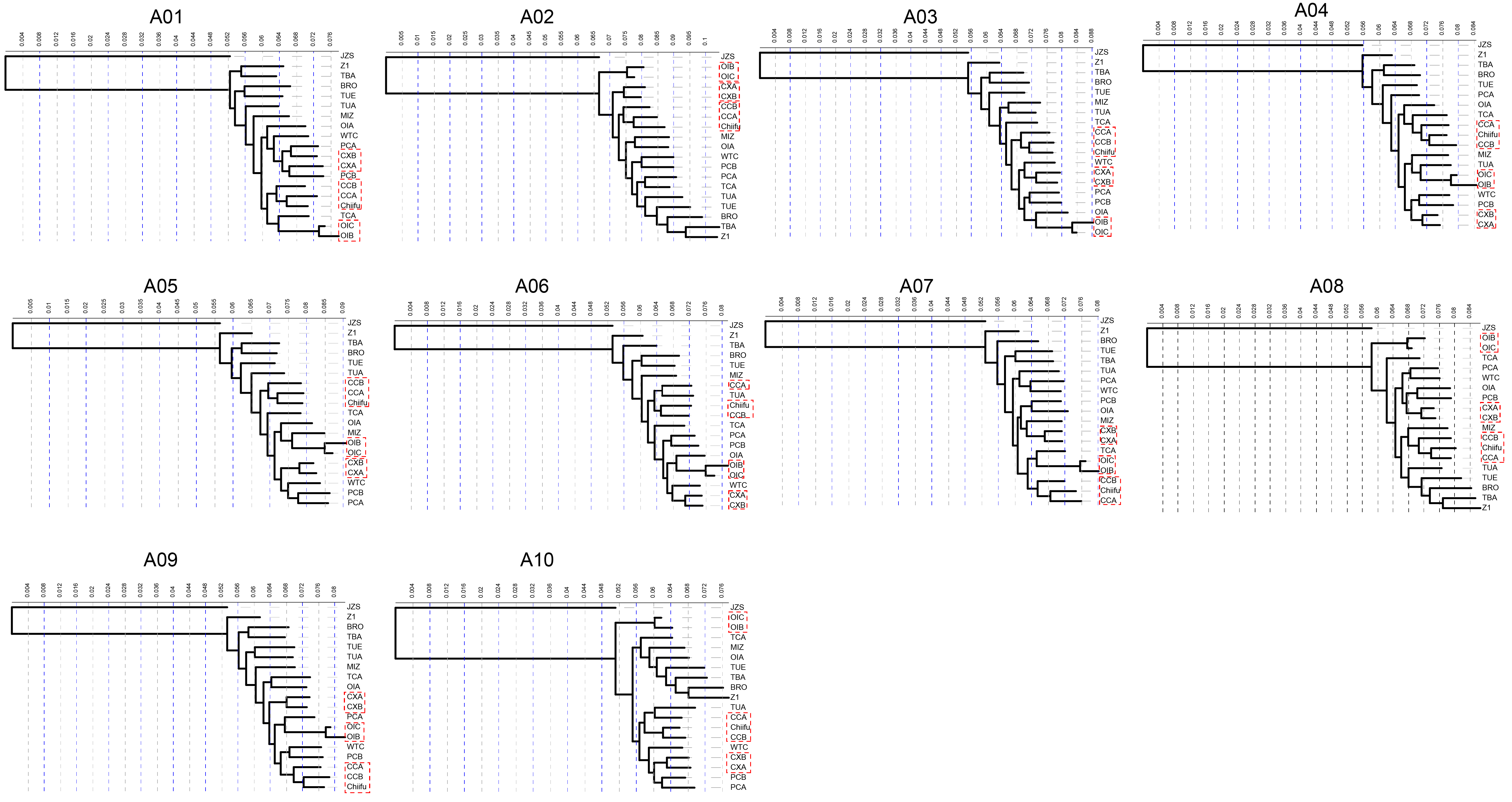


**Fig. S9** Phylogenetic trees of *B. rapa* ten chromosomes. Single-copy genes on each chromosome are used to construct the ten phylogenetic trees. Specific accessions (i.e., OIB and OIC) are marked with red-dotted lines to present extensive phylogeny discordance.


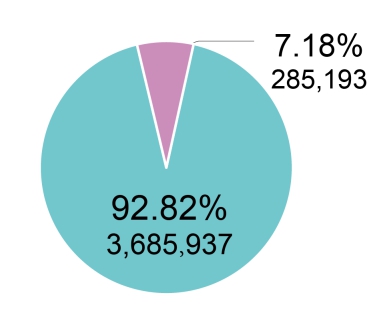


**Fig. S10** Comparisons of SNP data sets detected by mapping-calls of 524 resequencing accessions and assembly-calls of the 17 *de novo* assemblies. The light blue indicates that the SNPs detected by mapping-calls of 524 resequencing accessions are present in the SNP data set detected by assembly-calls of the 17 *de novo* assemblies using Chiifu as the reference, and light purple indicates that SNPs not detected in the SNP data set which is detected by assembly-calls of the 17 *de novo* assemblies.


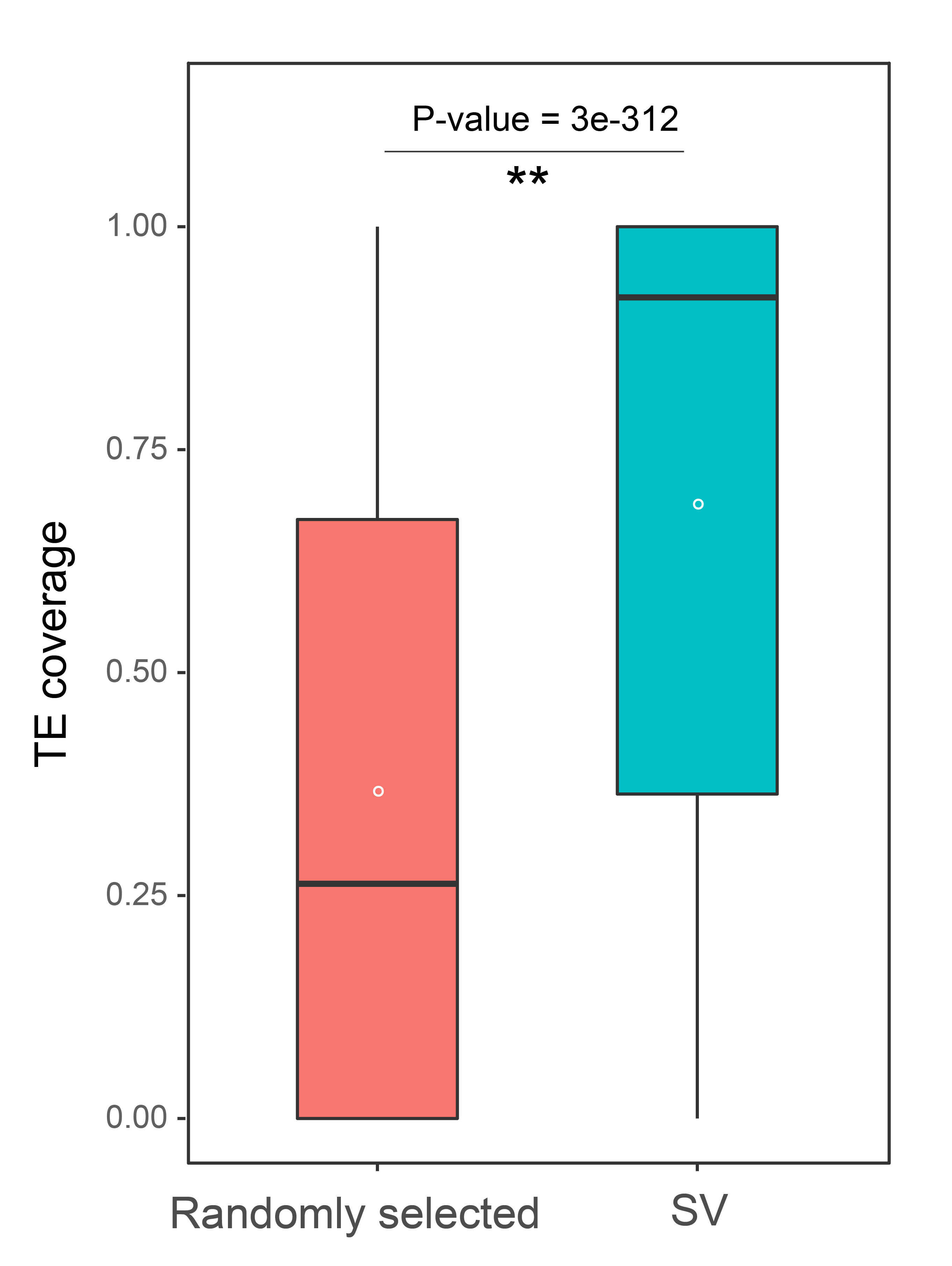


**Fig. S11** Comparison of TE coverage between SV regions and genome regions selected randomly. We randomly selected 4000 SV sequences with a length of 1-2 kb, and also randomly selected 4000 genomic sequences with a length of 1-2 kb from the whole genome, and calculated the TE coverage of the two sets of sequences.


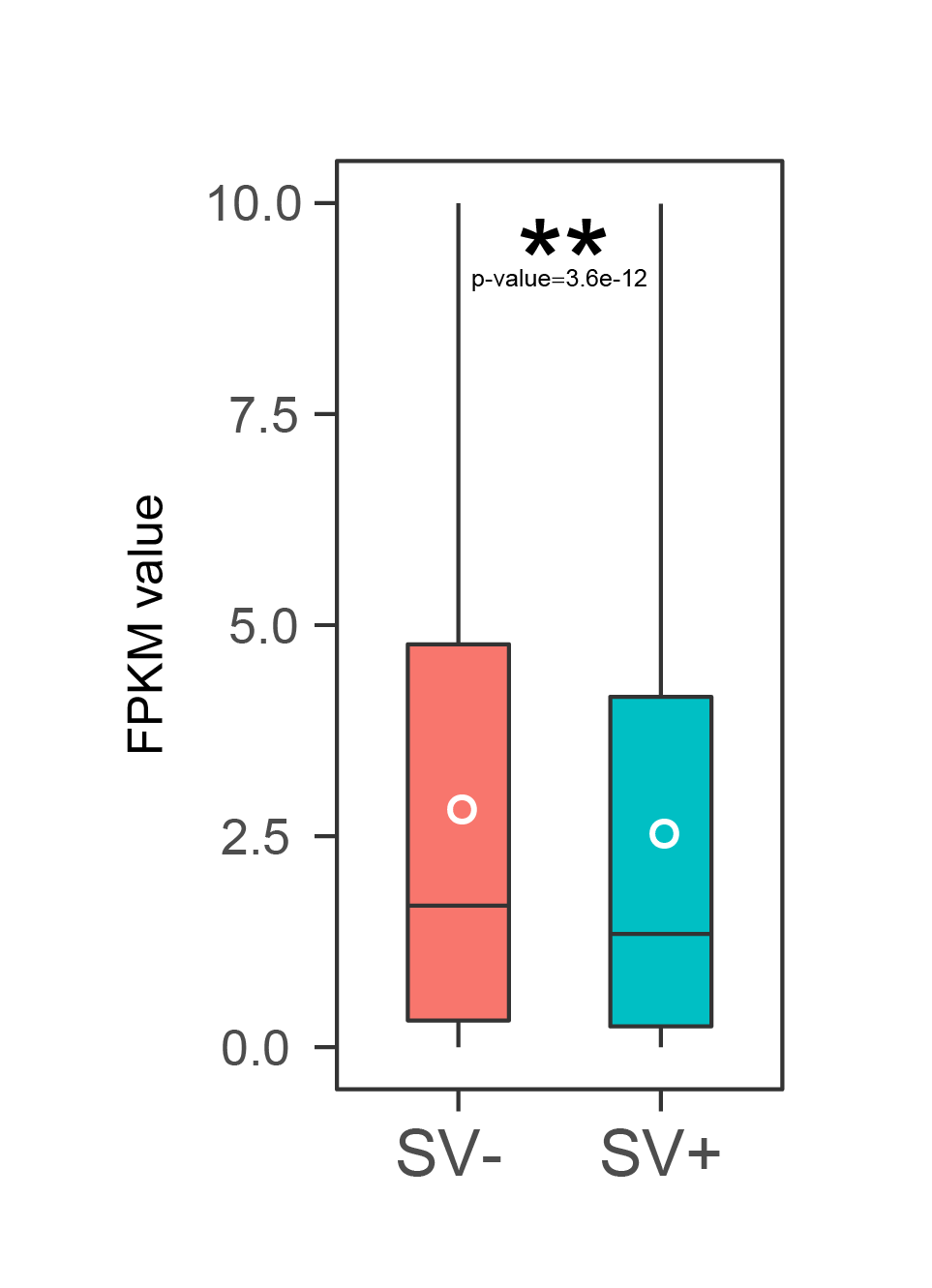


**Fig. S12** Comparison of the expression levels of genes with SVs and without SVs in the Chiifu genome.


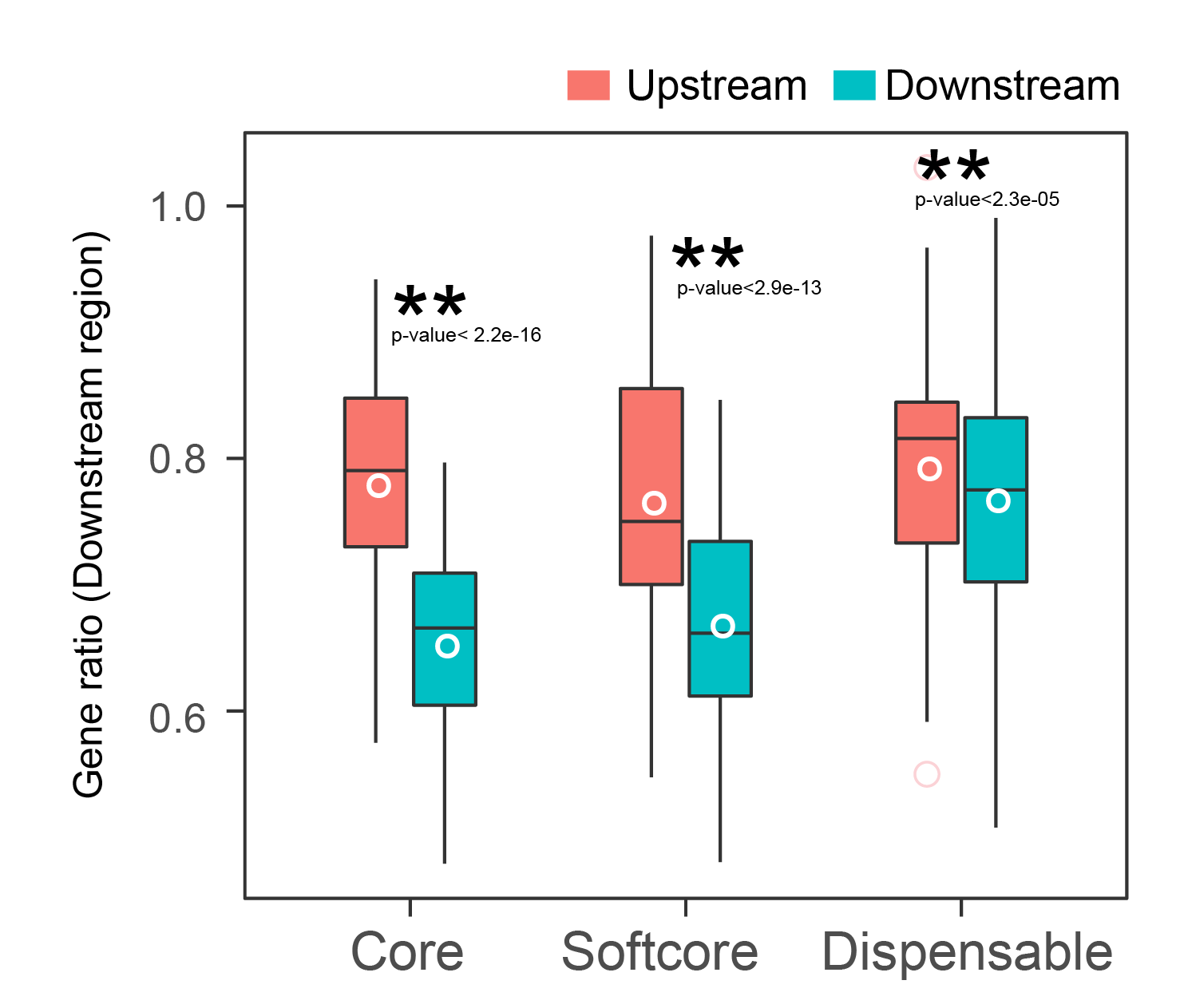


**Fig. S13** The proportion of genes with SVs in upstream (red) and downstream (blue) regions.


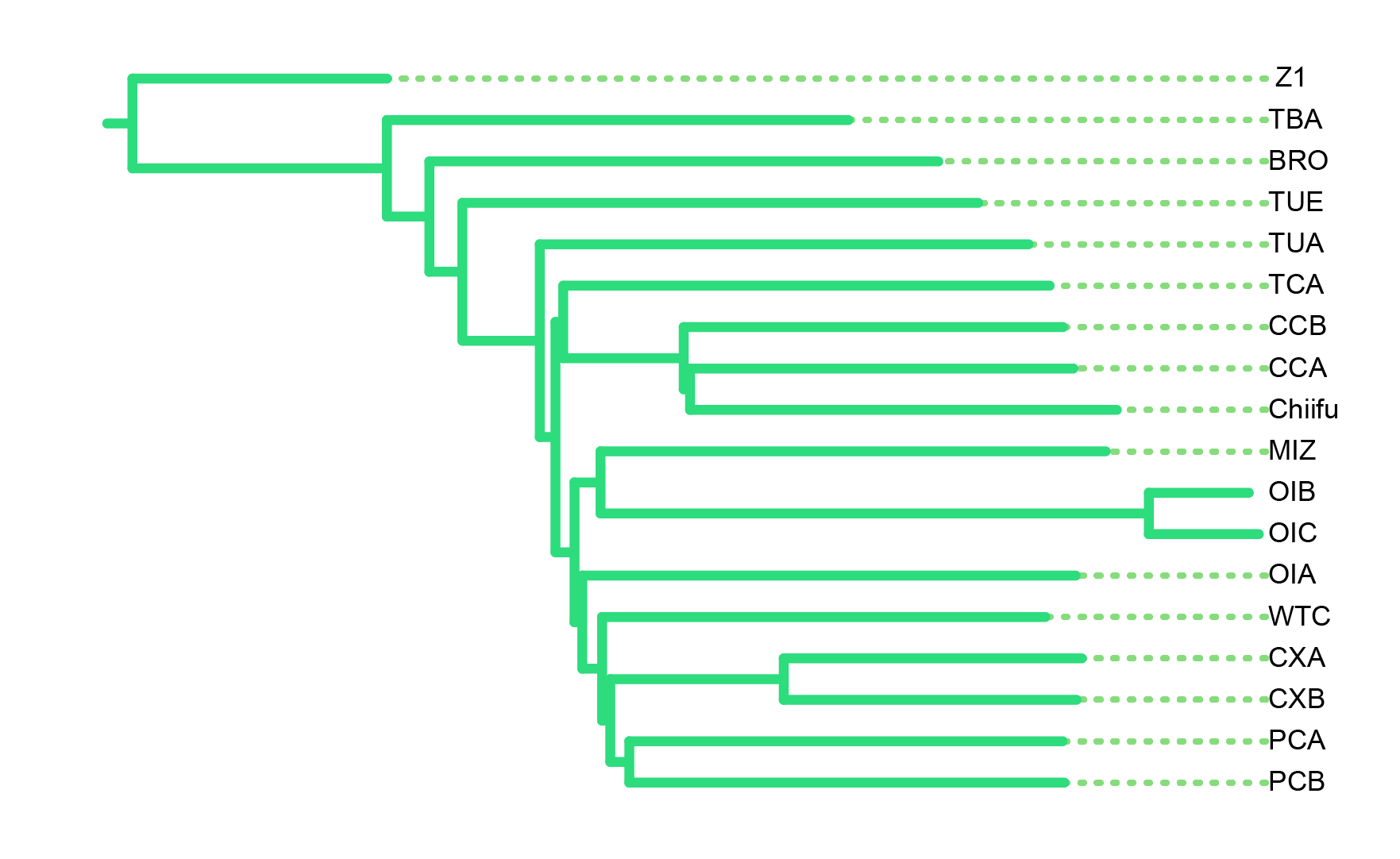


**Fig. S14** A neighbor-joining tree constructed for 18 genomes by whole genome SVs.


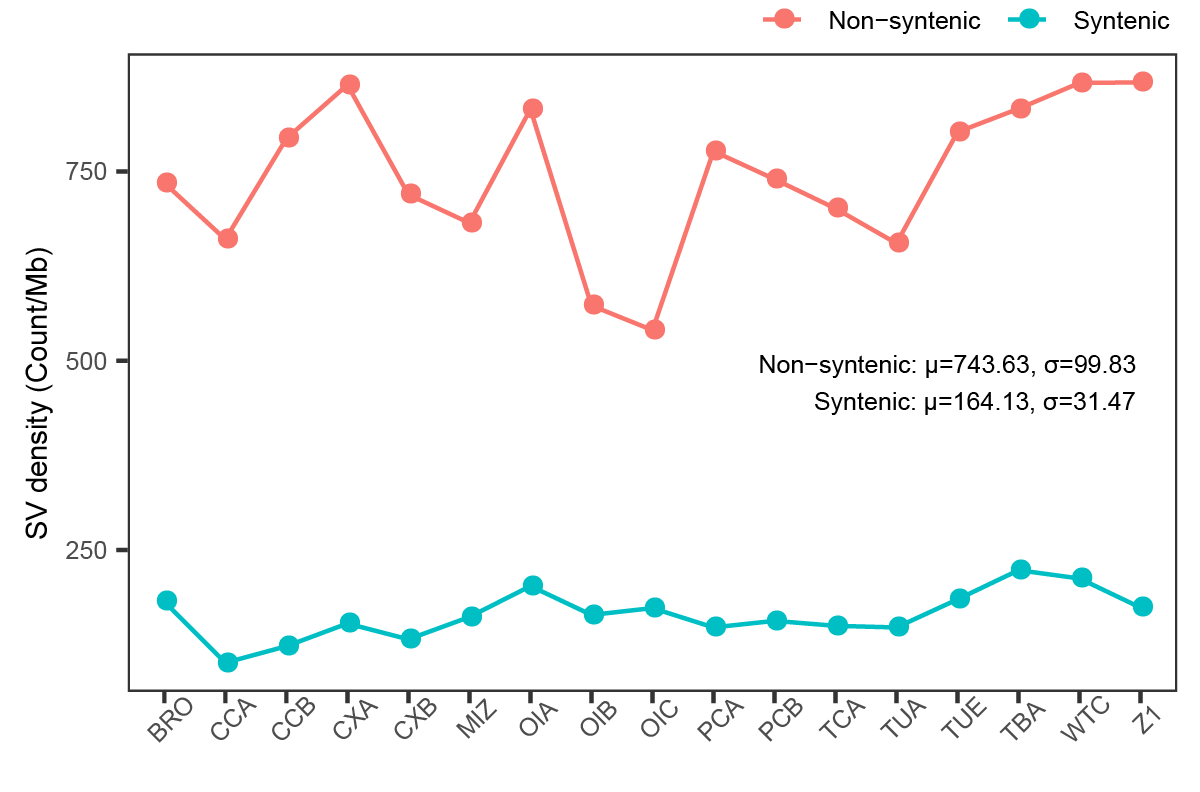


**Fig. S15** SV density in the syntenic and non-syntenic regions.


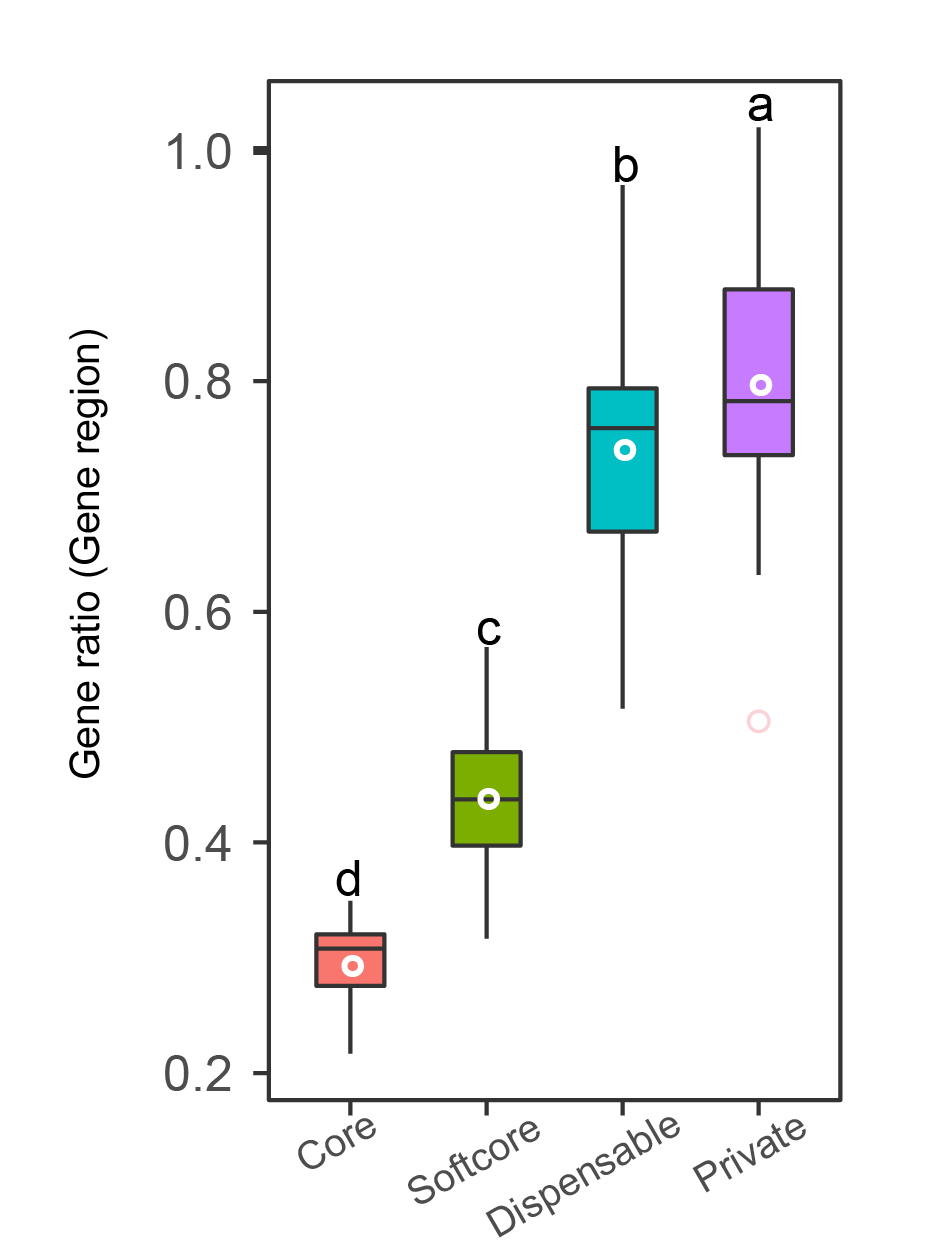


**Fig. S16** The ratio of SV-related genes in the core, softcore, and dispensable genes.


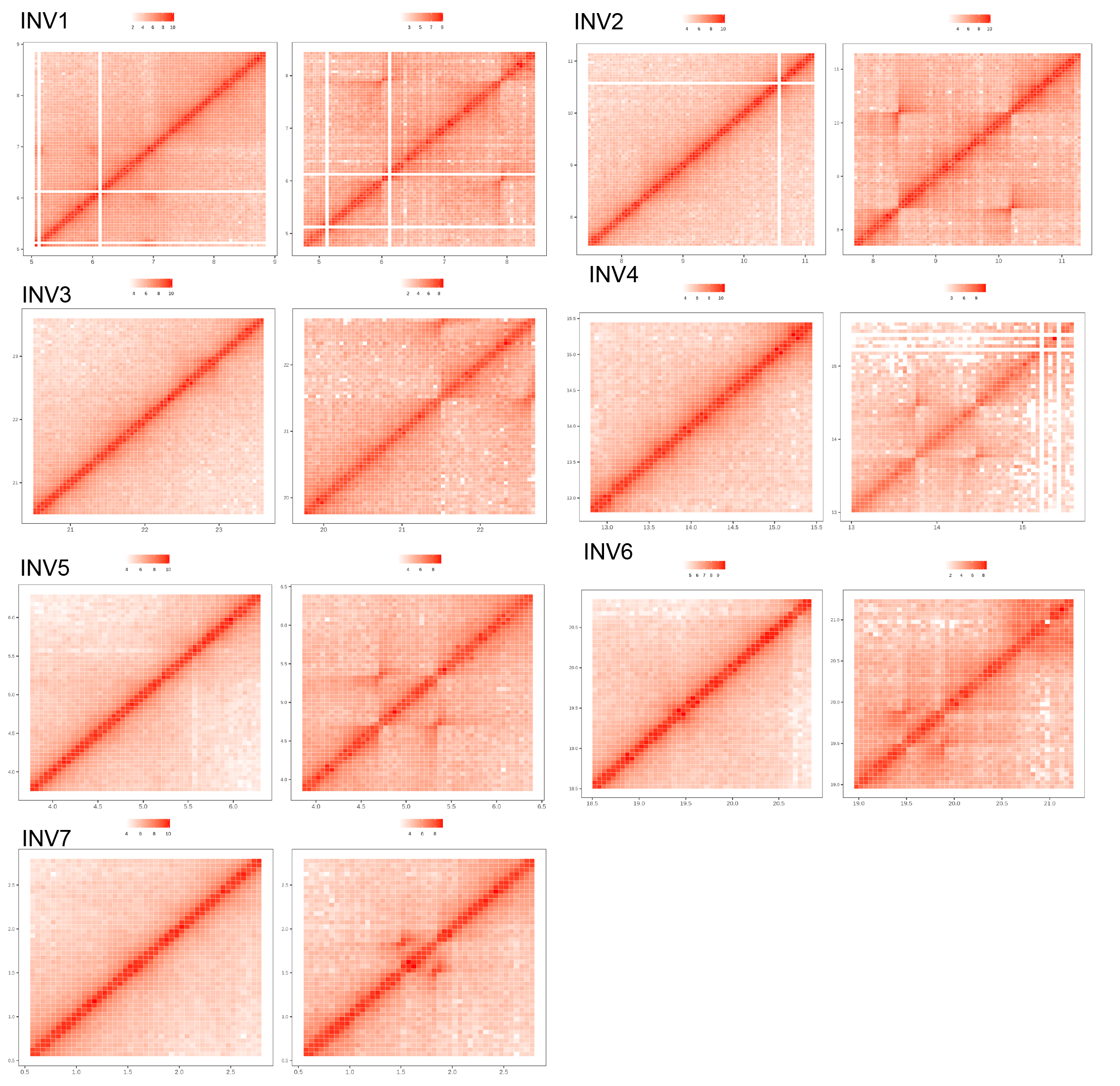


**Fig. S17** Seven large genomic inversions verified by Hi-C reads. The detailed information of each inversion is recorded in Supplementary Table S21. Each inversion is verified by Hi-C data. The Hi-C contact map on the left indicated the signals of Hi-C data of Chiifu to the Chiifu genome sequences. The Hi-C contact map on the right indicated the signals of the Hi-C data of other materials to the corresponding genome fragment of Chiifu. The *x-*axis and *y*-axis represent the position of fragments, and the heatmap represents the normalized Hi-C contact value (log2).


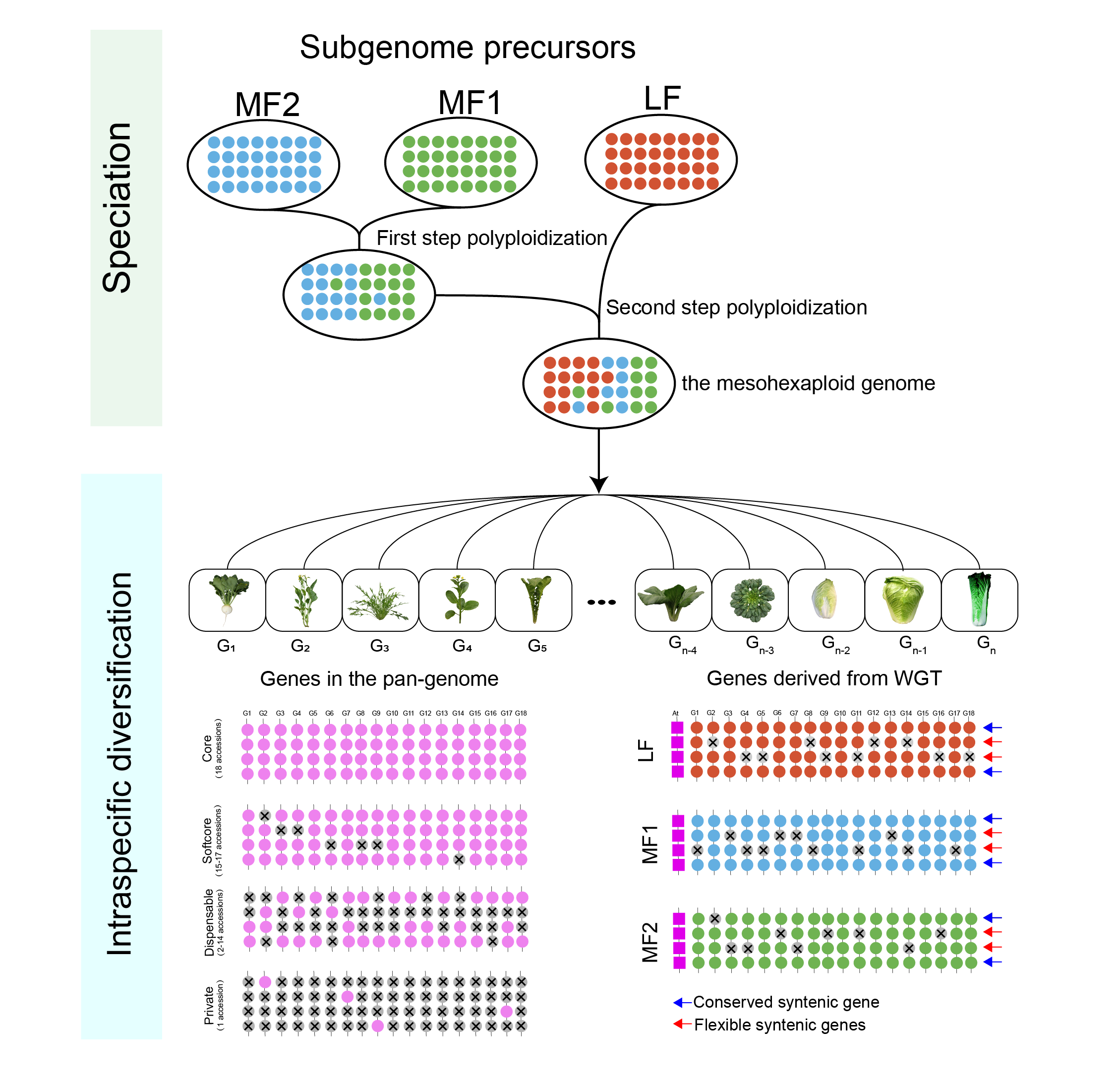


**Fig. S18** The speciation and intraspecific diversification in *B. rapa*. We used figures to present the diversifications of genes in the *B. rapa* pan-genome and genes derived from WGT. The pink squares represent genes in *A. thaliana*, and the red, green, and blue dots represent genes on the LF, MF1, and MF2 subgenomes, respectively. Undetectable gene is indicated by gray dots with a black mark.


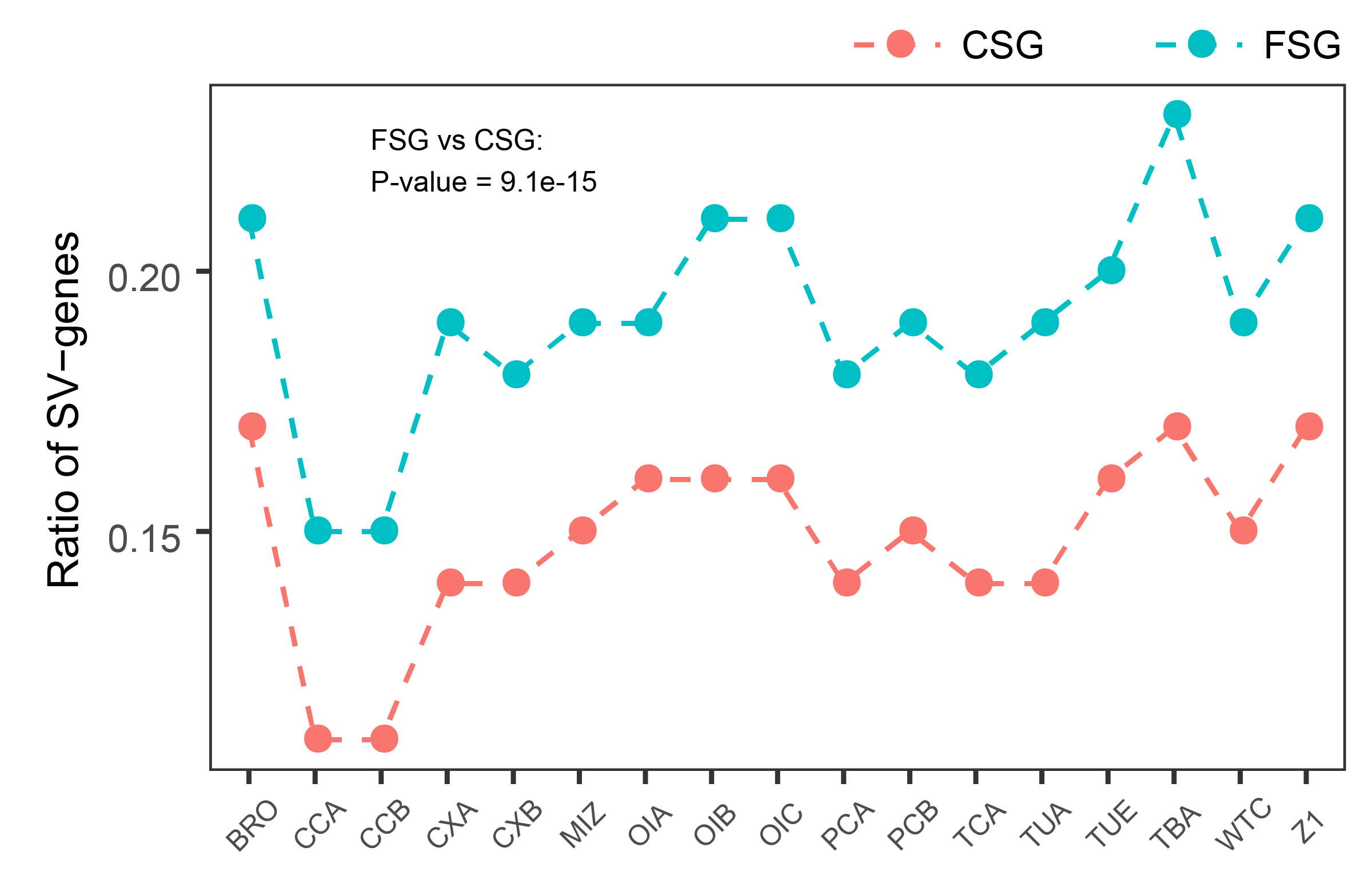


**Fig. S19** Ratio of SV-genes in CSGs and FSGs. SV-gene represents that an SV occurred in the gene region.


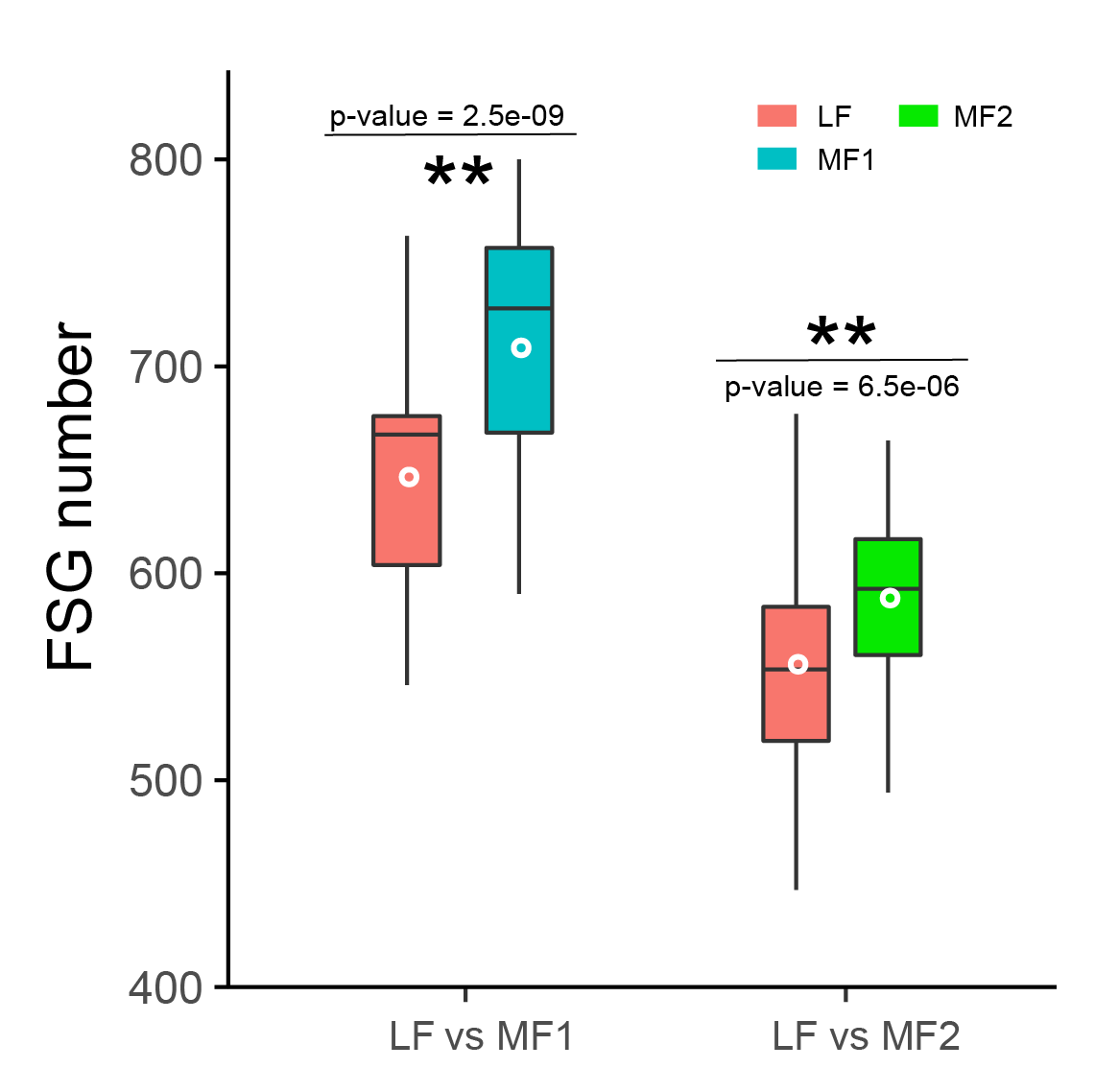


**Fig. S20** Number of FSGs in homoeologous gene pairs between LF and MF subgenomes


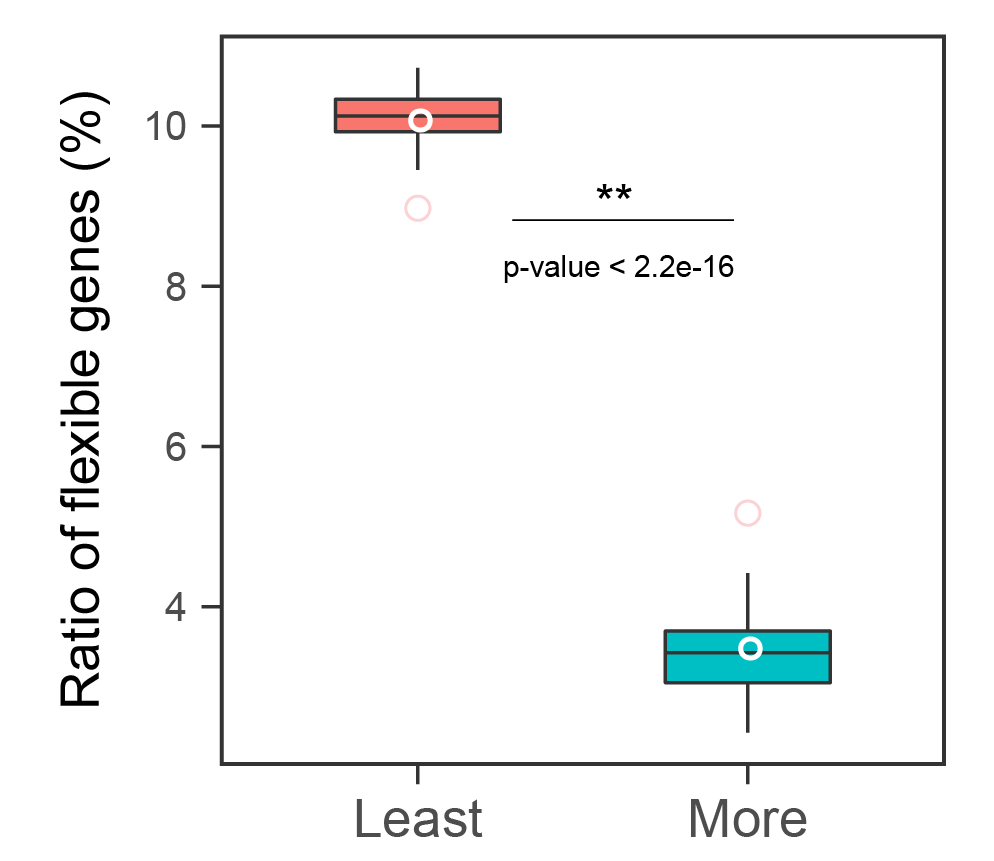


**Fig. S21** The ratio of least and more flexible syntenic genes in the two-copy genes.


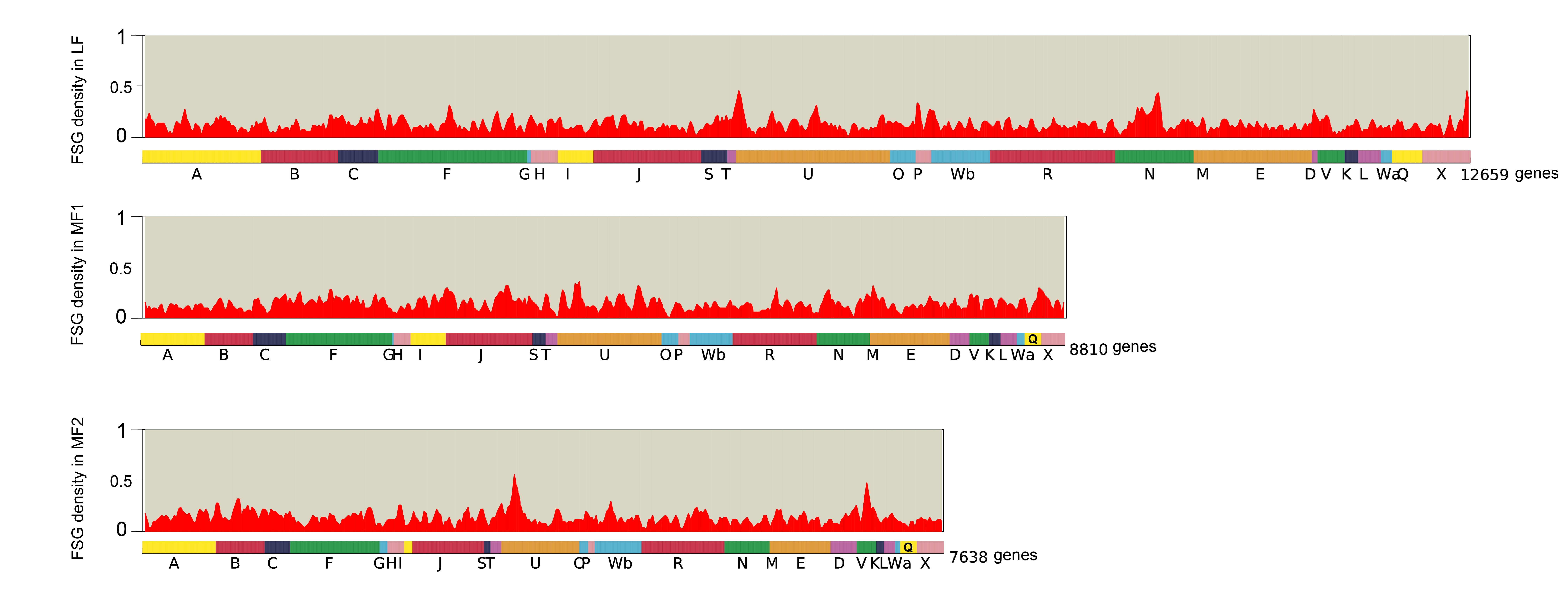


**Fig. S22** Distribution of FSGs across *B. rapa* three subgenomes. A 50 genes sliding window with an increment of 20 genes is adopted to calculate the FSG density in the Chiifu three subgenome. Red indicates the density of FSGs.


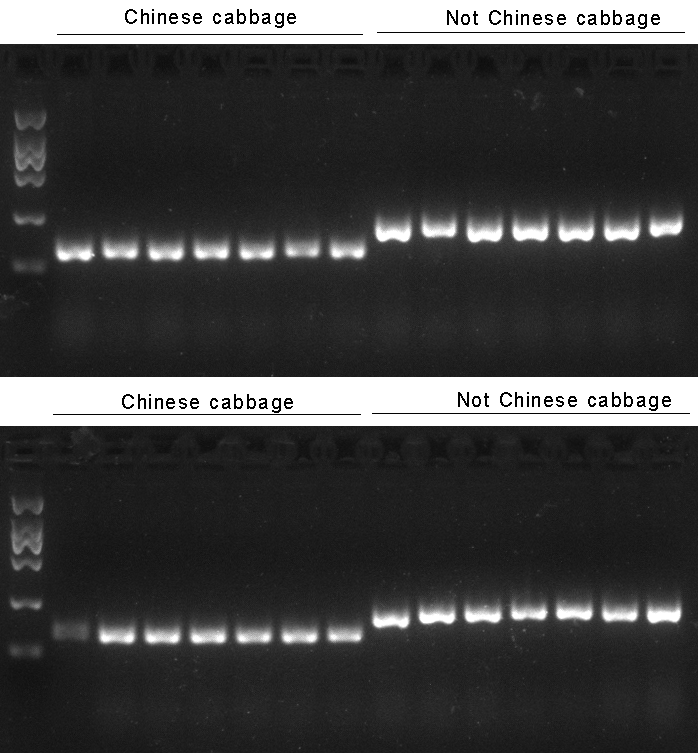


**Fig. S23** Validation of a selected SV between Chinese cabbage and other sub-populations using PCR amplification. A 74-bp deletion occurred in Chinese cabbage, which was verified in 14 Chinese cabbage and 14 non-Chinese cabbage.





**Fig. S24** An example of an SV-related gene is associated with Pak choi domestication. **a**, The distribution of haplotypes in BraA03g021890.3.1C gene region in 524 genomes. **b**, The distribution of one genotype of the SV in BraA03g021890.3.1C region in 524 genomes. **c**, The genotype of SV in the BraA03g021890.3.1C gene region in European turnip group and others. Detailed information about the putative SVs and SV-related genes in the domestication of the Pak choi population is summarized in Table S33.





**Fig. S25** An example of an SV-related gene is associated with European turnip domestication. **a**, The distribution of haplotypes in BraA03g002340.3.1C gene region in 524 genomes. **b**, The distribution of one genotype of the SV in BraA03g002340.3.1C region in 524 genomes. **c**, The genotype of SV in the BraA03g002340.3.1C gene region in European turnip group and others. Detailed information about the putative SVs and SV-related genes in the domestication of the European turnip population is summarized in Table S34.


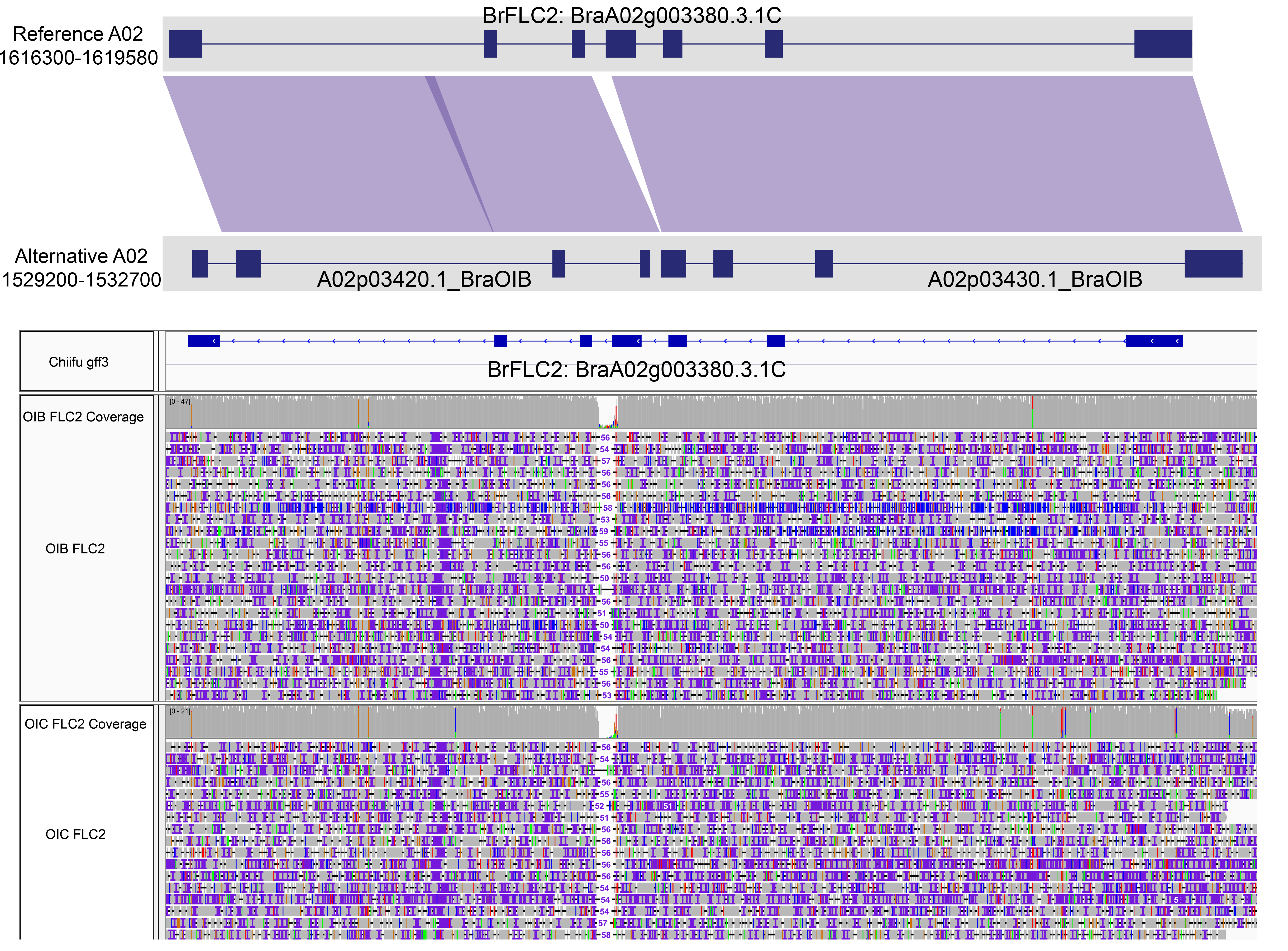


**Fig. S26** A 55-bp structural variation occurred in the *BrFLC2* region. **a**, Micro-synteny analysis between the two genotypes of *BrFLC2*. **b**, The PacBio reads of OIB and OIC were aligned to the *BrFLC2* gene region on the Chiifu reference genome.


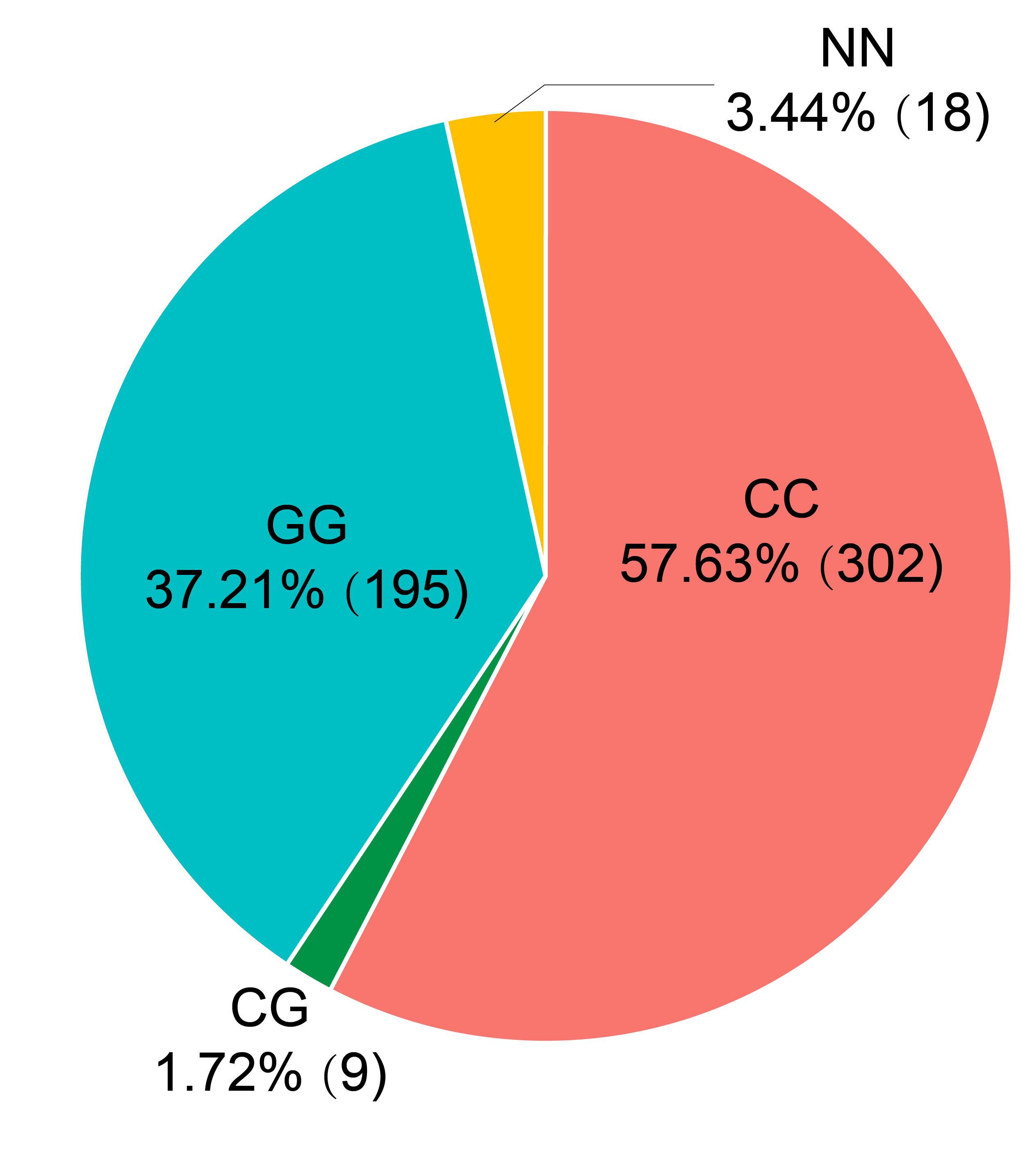


**Fig. S27** The genotype of the structural variation in the *BrPIN3.3* gene region in 524 accessions. CC indicated that the genotype in the corresponding accession was consistent with the reference genome, and GG indicated that the genotype in the accession was different from the reference genome. CG represents the heterozygous type, and NN represents a deletion.


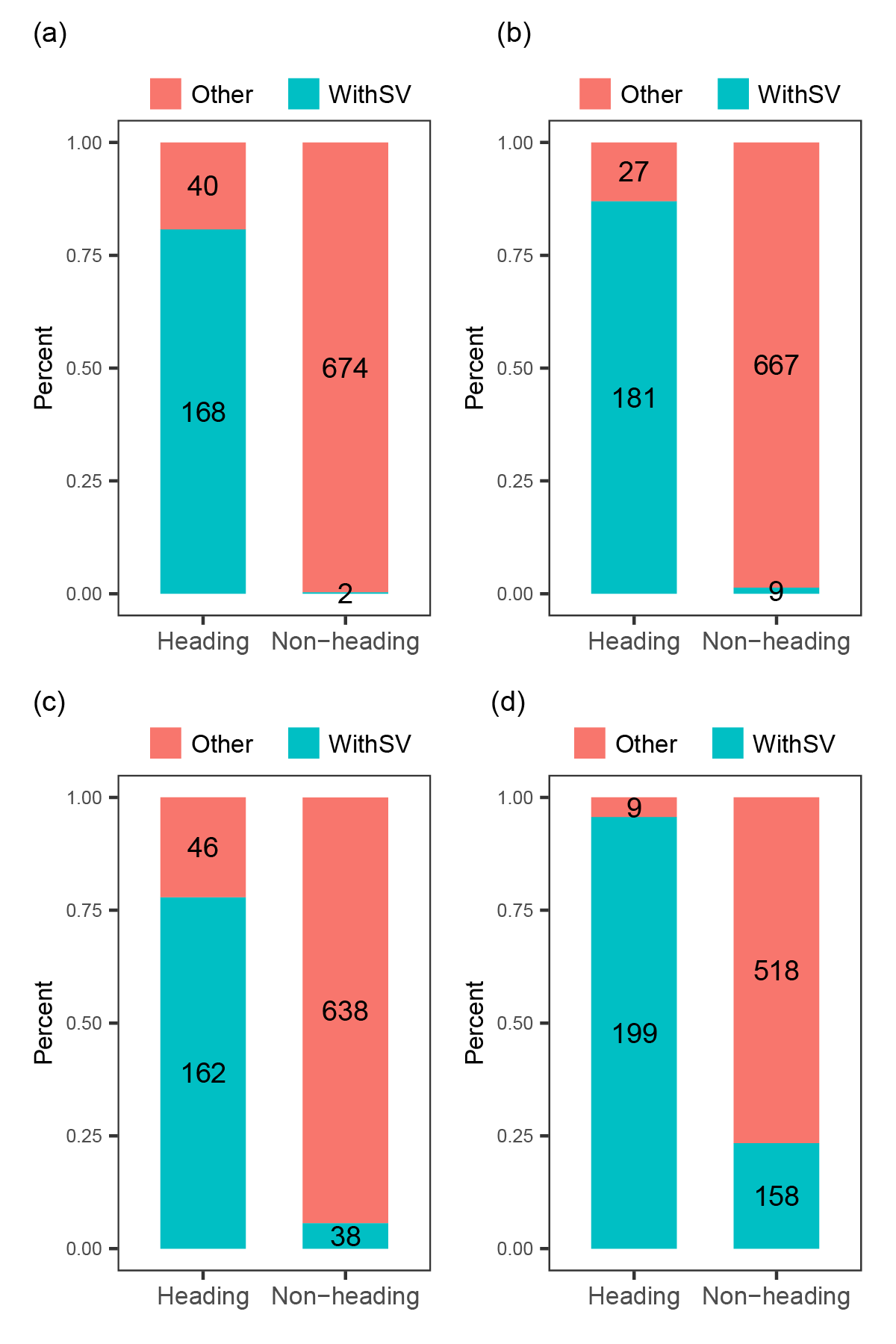


**Fig. S28** The SVs in the four candidate genes are genotyped in a larger *B. rapa* collection of 884 accessions. WithSV represents the dominant allele in the heading population. The SVs in the four candidate genes are genotyped in a large *B. rapa* population. The primers are recorded in Table S31, **a**, **b**, **c**, and **d** show the results of SVs in the *BrPIN3.3*, *BrMYB95.3*, *BrFL5.1*, and *BrSAL4.2* genes, respectively. Genotypes of these markers are collected separately for the heading and non-heading accessions and are submitted to Fisher’s exact test using R software (Table S36).


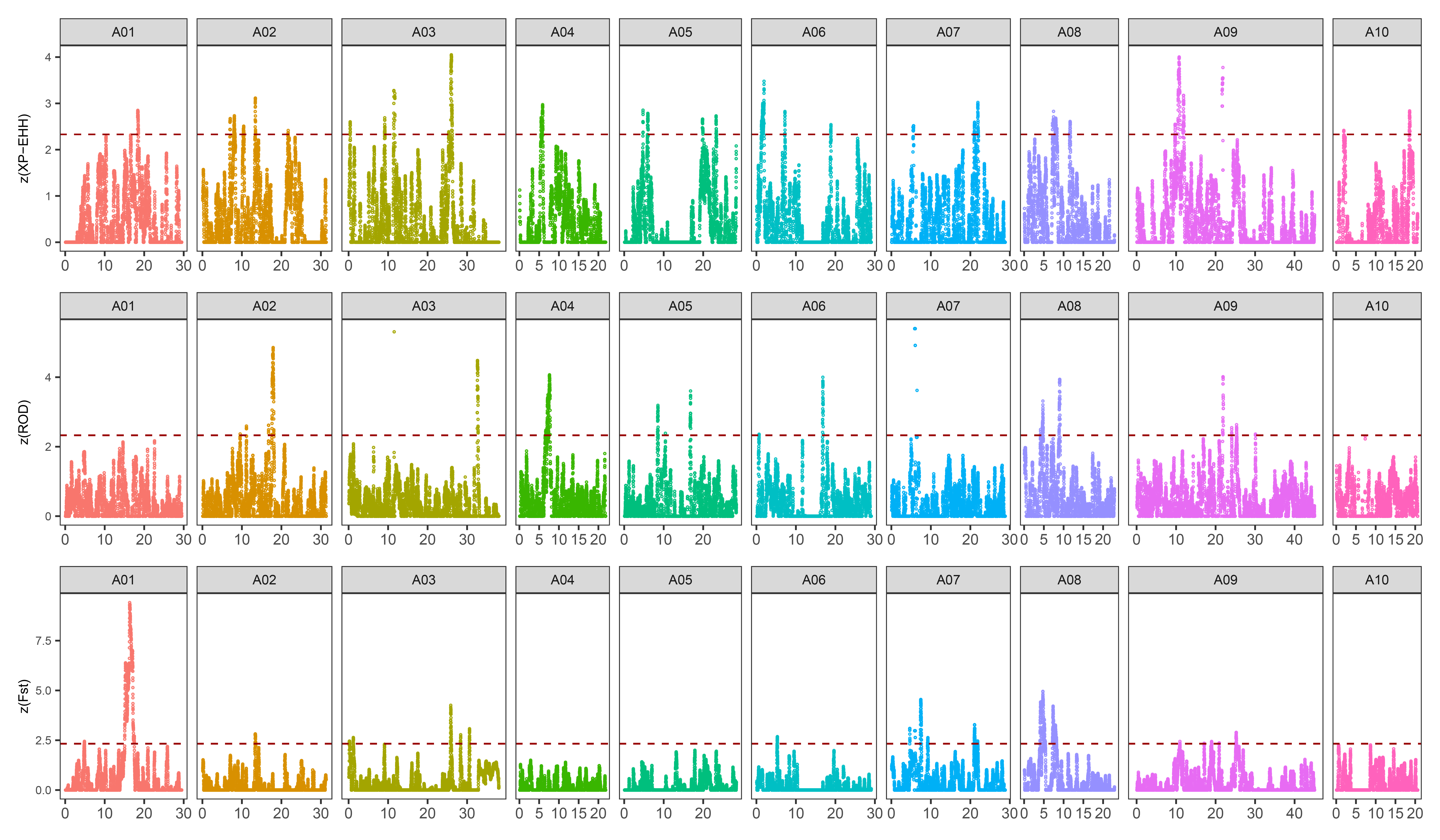


**Fig. S29** Genomic signatures of selection in the genome of Chinese cabbage. The XP-EHH, ROD, and Fst values were normalized as z scores for *B. rapa*. A 200–kb sliding window with an increment of 5 kb was used to calculate these values; each point represents a value in a 200–kb window. The horizontal dashed lines show the empirical threshold of α = 0.01 (z = 2.33).





**Fig. S30** Structural Variation of *BrMYB95.3* is associated with heading morphotype diversification. **a**, The distribution of haplotypes in *BrMYB95.3* gene region in 524 accessions. **b**, The distribution of one of the *BrMYB95.3* genotypes in 524 accessions. Accessions with a 74 bp deletion in *BrMYB95.3* were marked using red stars. **c**, Micro-synteny analysis between the two genotypes of *BrMYB95.3*. **d**, Fst values were normalized as z scores for *B. rapa* on A07. A 200-kb sliding window with an increment of 5 kb was used to calculate the normalized Fst value. Each point represents a value in a 200-kb window, and the horizontal dashed line presented the empirical threshold of α = 0.01 (z = 2.33). The arrow indicated the location of the *BrMYB95.3* gene. **e**, Expression level of *BrMYB95.3* in 44 heading and 42 non-heading accessions. CC (Chinese cabbage) and Others represented heading and non-heading types, respectively. **f**, The genotype of SV in the *BrMYB95.3* gene region in 524 accessions. CC indicated that the genotype in the corresponding accession was consistent with the reference genome, and GG indicated that the genotype in the accession was different from the reference genome. CG represents the heterozygous type, and NN represented deletion.


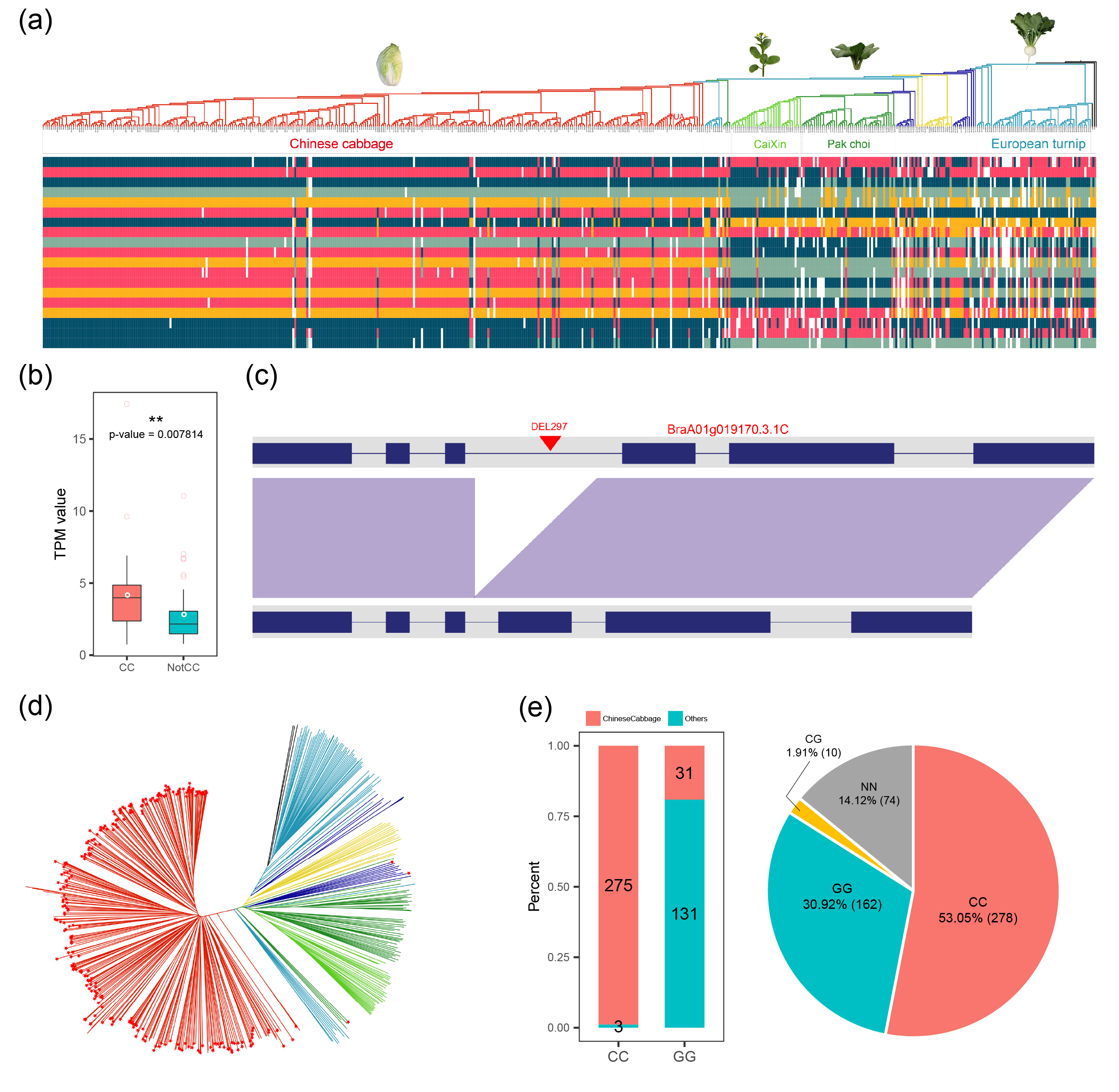


**Fig. S31** Structural Variation of *BrFL5.1* is associated with heading morphotype diversification in *B. rapa*. **a**, The distribution of haplotypes in *BrFL5.1* gene region in 524 accessions. **b**, Expression level of *BrFL5.1* in 44 heading and 42 non-heading accessions. **c**, Micro-synteny analysis between the two genotypes of *BrFL5.1*. **d**, The distribution of one of the *BrFL5.1* genotype in 524 accessions. **e**, The genotype of SV in the *BrFL5.1* gene region in 524 accessions.


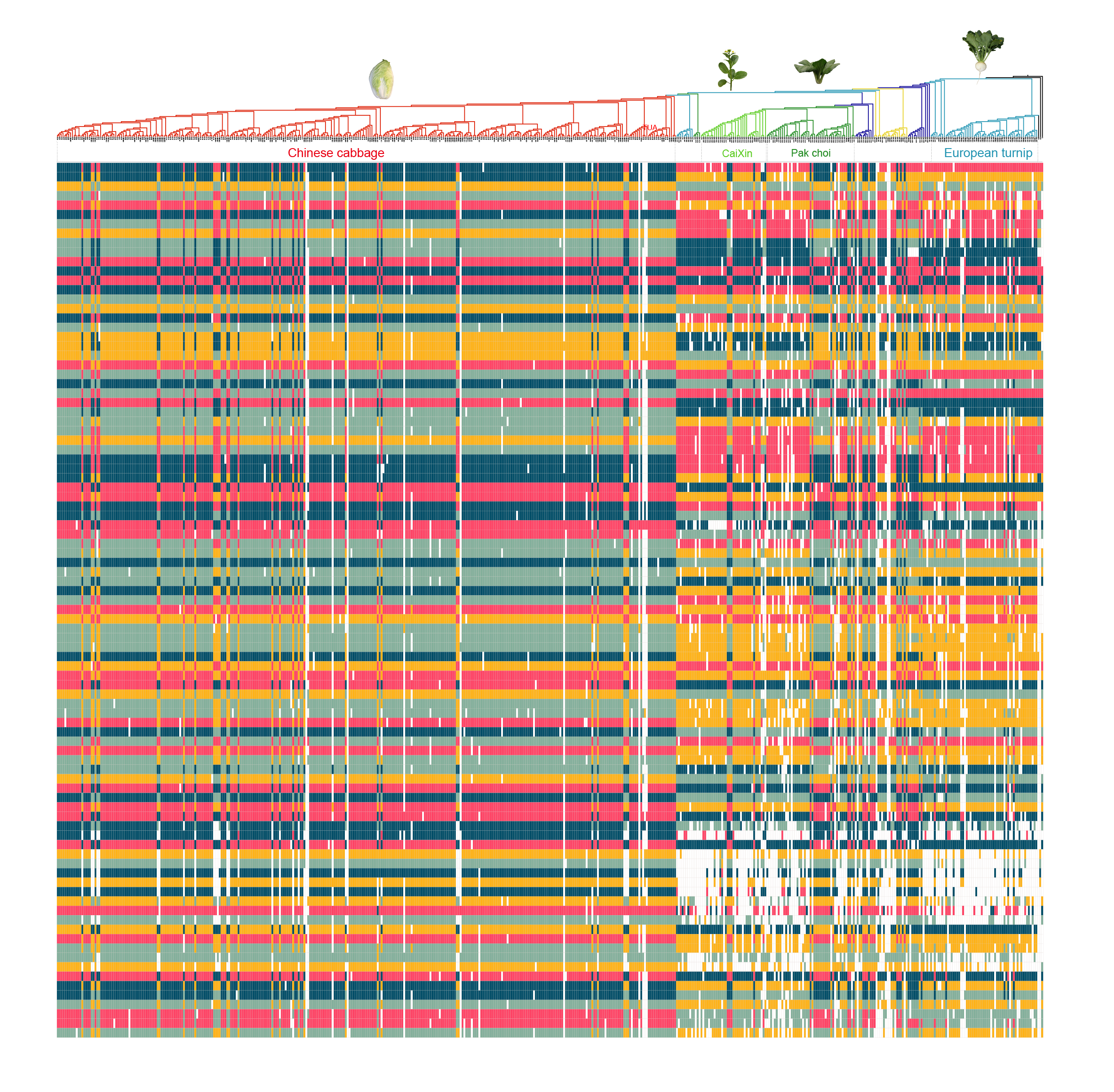


**Fig. S32** The distribution of haplotypes in *BrSAL4.1* gene region in 524 accessions.


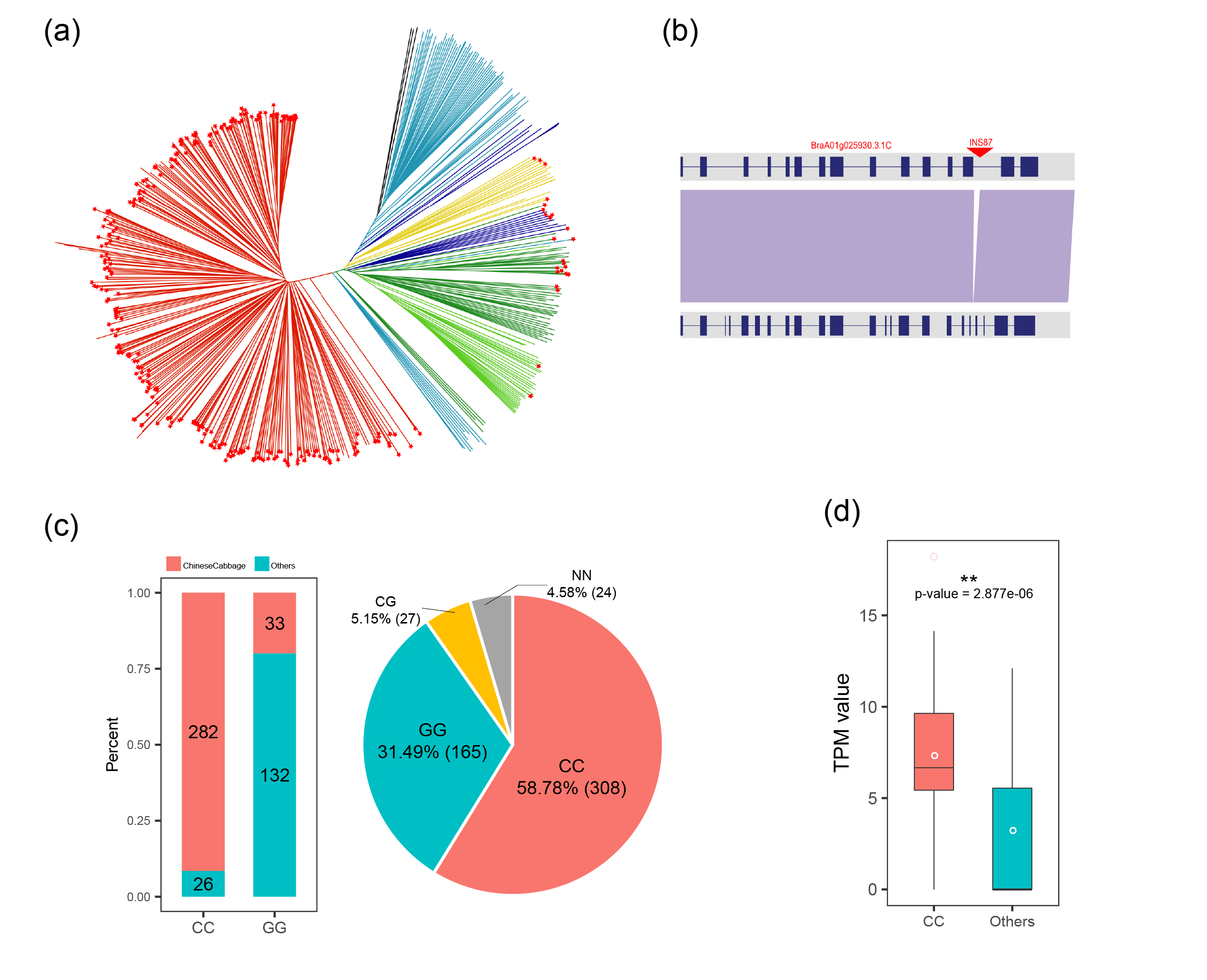


**Fig. S33** Structural Variation of *BrSAL4.1* is associated with heading morphotype diversification in *B. rapa*. **a**, The distribution of one of the *BrSAL4.1* genotype in 524 accessions. **b**, Micro-synteny analysis between the two genotypes of *BrSAL4.1.* **c**, The genotype of SV in the *BrSAL4.1* gene region in 524 accessions. **d**, Expression level of *BrSAL4.1* in 44 heading and 42 non-heading accessions.


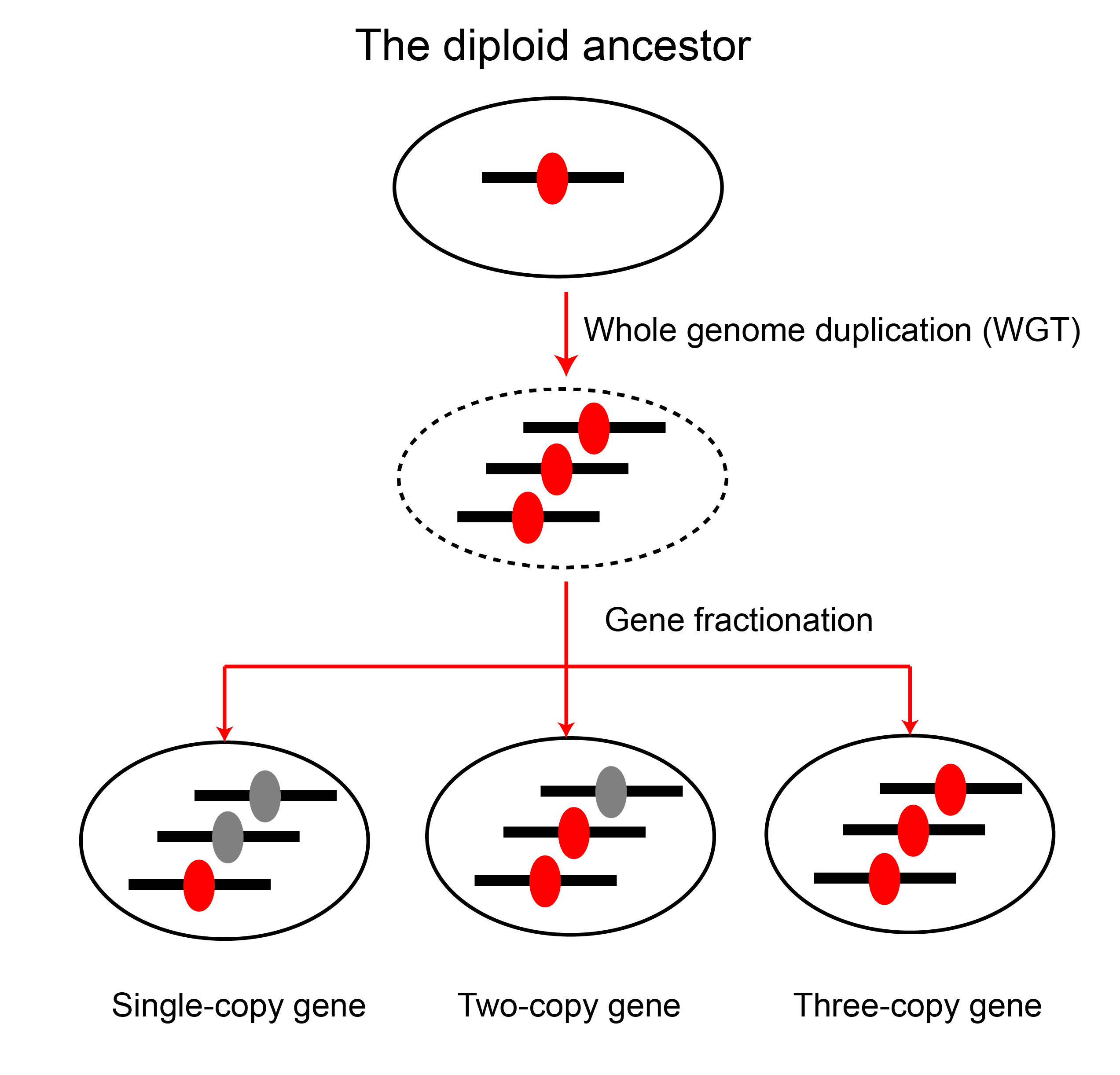


**Fig. S34** The definition of single-, two- and three-copy genes in *B. rapa*


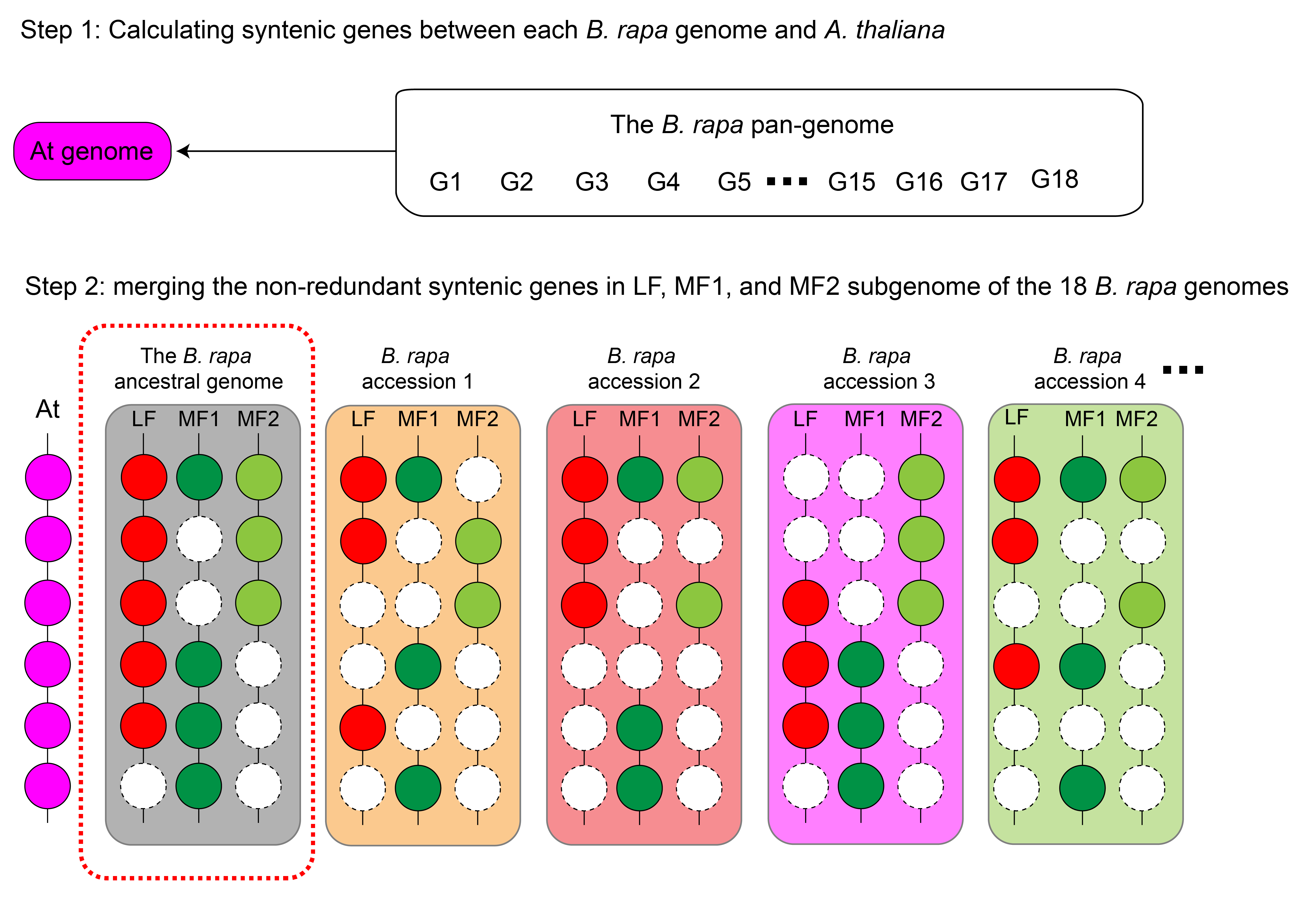


**Fig. S35** The pipeline of constructing the *B. rapa* ancestral genome
