# Supplementary material for: Impacts of allopolyploidization and structural variation on intraspecific diversification in Brassica rapa
Source: Genome Biol. 2021 May 31;22:166. doi: 10.1186/s13059-021-02383-2 (PMC8166115; doi:10.1186/s13059-021-02383-2)
Supplement: Supplementary file 6 — Additional file 6. Review history. [file 13059_2021_2383_MOESM6_ESM.docx]

Review History

**First round of review**

**Reviewer 1**

**Are you able to assess all statistics in the manuscript, including the appropriateness of statistical tests used?**

Yes.

**Comments to author:**

This study assembled 16 representative B. rapa genomes and then constructed a pan-genome of B. rapa (with these 16 B. rapa genome and 2 published B. rapa genome). They demonstrate good application of the pan-genome in B. rapa:

1. Divided all gene families into 4 different classes: core, softcore, dispensable, and private.

2. Identify gene flexibility with pan-genome and analysis of subgenome dominance in aspect of gene flexibility.

3. Construct common ancestor genome of Brassiceae species (ABrassiceae) and common ancestral genome of B. rapa and B. oleracea (ABra_Bol) with pan-genome, to reveal intraspecific diversification and the continuing influence of subgenome dominance in fraction in two evolution stage.

4. Identify SVs within individual B. rapa with pan-genome, and reveal intraspecific morphotype diversification associated with SVs. For example, based on identification, they observed four SV-related genes involved in the domestication of the B. rapa leafy head.

The present paper shows meaning of pan-genome in evolution analysis and trait-related gene identification. Please see the comments below.

Major comments:

1. The current analysis of SV and flexible syntenic genes looks more like two independent work. It is recommended to increase the relationship analysis between SV and flexible genes to make this study more systematic and complete.

(1) Flexible syntenic genes mainly come from large-scale deletion of the genome (PAV) or non-functionalization of duplicate genes accumulation of small mutations (accumulation of small mutations).

(2) Please add a correlation analysis of the distribution of polyploidy-derived flexible syntenic genes at the gene level, such as whether they are randomly distributed or clustered within a single LF/MF block(refer to Lou et al., Plant Cell, 2012;24;2415-2426).

2. The authors use different standards when defining core/softcore/dispensable/private genes and CSGs/FSGs. Please use the same standard.

3. The authors need to make it clear that the concept and identification method of single-, two- and three-copy gene sets.

4. In the "high flexibility of multi-copy genes" analysis section. Considering WGD/WGT-derived duplication and gene dosage hypothesis, it is recommended that the author divide multi-copy genes into WGT-derived multi-copy genes, SSD-derived multi-copy genes or dose-sensitive genes.

5. The description in methods is too general. These can cause great difficulty for others to do similar analysis. For example, authors didn't list versions of many softwares used in this study, for example, MaSuRCA, RepeatMasker, AUGUSTUS, GeneMark and so on.

On Page 32, "First, single-copy genes between B. oleracea and the 18 B. rapa were determined by OrthoFinder", but didn't list which genome assembly of B. oleracea used, To1000, HDEM or others?

On Page 33, line 15-16: Authors introduced the analysis methods of SNPs and InDels, but just said "Finally, SAMtools was used to filter the PCR duplicates of the reads and call SNP variants. We also used Graphtyper to call InDels", which is general and authors should describe detailed steps and parameters about the identification of SNPs and InDels and how to integrate results from SAMtools and Graphtyper.

On Page 33, line 7-9: "We used MUMmer to align the 17 assemblies to the B. rapa reference genome (Chiifu) using the parameters '--mum -g 1000 -c 90 -l 40', following which we used the delta-filter with parameters settings '-r -q' to obtain one-to-one blocks in the alignment results". Maybe authors used Nucmer program of MUMmer package and if authors want to obtain one-to-one blocks, authors should use the parameter '-1'. Authors should The author can refer to this article(Zhang, J., Chen, L.L., Xing, F., Kudrna, D.A., Yao, W., Copetti, D., Mu, T., Li, W., Song, J.M., and Xie, W. (2016). Extensive sequence divergence between the reference genomes of two elite indica rice varieties Zhenshan 97 and Minghui 63. Proc Natl Acad Sci U S A 113:E5163.)

Authors should polish the description of the methods so that the readers can understand and repeat the methods used by the authors.

6. Sizes of genome assemblies ranged 337.46 Mb to 466.50 Mb and contig N50 sizes ranged 0.25Mb to 1.41 Mb why do genome sizes and contig N50 sizes vary so much? Is it related to the heterozygosity of these genomes? Authors should explain it.

Minor comments

1. On page 10, line 17-19: "The insertions of LTR-RTs were more likely to occur in the dispensable genes". Authors only counted the ratio of genes with LTR-RTs in intron regions in the core, softcore, and dispensable genes in Figure 1h. Coding region and promoter region should be counted and compared.

2. On Page 13, line 1-3: "We observed that nearly 50% of SVs involved repetitive sequences (Fig. 2e), and SVs were tightly associated with morphological diversification (Supplementary note)." If repetitive sequences cover 46.52% of the Chiifu genome, 50% of SVs involved repetitive sequences may be not very high. Authors should compare SV regions with genome regions selected randomly.

3. The legend for multiple tests should be more detailed (e.g., Fig. 1e-f)

4. Spelling mistake. (e.g., hist2 in Line 16 on Page 34 should be hisat2.)

5. On Page 16, line 13-16: "We found that an average of 10.06% and 3.47% of two copies were least and more FSGs (Fig. S21), and an average of 7.77%, 3.35%, and 1.86% of three copies were least, more and most FSGs, respectively (Fig. 3i and Table S26)" and I didn't see Fig. 3i and I think it's Fig. 3g.

6. In Fig.6a, it should be better to use SV genotypes.

**Reviewer 2**

**Are you able to assess all statistics in the manuscript, including the appropriateness of statistical tests used?**

No, I do not feel adequately qualified to assess the statistics.

**Comments to author:**

This is a valuable study on the impacts of (allo)polyploidisation on diversification in Brassica rapa and will be of broad interest. The dataset is phenomenal in scale and the analysis appears solid, but the presentation needs some improvement. I have given many suggestions to increase clarity.

Aside from issues with the writing (which I work to enumerate at length below), I do have a couple of broader concerns with the paper:

1. Overall the scope of this entire paper is limited to allopolyploids, but the authors uniformly use the much wider term 'polyploids' throughout. This is not accurate and overblows the import of the work: given the lack of hybridisation (and thus subgenomes), 'pure polyploids,' autopolyploids, do not engage in the phenomena discussed here (they don't have homeologs, subgenome dominance, etc). Therefore the authors should consistently change their terminology to add the prefix 'allo-' to 'polyploid' throughout. Further, the distinction between these two major types of polyploids should be made clear up front in the introduction.

2. Given the amazing data it seems a missed opportunity that the authors use only SV to identify genomic correlates of morphotype diversity. Why not compare to the SNP data? If the goal is to get a realistic picture of the genomic changes controlling phenotype, looking only at SVs makes little sense and should be paired with an analysis of the SNP polymorphisms, which relative to the SV analysis, would be straightforward, especially given the authors are focussing only on the heading phenotype. The MS as currently presented leaves the reader wondering whether the given SV analysis is more powerful than one using SNPs in the same dataset and additionally how many genes are missed in the presented approach. Surely some SNPs are causative, and the SV used here are imperfect linked markers for the causative SNP. If on the other hand the given SV analysis is more powerful for finding associated genetic changes than using SNPs, that would be fascinating to see. Would it not be best to have both complementary methods, given the data are there and this is a golden opportunity to compare the normal standard with this new unique SV-based analysis? This would also give a fuller picture of the true genomic basis for the traits of interest. The authors are part-way there with the XP-EHH analysis shown over one region; can this not be formalised in addition to a SNP-based GWAS and compared to the SV results to show whether indeed this SV approach indeed finds more candidates than mapping SNPs to a single reference (or a pangenome graph, as the authors prefer)?

3. Thirdly, the authors state (e.g. P24ln7) that 'gene flexibility during intraspecific diversification increased the subgenome dominance'. How do the authors feel causality is established in this relationship? This needs to be better stated (justified) for the reader. That is, why do they think that subgenome dominance does not cause the 'flexibility'? A reader could imagine, for example, that highly unequal expression levels of two homeologs would lead to stronger purifying selection on the highly expressed one, and much lower purifying selection on the less strongly expressed one. On account of this difference in expression, the low expressed one to accumulate diversity. This is in strong contrast to the (purported causal) 'flexibilty' the authors assume. This may be a lack of knowledge on my part, but as a reader of the document, I am not at all convinced causality is established in this assumed relationship.

Other points:

Abstract ln7-8

"Among the genes derived from WGT, 13.42% of polyploidy-derived genes accumulated more transposable elements" More than what? More than others? This is clear.

Abstract ln17

266 candidate genes for what? Domestication genes with SV?

P3ln6

"subgenome dominance is a common phenomenon that is widely observed in polyploids" The authors must distinguish first between allo- and autopolyploids. Subgenome dominance is not a common phenomenon across all polyploids: it is only evident in allos.

P3ln15

'ancient' is too specific: diversification events following WGD occur within 10,000 -20,000 generations, for example, which is much less ancient than those to which the authors are perhaps pointing.

P4ln4

'620 homoeologous pairs that have been subjected to the domestication of long white fiber' This is unclear as written. The pairs have been subjected to the domestication? Perhaps this is colloquial in crop breeding, but not broadly accessible usage.

P4lLn8-11

Please be more specific: long read pop resequencing can achieve this, just not short read.

P4ln22

Most readers will have an idea of the A.thaliana genome size, but it should be stated here if the authors move away from giving percentages to giving Mb in this example (inexplicably to the reader). It would be better to keep examples consistent and use percentages, as in all the preceding.

P5ln22 'crucial' is not the correct word here. I would suggest 'powerful' or similar.

P6ln7 "correlations of SVs with intraspecific morphotype domestication are still unclear," or do you mean 'there are no studies of…'? if they are unclear it suggests there's some knowledge but the correlation itself is unclear, which does not seem right. Please rephrase.

P6ln12 'extensively identified' seems rather fluffed up. Suggest striking 'extensively'

While generally very good, sometimes the language can still be polished: 'the core gene represents'. I suggest to change that to 'core genes are defined as genes that were retained' and the parallel construction in the rest of the sentence.

Ln 16 'correlated with subgenome dominance' Do the authors mean 'the extent of subgenome dominance'?

P7ln15 'for genome annotation' I suggest to strike or rephrase.

P8ln1 'Multiple' or more accurately 'All'? Also, 'variations' would be better as 'variants' here and elsewhere.

P8ln5 'relatively short period' begs the question 'how long.' It would be good to give some estimate for the reader.

Ln9 'variations' (see above)

Table 1å: 'statistics' More accurately these are 'metrics'

P9ln7 'closed pan-genome': for the readers who don't read a lot of pangenome literature please explain what you mean by this uncommon term more explicitly (though obviously the next sentence follows the statement)

P10ln1 what is the difference here between orphan genes and those present in only one accession? Are private genes where both homeologs are present in one accession and orphans where only one homeolog is present in one accession? If so, please make more clear for readers with even less allopolyploid orientation than myself

P10ln5: some of the writing could still be cleaned up. For example, rephrasing "The proportion of genes with large effect mutations in core genes" to something like "The proportion of core genes with large effect mutations" etc.

P11ln14 here, for example, 'variations' should just be 'variation'

P11ln16 'in different chromosomes and the whole-genome' simplify to something like 'across the genome'

P12ln6 'Large-scale resequencing illustrated the high representation of identifying genomic variations using the B. rapa pan-genome' Please rephrase completely; this makes little sense to the reader.

P12ln16 'further supporting the high representation of identifying genomic variations using the pan-genome' Totally unclear to me what this means here.

P13ln1 'species' I am no expert on Brassicas, but is it right to call these different species of the species Brassica rapa?

P13ln4 'the nonredundant SV set started to slow down and tended to flatten (Fig. 2f)' Something is missing from this sentence. Please add something like 'with the addition of more species. I note 'started to slow down and tended to flatten' is a bit strange usage here.

P14ln10: this section heading is not proven by the following text. It's a nice speculation but not appropriate as a section heading. In what way does the study test if anything is a 'driving force'?

P14ln13 "The gene flexibility represented a gene was fractionated in some B.rapa genomes." Please rephrase; this makes little sense to the reader.

Ln16 'In this study, if a gene had an orthologous gene in A. thaliana' or more simply 'an ortholog in A. th..'

P15ln9 nonsyn and large effect mutations do not equal positive selection and the inference of causality ('driving force' in the section heading) in this section is unsupported. Please rephrase.

Ln 10: in what way exactly do the authors mean, strictly speaking, that 'FSGs were strongly correlated with the intraspecific diversification'? This entire section should be reworked.

P15ln16: it is not enough to bring up 'the two-step theory' as the authors do: there should please be a brief description of what they're talking about here.

P15ln21: Fig S24 callout here is an error?

Here also: 'revealing that the biased gene flexibility during intraspecific diversification increased the dominance of the LF subgenome' The authors cannot prove causality here and are simply measuring a correlation. This should be rephrased accordingly throughout.

P16ln3: 'further highlighting the continuing influence of the dominant subgenome on intraspecific diversification.' I do not see how causality is proven here and therefore the authors need to either explain this clearly or change their language to state correlation instead.

P16ln17 does gene duplication actually 'reduce the pressure of effective purifying selection'? This seems an odd formulation. That is, the selection pressure is the same; I'd just see it as managed differently by the duplicates.

P17ln5 use of parenthesis is awkward such as 'Conserved (Flexible) represent conserved (flexible) syntenic gene in the homoeologous pair.' Rephrase please.

P17ln15 "In total, there were 30,166 genes in the B. rapa ancestral" The language here is too careless: there were 30,166 genes in the inferred B. rapa ancestral genome. The number in the actual ancestral genome is unknown. Please rephrase with more care. Later invocations (final line on page/first line of p18, for example) should also be given the adjective 'inferred' to 'ancestral.'

Ln18 'revealing' The language is a too strong and somewhat careless. Suggest 'indicating' or 'suggesting'. The evidence given is hardly conclusive enough to 'reveal'

P18: Please describe exactly what 'gene density' measures and how it is calculated at the first mention.

P18ln4-7: two sentences merged in run-on. Rephrase.

P18ln11: delete 'the' in 'the B. nigra'

P18: again, the discussion here focusses on INFERRED ancestral genomes and the gene numbers are low estimate for the ACUTAL gene numbers in the ancestral genomes. The language in the present draft does not take this into account and needs to be clearer throughout.

Fig5: spell out R. sativus genus please.

P20ln13; FigS22 shows a single example re the 74bp deletion tested, but Table S31 gives many primer pairs. None of the other pairs or data are described. Why not?

P20ln17 "especially for the leaf heading…" rephrase; this makes no sense.

P24ln9 "the genomes because more different" When? Please rephrase and clarify.

The discussion (like the entire paper) is very long and should be trimmed dramatically for readability. Much of it is redundant with previous statements in the manuscript.

P24ln16 when stating a sentence spell the genus; here it's abbreviated.

P25Ln12 why TE's all of a sudden? The discussion seems rambling and a bit disorganised.

**Authors Response**

**Point-by-point responses to the reviewers’ comments:**

Reviewer #1:

This study assembled 16 representative B. rapa genomes and then constructed a pan-genome of B. rapa (with these 16 B. rapa genome and 2 published B. rapa genome). They demonstrate good application of the pan-genome in B. rapa: 1. Divided all gene families into 4 different classes: core, softcore, dispensable, and private. 2. Identify gene flexibility with pan-genome and analysis of subgenome dominance in aspect of gene flexibility. 3. Construct common ancestor genome of Brassiceae species (ABrassiceae) and common ancestral genome of B. rapa and B. oleracea (ABra_Bol) with pan-genome, to reveal intraspecific diversification and the continuing influence of subgenome dominance in fraction in two evolution stage. 4. Identify SVs within individual B. rapa with pan-genome, and reveal intraspecific morphotype diversification associated with SVs. For example, based on identification, they observed four SV-related genes involved in the domestication of the B. rapa leafy head. The present paper shows meaning of pan-genome in evolution analysis and traitrelated gene identification. Please see the comments below.

Q1. The current analysis of SV and flexible syntenic genes looks more like two independent work. It is recommended to increase the relationship analysis between SV and flexible genes to make this study more systematic and complete.

*Response: We agree. In the revised manuscript, we have added the relationship analysis between SV and flexible genes (Page 15, Line 6–7, Fig. S19). We found that the ratio of SV-genes in FSGs was significantly higher than CSGs (P = 9.1e-15). Furthermore, we compared the impact of small mutations to the impact of SVs, and we found that genes with large-effect mutations mainly originated from the accumulation of small mutations (for details please refer to A2).*

*Fig. S19. Ratio of SV-genes in CSGs and FSGs. SV-gene represents that an SV occurred in the gene region.*

Q2: (1) Flexible syntenic genes mainly come from large-scale deletion of the genome (PAV) or non-functionalization of duplicate genes accumulation of small mutations (accumulation of small mutations).

*Response: We found that flexible syntenic genes are mainly the result of the accumulation of small mutations. However, as this result was not the main point of our manuscript, we did not add this result in the revised manuscript. However, since the reviewer is interested in this aspect, we have provided the following details. We calculated the ratio of genes with large-effect mutations that were influenced by small mutations and SVs in FSGs and CSGs to evaluate the impacts of small mutations and SVs on gene flexibility. We defined large-effect mutations to include small mutations (SNPs and InDels) that lead to start-codon mutations, stop-codon mutations, and premature stop codons, etc. (Sun et al., 2018) and SVs that occur in the CDS regions. The average ratios of genes with large-effect mutations influenced by small mutations and SVs were 21.38% and 11.05% in FSGs, and this ratio was 18.01% and 4.47% in CSGs, respectively. The ratios of genes with large-effect mutations influenced by small mutations were significantly higher than those influenced by SVs in both FSGs and CSGs (P = 5.0e-14 and P = 9.4e-16), revealing that flexible syntenic genes mainly result from the accumulation of small mutations.*

*Sun et al., 2018; Extensive intraspecific gene order and gene structural variations between Mo17 and other maize genomes Figure. Ratio of genes with large effect mutations in FSGs (a) and CSGs (b).*

Q3: (2) Please add a correlation analysis of the distribution of polyploidy-derived flexible syntenic genes at the gene level, such as whether they are randomly distributed or clustered within a single LF/MF block (refer to Lou et al., Plant Cell, 2012;24;2415-2426).

*Response: We agree. In the revised manuscript, we calculated the distribution of FSGs across the B. rapa three subgenomes. Using the Chiifu genome as the reference, we identified genomic blocks and calculating FSG density across the three subgenomes at the gene level (Fig. S22). We observed that the FSGs did not cluster together within a single block.*

*Fig. S22. Distribution of FSGs across B. rapa three subgenomes. A 50 genes sliding window with an increment of 20 genes is adopted to calculate the FSG density in the Chiifu three subgenome. Red indicates the density of FSGs.*

Q4: The authors use different standards when defining core/softcore/dispensable/private genes and CSGs/FSGs. Please use the same standard.

*Response: “CSGs/FSGs” was defined to investigate the evolution of WGT-derived genes during intraspecific diversification. For this, we had to trace the syntenic relationship for each of the B. rapa genes with that of A. thaliana. However, “core/softcore/dispensable/private genes” was defined by the frequency of gene families in the 18 B. rapa genomes, as it ignores the syntenic relationship of the genes between B. rapa and A. thaliana. Therefore, it will be confusing if we follow the same standard to define “CSGs/FSGs”. Additionally, we used a very strict definition of "CSGs/FSGs", in which each of the CSGs and FSGs is supported by at least two sequenced genomes. This definition allowed us to avoid introducing wrong gene models into the CSG/FSG sets due to genome assembly or gene model predicting errors.*

Q5: The authors need to make it clear that the concept and identification method of single-, two- and three-copy gene sets.

*Response: We thank the reviewer for pointing this out. We have made a supplementary figure to explain this in the revised manuscript (Page 35 Line 19–20, Fig. S34). We define single-, two- and three-copy gene sets as below: If one gene in A. thalina had one orthologous gene in B. rapa, then the orthologous gene was defined as a single-copy gene; if one gene in A. thalina had two orthologous genes in B. rapa and the two orthologous genes were distributed on two of the three subgenomes, then the two orthologous genes were defined as two-copy genes; if one gene in A. thalina had three orthologous genes in B. rapa and the three orthologous genes were distributed on the three subgenomes, then the three orthologous genes were defined as three-copy genes; Additionally, we have added details of the method for identifying the three gene types (Page 35, Line 19–20).*

*Fig. S34. The definition of single-, two- and three-copy genes in B. rapa.*

Q6: In the "high flexibility of multi-copy genes" analysis section. Considering WGD/WGT-derived duplication and gene dosage hypothesis, it is recommended that the author divide multi-copy genes into WGT-derived multi-copy genes, SSD-derived multi-copy genes or dose-sensitive genes.

*Response: In the present analysis, we did not divide multi-copy genes into WGT-derived multicopy genes and SSD-derived multi-copy genes, as all multi-copy genes were WGTderived multi-copy genes. We have added Fig. S34 to explain the multi-copy genes we used in our work (for details please refer to A5).*

Q7: The description in methods is too general. These can cause great difficulty for others to do similar analysis. For example, authors didn't list versions of many softwares used in this study, for example, MaSuRCA, RepeatMasker, AUGUSTUS, GeneMark and so on.

*Response: We thank the reviewer for pointing this out. We have added version information, parameters, and detailed pipelines in the revised manuscript.*

Q8: On Page 32, "First, single-copy genes between B. oleracea and the 18 B. rapa were determined by OrthoFinder", but didn't list which genome assembly of B. oleracea used, To1000, HDEM or others?

*Response: We used our newly assembled Brassica oleracea JZS v2.0 (Cai et al., 2020).*

*Cai et al., 2020; Improved Brassica oleracea JZS assembly reveals significant changing of LTR-RT dynamics in different morphotypes.*

Q9: On Page 33, line 15-16: Authors introduced the analysis methods of SNPs and InDels, but just said "Finally, SAMtools was used to filter the PCR duplicates of the reads and call SNP variants. We also used Graphtyper to call InDels", which is general and authors should describe detailed steps and parameters about the identification of SNPs and InDels and how to integrate results from SAMtools and Graphtyper.

*Response: We have added the version information and parameters of these software programs in the revised manuscript. We have also described the steps and parameters in detail (Page 33, Line 17–22).*

Q10: On Page 33, line 7-9: "We used MUMmer to align the 17 assemblies to the B. rapa reference genome (Chiifu) using the parameters '--mum -g 1000 -c 90 -l 40', following which we used the delta-filter with parameters settings '-r -q' to obtain one-toone blocks in the alignment results". Maybe authors used Nucmer program of MUMmer package and if authors want to obtain one-to-one blocks, authors should use the parameter '-1'. Authors should The author can refer to this article(Zhang, J., Chen, L.L., Xing, F., Kudrna, D.A., Yao, W., Copetti, D., Mu, T., Li, W., Song, J.M., and Xie, W. (2016). Extensive sequence divergence between the reference genomes of two elite indica rice varieties Zhenshan 97 and Minghui 63. Proc Natl Acad Sci U S A 113:E5163.)

*Response: Yes, we used the Nucmer program from the MUMmer package to conduct the alignment. We have changed " MUMmer" into " Nucmer program" in the revised manuscript.*

*We have checked the parameter '-1' and the parameter '-r -q' in the 'delta-filter' manual, which indicated that the result of using the parameter '-1' is the intersection of the -r and -q alignments. The parameter '-r -q' is also a common means of detecting one-toone blocks. The same description was also used in the latest Brassica napus pangenome analysis (Song et al., 2020). We have changed the parameter into '-1' in the revised manuscript (Page 33; Line 11).*

*Song et al., 2020; Eight high-quality genomes reveal pan-genome architecture and ecotype differentiation of Brassica napus).*

Q11: Authors should polish the description of the methods so that the readers can understand and repeat the methods used by the authors.

*Response: We have carefully adjusted the description of the methods in the revised manuscript as suggested. Firstly, we have added the version information of the software; Secondly, we have added the parameters of the software; and thirdly, we have added detailed steps in the method.*

Q12: Sizes of genome assemblies ranged 337.46 Mb to 466.50 Mb and contig N50 sizes ranged 0.25Mb to 1.41 Mb why do genome sizes and contig N50 sizes vary so much?

*Response: We found that the final assembly sizes were affected by the genome composition and the integrity of the TE sequence assembly. The contig N50 sizes were affected by the sequencing depth and heterozygosity of these genomes.*

*The genome composition and integrity of the TE sequence assembly affects the final assembly sizes. First, using flow cytometry, Boutte et al (Boutte et al., 2020) found a genome size variation of up to 16% between different B. rapa accessions; specifically, they confirmed a length difference of 17.6% between the A06 chromosomes of two B. rapa accessions. Second, in our work, the length of TEs in each genome was 148.86–249.16 Mb, and the maximum difference in the length of the assembled TE sequences was 100.3 Mb in the different genomes. Additionally, we found that the TE content was positively correlated with the genome assembly size (R = 0.99, P = 3.8e16). Therefore, we believe that both the genome composition and the integrity of the TE sequence assembly are associated with the final assembled sizes. The differences in Contig N50 may be associated with the long-read sequencing depth and the heterozygosity of these genomes. It is known that the Contig N50 value can be improved by using more long-read sequencing reads. Also, we think that the heterozygosity can affect the contig N50 value (for details please refer A13).*

*Boutte et al., 2020; Genome Size Variation and Comparative Genomics Reveal Intraspecific Diversity in Brassica rapa.*

Q13: Is it related to the heterozygosity of these genomes? Authors should explain it.

*Response: Yes, the contig N50 values can be influenced by the heterozygosity of these genomes. We used short reads to evaluate the heterozygosity of the de novo assembled 16 B. rapa genomes, and found that the accession with high heterozygosity had a low contig N50 value. For example, the contig N50 values of MIZ and CCB were 0.25 Mb and 1.4Mb，and the corresponding genome heterozygosity was 0.59% and 0.15%, respectively.*

*Table. Correlation analysis of assembly size, contig N50, and heterozygosity*

*IndexAssembly size*

*(Mb)Heterozygosity*

*(%)Contig N50 (kb)*

*CCA341.620.19689*

*CCB378.470.151,413*

*CXA378.750.11905*

*CXB348.220.14324*

*OIB354.410.36479*

*TUA368.700.57272*

*TUE384.720.151,063*

*PCA367.560.41671*

*PCB389.260.39504*

*TCA371.280.31336*

*BRO378.310.51408*

*MIZ386.270.59250*

*OIA434.630.391,273*

*WTC466.500.53826*

*OIC337.460.261,340*

*TBA422.860.51,037*

Minor comments

Q14: On page 10, line 17-19: "The insertions of LTR-RTs were more likely to occur in the dispensable genes". Authors only counted the ratio of genes with LTR-RTs in intron regions in the core, softcore, and dispensable genes in Figure 1h. Coding region and promoter region should be counted and compared.

*Response: We thank the reviewer for pointing this out, and we have revised this as suggested. In the revised manuscript, we calculated the ratio of LTR-related genes in core, softcore and dispensable genes (Fig. 1h). An LTR-related gene is defined as a gene with insertions of LTR-RTs in the regions of 2 kb upstream and downstream of the gene body.*

Q15: On Page 13, line 1-3: "We observed that nearly 50% of SVs involved repetitive sequences (Fig. 2e), and SVs were tightly associated with morphological diversification (Supplementary note)." If repetitive sequences cover 46.52% of the Chiifu genome, 50% of SVs involved repetitive sequences may be not very high. Authors should compare SV regions with genome regions selected randomly.

*Response: We agree. In the revised manuscript, we added an analysis of the comparison of TE coverages in SV regions and genome regions selected randomly, we found that the average TE coverage in the SV sequences was significantly higher than in the genomic regions selected randomly (P = 3e-312) (Page 13; Line 2–3, Fig. S11).*

*Fig. S11. Comparison of TE coverage between SV regions and genome regions selected randomly. We randomly selected 4000 SV sequences with a length of 1–2 kb, and also randomly selected 4000 genomic sequences with a length of 1–2 kb from the whole genome, and calculated the TE coverage of the two sets of sequences.*

Q16: The legend for multiple tests should be more detailed (e.g., Fig. 1e-f).

*Response: We have clarified this in the revised manuscript.*

Q17: Spelling mistake. (e.g., hist2 in Line 16 on Page 34 should be hisat2.).

*Response: We have corrected this in the revised manuscript.*

Q18: On Page 16, line 13-16: "We found that an average of 10.06% and 3.47% of two copies were least and more FSGs (Fig. S21), and an average of 7.77%, 3.35%, and 1.86% of three copies were least, more and most FSGs, respectively (Fig. 3i and Table S26)" and I didn't see Fig. 3i and I think it's Fig. 3g.

*Response: We thank the reviewer for pointing this out, We have corrected this in the revised manuscript.*

Q19: In Fig.6a, it should be better to use SV genotypes.

*Response: SV genotypes have been visualized in Fig. 6b; it was clear that a 279 bp deletion was enriched in the heading population.*

Reviewer #2:

This is a valuable study on the impacts of (allo)polyploidisation on diversification in Brassica rapa and will be of broad interest. The dataset is phenomenal in scale and the analysis appears solid, but the presentation needs some improvement. I have given many suggestions to increase clarity. Aside from issues with the writing (which I work to enumerate at length below), I do have a couple of broader concerns with the paper:

Q1: Overall the scope of this entire paper is limited to allopolyploids, but the authors uniformly use the much wider term 'polyploids' throughout. This is not accurate and overblows the import of the work: given the lack of hybridisation (and thus subgenomes), 'pure polyploids,' autopolyploids, do not engage in the phenomena discussed here (they don't have homeologs, subgenome dominance, etc). Therefore the authors should consistently change their terminology to add the prefix 'allo-' to 'polyploid' throughout. Further, the distinction between these two major types of polyploids should be made clear up front in the introduction.

*Response: We thank the reviewer for pointing this out. We have corrected and added the distinction between allopolyploidization and autopolyploidization in the revised manuscript (Page 3; Line 6–8).*

Q2: Given the amazing data it seems a missed opportunity that the authors use only SV to identify genomic correlates of morphotype diversity. Why not compare to the SNP data? If the goal is to get a realistic picture of the genomic changes controlling phenotype, looking only at SVs makes little sense and should be paired with an analysis of the SNP polymorphisms, which relative to the SV analysis, would be straightforward, especially given the authors are focussing only on the heading phenotype. The MS as currently presented leaves the reader wondering whether the given SV analysis is more powerful than one using SNPs in the same dataset and additionally how many genes are missed in the presented approach. Surely some SNPs are causative, and the SV used here are imperfect linked markers for the causative SNP. If on the other hand the given SV analysis is more powerful for finding associated genetic changes than using SNPs, that would be fascinating to see. Would it not be best to have both complementary methods, given the data are there and this is a golden opportunity to compare the normal standard with this new unique SV-based analysis? This would also give a fuller picture of the true genomic basis for the traits of interest. The authors are part-way there with the XP-EHH analysis shown over one region; can this not be formalised in addition to a SNP-based GWAS and compared to the SV results to show whether indeed this SV approach indeed finds more candidates than mapping SNPs to a single reference (or a pangenome graph, as the authors prefer)?

*Response: We agree. In the revised manuscript, we conducted domestication analysis between the heading and non-heading groups based on whole-genome SNPs, and we further compared the differences in domestication analysis using traditional SNPs and SVs.*

*Using SNP-based domestication analysis, we identified a total of 3284 genes from the 33 XP-EHH, 22 ROD, and 23 Fst outlier regions (Fig. S33, Table S38, Supplementary note). Furthermore, we compared the candidate genes identified by the SNPs with those identified by SVs, and we found that 34 of the 266 candidate genes detected by SV-based domestication analysis were presented in the candidate genes detected by SNP-based domestication analysis.*

*In our work, we found that SV-based domestication analysis was a supplement to SNP-based domestication analysis. As the reviewer mentioned, it will be better to use both complementary methods. In our previous work, we conducted SNP-based domestication analysis between the heading and non-heading populations (Cheng et al., 2016). Therefore, in the present study, we focused on SV-based domestication analysis, and we have added SNP-based domestication analysis in the revised manuscript as suggested.*

*Fig. S33. Genomic signatures of selection in the genome of Chinese cabbage. The XPEHH, ROD, and Fst values were normalized as z scores for B. rapa. A 200-kb sliding window with an increment of 5 kb was used to calculate these values; each point represents a value in a 200-kb window. The horizontal dashed lines show the empirical threshold of α = 0.01 (z = 2.33).*

*Cheng et al., 2016; Subgenome parallel selection is associated with morphotype diversification and convergent crop domestication in Brassica rapa and Brassica oleracea.*

Q3: Thirdly, the authors state (e.g. P24ln7) that 'gene flexibility during intraspecific diversification increased the subgenome dominance'. How do the authors feel causality is established in this relationship? This needs to be better stated (justified) for the reader. That is, why do they think that subgenome dominance does not cause the 'flexibility'? A reader could imagine, for example, that highly unequal expression levels of two homeologs would lead to stronger purifying selection on the highly expressed one, and much lower purifying selection on the less strongly expressed one. On account of this difference in expression, the low expressed one to accumulate diversity. This is in strong contrast to the (purported causal) 'flexibilty' the authors assume. This may be a lack of knowledge on my part, but as a reader of the document, I am not at all convinced causality is established in this assumed relationship.

*Response: The subgenome dominance in the paleohexaploid B. rapa genome we analyzed refers to the fact that one of the subgenomes (LF subgenome) is prone to retaining more genes, while the other two MF subgenomes tend to lose more genes. It is accepted that the dominance was the result of “two-step” evolution (Wang et al., 2011; Cheng et al., 2012; Tang et al., 2012). In addition to the “two-step theory”, we demonstrated that the biased gene flexibility of the different subgenome also contributed to the dominance.*

*Previously, the ‘‘two-step theory’’ was widely accepted to explain subgenome dominance in the paleohexaploid B. rapa genome (Wang et al., 2011; Cheng et al., 2012; Tang et al., 2012), which illustrated that B. rapa experienced a tetraploidization followed by substantial genome fractionation (subgenomes MF1 and MF2) and subsequent hybridization with a third, less-fractionated genome (subgenome LF). The ‘‘two-step theory’’ illustrated that the subgenome dominance in B. rapa was attributed to biased gene fractionation. However, the ‘‘two-step theory’’ cannot be used to explain biased gene fractionation during intraspecific diversification, as all three subgenomes were co-existing during this stage. In the present manuscript, we focused on analyzing the impacts of biased gene fractionation during B. rapa intraspecific diversification. We agree with the reviewer’s comment that expression dominance is associated with bias fractionation. This phenomenon was also observed in an allotetraploid cotton species (Senchina et al., 2003) and an autotetraploid species of maize (Schnable et al., 2011). In the maize genome, it was proposed that maize genotypes with deletions in the higher expressed duplicate copy of the syntenic genes were removed from the population by purifying selection, while genotypes with deletions in the lowerexpressed copy were more likely to be under near-neutral selection (Schnable et al., 2011).*

*In the present manuscript, we found a significantly lower rate of FSGs in the LF dominance subgenome than that of the MF subgenomes, and the biased gene flexibility increased the subgenome dominance. We have added the relationship between SVs and FSGs and have rephased this section in the revised manuscript. Compared to CSGs, we found that FSGs accumulated significantly higher proportion of non-synonymous mutations, large-effect mutations, SVs, and TEs (Page 14–15). Meanwhile, we observed that the average expression level of FSGs was significantly lower than that of CSGs (Fig. 3a).*

*Wang et al., 2011; The genome of the mesopolyploid crop species Brassica rapa.*

*Cheng et al., 2012; Biased Gene Fractionation and Dominant Gene Expression among the Subgenomes of Brassica rapa.*

*Tang et al., 2012; Altered Patterns of Fractionation and Exon Deletions in Brassica rapa Support a Two-Step Model of Paleohexaploidy.*

*Senchina et al., 2003; Rate variation among nuclear genes and the age of polyploidy in Gossypium. Schnable et al., 2011; Differentiation of the maize subgenomes by genome dominance and both ancient and ongoing gene loss.*

Other points:

Q4: Abstract ln7-8, "Among the genes derived from WGT, 13.42% of polyploidyderived genes accumulated more transposable elements" More than what? More than others? This is clear.

*Response: Yes, more than other genes. We have clarified this (Page 2; Line 8).*

Q5: Abstract ln17, 266 candidate genes for what? Domestication genes with SV?

*Response: Four out of 266 candidate genes for Chinese cabbage domestication. We have clarified this (Page 2; Line 17).*

Q6: P3ln6, "subgenome dominance is a common phenomenon that is widely observed in polyploids" The authors must distinguish first between allo- and autopolyploids. Subgenome dominance is not a common phenomenon across all polyploids: it is only evident in allos.

*Response: We thank the reviewer for pointing this out. We have added the distinction between allopolyploidization and autopolyploidization in the introduction (Page 3; Line 6–8) and have changed "polyploidization" into "allopolyploidization " as suggested.*

Q7: P3ln15, 'ancient' is too specific: diversification events following WGD occur within 10,000 -20,000 generations, for example, which is much less ancient than those to which the authors are perhaps pointing.

*Response: We have removed 'ancient' from the revised manuscript.*

Q8: P4ln4, '620 homoeologous pairs that have been subjected to the domestication of long white fiber' This is unclear as written. The pairs have been subjected to the domestication? Perhaps this is colloquial in crop breeding, but not broadly accessible usage.

*Response: We have clarified this in the revised manuscript, and have changed it into 'domestication analysis for long white fiber revealed 620 homoeologous pairs that have been subjected to domestication selection in the A or D subgenome, and only 34 homoeologous pairs exhibit selection signals in both subgenomes' (Page 4; Line 5–7).*

Q9: P4lLn8-11, Please be more specific: long read pop resequencing can achieve this, just not short read.

*Response: We have clarified this in the revised manuscript and have changed it to 'the combination of a single reference genome and population-scale short-read resequencing cannot be adopted to identify large structural variations and genomic sequences that are absent in the reference genome' (Page 4; Line 9–10).*

Q10: P4ln22, Most readers will have an idea of the A.thaliana genome size, but it should be stated here if the authors move away from giving percentages to giving Mb in this example (inexplicably to the reader). It would be better to keep examples consistent and use percentages, as in all the preceding.

*Response: We have added percentages as suggested (Page 5; Line 1–2).*

Q11: P5ln22 'crucial' is not the correct word here. I would suggest 'powerful' or similar. A11: We have revised this (Page 5; Line 18).

*Response: We have revised this (Page 5; Line 18).*

Q12: P6ln7 "correlations of SVs with intraspecific morphotype domestication are still unclear," or do you mean 'there are no studies of…'? if they are unclear it suggests there's some knowledge but the correlation itself is unclear, which does not seem right. Please rephrase.

*Response: We have revised this and have changed it to "there are no studies of morphotype domestication using SVs in B. rapa" (Page 6; Line 7).*

Q13: P6ln12 'extensively identified' seems rather fluffed up. Suggest striking 'extensively'.

*Response: We have revised this.*

Q14: While generally very good, sometimes the language can still be polished: 'the core gene represents'. I suggest to change that to 'core genes are defined as genes that were retained' and the parallel construction in the rest of the sentence.

*Response: We have revised this.*

Q15: Ln 16 'correlated with subgenome dominance' Do the authors mean 'the extent of subgenome dominance'?

*Response: Yes, we mean 'the extent of subgenome dominance'. We have corrected this in the revised manuscript.*

Q16: P7ln15 'for genome annotation' I suggest to strike or rephrase.

*Response: We have removed this.*

Q17: P8ln1 'Multiple' or more accurately 'All'? Also, 'variations' would be better as 'variants' here and elsewhere.

*Response: We have revised this.*

Q18: P8ln5 'relatively short period' begs the question 'how long.' It would be good to give some estimate for the reader.

*Response: We have removed this description.*

Q19: Ln9 'variations' (see above).

*Response: We have revised this.*

Q20: Table 1å: 'statistics' More accurately these are 'metrics'.

*Response: We have revised this.*

Q21: P9ln7 'closed pan-genome': for the readers who don't read a lot of pangenome literature please explain what you mean by this uncommon term more explicitly (though obviously the next sentence follows the statement).

*Response: We have explained this in the revised manuscript (Page 9; Line 8–9).*

Q22: P10ln1 what is the difference here between orphan genes and those present in only one accession? Are private genes where both homeologs are present in one accession and orphans where only one homeolog is present in one accession? If so, please make more clear for readers with even less allopolyploid orientation than myself.

*Response: Yes, orphan genes only present in one accession, and have no homologs. Orphan genes and the genes that exist in one accession and have homologs are called private genes. We have clarified this in the revised manuscript (Page 10; Line 3–4).*

Q23: P10ln5: some of the writing could still be cleaned up. For example, rephrasing "The proportion of genes with large effect mutations in core genes" to something like "The proportion of core genes with large effect mutations" etc.

*Response: We have revised this (Page 10; Line 7–8).*

Q24: P11ln14 here, for example, 'variations' should just be 'variation'.

*Response: We have revised this (Page 11; Line 15).*

Q25: P11ln16 'in different chromosomes and the whole-genome' simplify to something like 'across the genome'.

*Response: We have revised this (Page 11; Line 17–18).*

Q26: P12ln6 'Large-scale resequencing illustrated the high representation of identifying genomic variations using the B. rapa pan-genome' Please rephrase completely; this makes little sense to the reader.

*Response: We have changed this to 'There is a strong correlation between the variants identified by large-scale resequencing accessions and the B. rapa pan-genome’ (Page 12; Line 7–8).*

Q27: P12ln16 'further supporting the high representation of identifying genomic variations using the pan-genome' Totally unclear to me what this means here.

*Response: We have changed this to ' further revealing that the pan-genome harbored abundant variants of B. rapa’ (Page 12; Line 17–18).*

Q28: P13ln1 'species' I am no expert on Brassicas, but is it right to call these different species of the species Brassica rapa?

*Response: We have change this to 'different B. rapa genomes' in the revised manuscript (Page 13; Line 2).*

Q29: P13ln4 'the nonredundant SV set started to slow down and tended to flatten (Fig. 2f)' Something is missing from this sentence. Please add something like 'with the addition of more species. I note 'started to slow down and tended to flatten' is a bit strange usage here.

*Response: We agree with this comment. In the revised manuscript, we changed this to 'the addition of more sequenced genomes did not affect the size of the nonredundant SV set' (Page 13; Line 5–6).*

Q30: P14ln10: this section heading is not proven by the following text. It's a nice speculation but not appropriate as a section heading. In what way does the study test if anything is a 'driving force'?

*Response: We agree with this comment. In the revised manuscript, we changed this to 'the flexibility of syntenic genes was associated with the intraspecific diversification of B. rapa' (Page 14; Line 11–12).*

Q31: P14ln13 “The gene flexibility represented a gene was fractionated in some B.rapa genomes." Please rephrase; this makes little sense to the reader.

*Response: We have removed the sentence in the revised manuscript.*

Q32: Ln16 'In this study, if a gene had an orthologous gene in A. thaliana' or more simply 'an ortholog in A. th..'

*Response: We have revised this (Page 14; Line 16–18).*

Q33: P15ln9 nonsyn and large effect mutations do not equal positive selection and the inference of causality ('driving force' in the section heading) in this section is unsupported. Please rephrase.

*Response: We have changed the section heading to ' The flexibility of syntenic genes was associated with the diversification of different B. rapa genomes' (Page 14; Line 11–12).*

Q34: Ln 10: in what way exactly do the authors mean, strictly speaking, that 'FSGs were strongly correlated with the intraspecific diversification'? This entire section should be reworked.

*Response: We rephased this section and changed the section title to ' The flexibility of syntenic genes was associated with the diversification of different B. rapa genomes’ (Page 14; Line 11–12). The main results were as follows: First, we identified 3,324–4,575 FSGs in each of the 18 B. rapa genomes, and 13.42% of WGT-derived genes were flexible among the different B. rapa genomes. Second, we found that the average expression level of FSGs was significantly lower than CSGs. Third, compared to CSGs, we found that FSGs accumulated significantly higher proportions of non-synonymous mutations, SVs, and TEs. These results supported the association of FSGs with the diversification of different B. rapa genomes.*

Q35: P15ln16: it is not enough to bring up 'the two-step theory' as the authors do: there should please be a brief description of what they're talking about here.

*Response: We have added this in the revised manuscript. The ‘‘two-step theory’’ suggests that B. rapa experienced a tetraploidization followed by fractionation and subsequent hybridization with a third genome, which shows less fractionation (Page 15; Line 16–20).*

Q36: P15ln21: Fig S24 callout here is an error?

*Response: We have revised this.*

Q37: Here also: 'revealing that the biased gene flexibility during intraspecific diversification increased the dominance of the LF subgenome' The authors cannot prove causality here and are simply measuring a correlation. This should be rephrased accordingly throughout.

*Response: The subgenome dominance in the paleohexaploid B. rapa genome we analyzed refers to the fact that the LF subgenome is prone to retaining more genes, while the other two MF subgenomes tend to lose more genes. Additionally, the ‘‘two-step theory’’ illustrated that the subgenome dominance in B. rapa was attributed to biased gene fractionation (Wang et al., 2011; Cheng et al., 2012) (for details please refer to A3). In the present manuscript, a significantly lower rate of fractionated genes was observed in the dominant LF subgenome during intraspecific diversification. Therefore, we concluded that biased gene flexibility during intraspecific diversification increased the subgenome dominance.*

Q38: P16ln3: 'further highlighting the continuing influence of the dominant subgenome on intraspecific diversification.' I do not see how causality is proven here and therefore the authors need to either explain this clearly or change their language to state correlation instead.

*Response: We agree with this comment. As suggested, we have changed this to 'further highlighting the continuing influence of biased gene fractionation during intraspecific diversification' (Page 16; Line 6–7).*

Q39: P16ln17 does gene duplication actually 'reduce the pressure of effective purifying selection'? This seems an odd formulation. That is, the selection pressure is the same; I'd just see it as managed differently by the duplicates.

*Response: We have removed this.*

Q40: P17ln5 use of parenthesis is awkward such as 'Conserved (Flexible) represent conserved (flexible) syntenic gene in the homoeologous pair.' Rephrase please.

*Response: We have changed this to 'Conserved' and 'Flexible' represent conserved and flexible syntenic genes (Page 17; Line 7).*

Q41: P17ln15 "In total, there were 30,166 genes in the B. rapa ancestral" The language here is too careless: there were 30,166 genes in the inferred B. rapa ancestral genome. The number in the actual ancestral genome is unknown. Please rephrase with more care. Later invocations (final line on page/first line of p18, for example) should also be given the adjective 'inferred' to 'ancestral.'

*Response: We have added the adjective 'inferred' as suggested.*

Q42: Ln18 'revealing' The language is a too strong and somewhat careless. Suggest 'indicating' or 'suggesting'. The evidence given is hardly conclusive enough to 'reveal'.

*Response: We have changed 'revealing' into 'indicating' (Page 18; Line 2).*

Q43: P18: Please describe exactly what 'gene density' measures and how it is calculated at the first mention.

*Response: We have calculated gene densities in the three subgenomes to investigate biased gene fractionation. Additionally, we added the method to calculate gene densities in the method (Page 35; Line 19–21).*

Q44: P18ln4-7: two sentences merged in run-on. Rephrase.

*Response: We have revised this (Page 18; Line 9).*

Q45: P18ln11: delete 'the' in 'the B. nigra'.

*Response: We have revised this (Page 18; Line 15).*

Q46: P18: again, the discussion here focusses on INFERRED ancestral genomes and the gene numbers are low estimate for the ACUTAL gene numbers in the ancestral genomes. The language in the present draft does not take this into account and needs to be clearer throughout.

*Response: We have added this in the revised manuscript.*

Q47: Fig5: spell out R. sativus genus please.

*Response: We have corrected this (Page 20; Line 4).*

Q48: P20ln13; FigS22 shows a single example re the 74 bp deletion tested, but Table S31 gives many primer pairs. None of the other pairs or data are described. Why not?

*Response: As the selected SVs were all independently tested using PCR, only an example is provided to avoid excessive supplementary material. For the convenience of other researchers, we have provided the primer sequences used in our experiments. In addition, we will upload this information and the genomic data to the public database BRAD (http://brassicadb.cn).*

Q49: P20ln17 "especially for the leaf heading…" rephrase; this makes no sense.

*Response: We have removed this.*

Q50: P24ln9 "the genomes became more different" When? Please rephrase and clarify.

*Response: We have changed this to "the subgenomes became more different in gene content and the rate of accumulating genomic variants during intraspecific diversification” (Page 24; Line 8–10).*

Q51: The discussion (like the entire paper) is very long and should be trimmed dramatically for readability. Much of it is redundant with previous statements in the manuscript.

*Response: We have polished the discussion and the entire paper by removing redundant descriptions in the discussion and have also rephased unclear sentences.*

Q52: P24ln16 when stating a sentence spell the genus; here it's abbreviated.

*Response: We have revised this (Page 24; Line 16).*

Q53: P25Ln12 why TE's all of a sudden? The discussion seems rambling and a bit disorganized.

*Response: We have refined the discussion and have removed this sentence from the revised manuscript.*

**Second round of review**

**Reviewer 1**

I have couple of minor suggestions:

The characters in several figures are very small and not clear. For example, in Figure 1 c-h. The size of the font should be increased.

Page 2 line 13 and page 13 line 13, LF subgenome has not been defined for the first use in the abstract and the context

Page 2 line 5, we report the consequences, I do not think "on" should be included

Page 3 line 20, but how exactly this diversity contributed to the domestication needs to

Page 4 line 12, I suggest change to "single reference genome is not sufficient to"

Page 9 line 6, reference should be cited for two published reference genomes

Page 25 line 8, In both stages

Page 27 line 18, it is better not to include "our"

**Reviewer 2**

I am satisfied with the revised manuscript.

**Authors Response**

**Point-by-point responses to the reviewers’ comments:**

*Response: We have addressed concerns from Referee 1 and the editor.*
